# Study protocol for two randomised controlled trials evaluating the effects of Cerclage in the reduction of extreme preterm birth and perinatal mortality in twin pregnancies with a short cervix or dilatation: the TWIN Cerclage studies

**DOI:** 10.1136/bmjopen-2023-081561

**Published:** 2024-05-10

**Authors:** Lissa van Gils, Marjon A de Boer, Judith Bosmans, Ruben Duijnhoven, Sam Schoenmakers, Jan B Derks, Jelmer R Prins, Salwan Al-Nasiry, Margo Lutke Holzik, Enrico Lopriore, Joris van Drongelen, Marieke H Knol, Judith O E H van Laar, Yves Jacquemyn, Caroline van Holsbeke, Isabelle Dehaene, Liesbeth Lewi, Hannes van der Merwe, Wilfried Gyselaers, Sylvia A Obermann-Borst, Mayella Holthuis, Ben W Mol, Eva Pajkrt, Martijn A Oudijk

**Affiliations:** 1 Department of Obstetrics and Gynaecology, Amsterdam UMC, Amsterdam, The Netherlands; 2 Department of Health Sciences, Amsterdam UMC Location AMC, Amsterdam, Noord-Holland, The Netherlands; 3 Department of Obstetrics and Gynaecology, Erasmus Medical Center, Rotterdam, The Netherlands; 4 Department of Obstetrics, University Medical Centre Utrecht, Utrecht, The Netherlands; 5 Department of Obstetrics and Gynaecology, University Medical Center Groningen, Groningen, The Netherlands; 6 Department of Obstetrics and Gynaecology, Maastricht UMC+, Maastricht, The Netherlands; 7 Department of Obstetrics and Gynecology, Leiden Universitair Medisch Centrum, Leiden, The Netherlands; 8 Department of Neonatology and Fetal Medicine, Leiden University Medical Center, Leiden, The Netherlands; 9 Department of Obstetrics and Gynecology, Radboud Universiteit, Nijmegen, The Netherlands; 10 Department of Obstetrics and Gynaecology, Isala Zwolle, Zwolle, The Netherlands; 11 Department of Obstetrics & Gynaecology, Máxima Medical Center, Veldhoven, The Netherlands; 12 University Hospital Antwerp, Antwerpen, Belgium; 13 AZ Sint-Lucas Brugge, Brugge, West-Vlaanderen, Belgium; 14 University Hospital Ghent, Gent, Belgium; 15 Fetal Medicine Unit, KU Leuven University Hospitals Leuven, Leuven, Flanders, Belgium; 16 Department of Obstetrics and Gynecology, Hospital Oost-Limburg, Genk, Belgium; 17 Care4Neo, Neonatal patient and parent advocacy organization, Rotterdam, The Netherlands; 18 Patient organization 'Extreme Vroeggeboorte', Amsterdam, The Netherlands; 19 department of obstetrics and gynaecology, school of medicine, Monash University, Melbourne, Victoria, Australia

**Keywords:** fetal medicine, maternal medicine, obstetrics

## Abstract

**Introduction:**

Twin pregnancies have a high risk of extreme preterm birth (PTB) at less than 28 weeks of gestation, which is associated with increased risk of neonatal morbidity and mortality. Currently there is a lack of effective treatments for women with a twin pregnancy and a short cervix or cervical dilatation. A possible effective surgical method to reduce extreme PTB in twin pregnancies with an asymptomatic short cervix or dilatation at midpregnancy is the placement of a vaginal cerclage.

**Methods and analysis:**

We designed two multicentre randomised trials involving eight hospitals in the Netherlands (sites in other countries may be added at a later date). Women older than 16 years with a twin pregnancy at <24 weeks of gestation and an asymptomatic short cervix of ≤25 mm or cervical dilatation will be randomly allocated (1:1) to both trials on vaginal cerclage and standard treatment according to the current Dutch Society of Obstetrics and Gynaecology guideline (no cerclage). Permuted blocks sized 2 and 4 will be used to minimise the risk of disbalance. The primary outcome measure is PTB of <28 weeks. Analyses will be by intention to treat. The first trial is to demonstrate a risk reduction from 25% to 10% in the short cervix group, for which 194 patients need to be recruited. The second trial is to demonstrate a risk reduction from 80% to 35% in the dilatation group and will recruit 44 women. A cost-effectiveness analysis will be performed from a societal perspective.

**Ethics and dissemination:**

This study has been approved by the Research Ethics Committees in the Netherlands on 3/30/2023. Participants will be required to sign an informed consent form. The results will be presented at conferences and published in a peer-reviewed journal. Participants will be informed about the results.

**Trial registration number:**

ClinicalTrials.gov, NCT05968794.

**STRENGTHS AND LIMITATIONS OF THIS STUDY**

- These are the largest randomised trials comparing the effects of cerclage on preterm birth (PTB) in women with a twin pregnancy and a short cervix or dilatation.
- A strength of the study lies in its comprehensive assessment of outcomes, including PTB at various gestational age thresholds, as well as a thorough examination of adverse maternal and neonatal outcomes.
- The intention to perform long-term follow-up of children, so that future parents can be counselled on the long-term effects of this intervention, enhances the strength of this study.
- The recruitment of patients could be a potential challenge as the incidence of women with a twin pregnancy and a short cervix of ≤25 mm or dilatation at midpregnancy is low.

## Introduction

Twin pregnancies have a high risk on extreme preterm birth (PTB) at <28 weeks of gestation, which is associated with increased risk of neonatal morbidity and mortality.^1^ Worldwide, twins account for 0.5–4.0% of all births, with rates varying between ethnic groups and countries.^2^ Compared with singletons, twins are at increased risk for adverse perinatal outcomes.^3^ In the Netherlands, 250 (5,2%) women with a twin pregnancy deliver at <28 weeks per year, resulting in 157 perinatal deaths and 343 surviving neonates of whom a large proportion suffer from long-term neurodevelopmental problems associated with extreme PTB.^4^ An asymptomatic short cervix (≤ 25 mm) or dilatation at midpregnancy in twin pregnancies is associated with high rates of extreme PTB and proven to be the best predictor for extreme PTB.^5 6^

Effective interventions for twin pregnancies with short cervix are limited. The Evaluating progestogens for preventing Preterm birth International collaborative individual participant data (IPD) meta-analysis on progesterone treatment in multiple pregnancies with a short cervix showed no reduction of PTB; however, a recent IPD meta-analysis showed a possible effect of progesterone.^7 8^ The use of a vaginal pessary in women with a twin pregnancy and a short cervix does not seem to result in a reduction of PTB.^9^

A possible surgical method to reduce extreme PTB in twin pregnancies with an asymptomatic short cervix or dilatation at midpregnancy is a vaginal cerclage. An IPD meta-analysis from 2015 assessed its impact on 49 women with a twin pregnancy and a short cervix (<25 mm) across three randomised controlled trials (RCTs).^10^ No significant differences were present between the groups on outcomes such as PTB or perinatal death. However, a more recent systematic review with a total of 471 women also included six cohort studies that focused on the outcome of PTB at different cut-offs.^11^ The pooled results of these cohort studies showed a significant beneficial effect of a cerclage on PTB at <32 weeks (relative risk (RR) 0.68; 95% CI 0.51 to 0.92) and <34 weeks (RR 0.73; 95% CI 0.59 to 0.90). In addition, a cerclage significantly prolonged pregnancy (mean 2.53 weeks (95% CI 1.25 to 3.81)). No significant difference was seen in the number of PTBs at <24 and 28 weeks or perinatal mortality, although the trend was in favour of the cerclage group.

Furthermore, a subgroup analysis by cervical length was carried out, distinguishing the cervical length between 16 and 24 mm and ≤15 mm.^11^ For the group with a cervical length of <15 mm, cerclage placement was associated with fewer PTBs at <37 weeks (RR 0.86; 95% CI 0.74 to 0.99), <34 weeks (RR 0.57; 95% CI 0.43 to 0.75) and<32 weeks (RR 0.61; 95% CI 0.41 to 0.90) compared with the control group. Cerclage also resulted in a significant pregnancy prolongation by 3.89 weeks (95% CI 2.19 to 5.59). In the group of women with a cervical length of 16–24 mm, no significant differences were seen in the outcomes.

In women with a twin pregnancy and asymptomatic cervical dilatation, the most recent systematic review was published in August 2023 and included one RCT and five retrospective cohort studies.^12^ These studies showed that a vaginal cerclage may result in a reduction of PTB at less than 24 weeks (RR 0.32; 95% CI 0.21 to 0.48), 28 weeks (RR 0.47; 95% CI 0.36 to 0.62), 32 weeks (RR 0.59; 95% CI 0.50 to 0.70) and 34 weeks (RR 0.68; 95% CI 0.57 to 0.80). In addition, women with a cerclage had a reduced risk of perinatal death (RR 0.30; 95% CI 0.16 to 0.55), 5 min Apgar score of <7 (RR 0.49; 95% CI 0.27 to 0.90), birthweight of <1500 g (RR 0.41; 95% CI 0.31 to 0.55) and admission to the neonatal intensive care unit (NICU) (RR 0.67; 95% CI 0.52 to 0.88).

In conclusion, there is a lack of well-designed RCTs on the effect of vaginal cerclage on PTB and neonatal outcomes in women with a twin pregnancy with a short cervix or asymptomatic dilatation at midpregnancy. Long-term follow-up of children is lacking in general; thus, future parents cannot be counselled on the long-term effects of this intervention. The possible effect of a cerclage in cohort studies justifies a well-designed large multicentre randomised trial. We therefore will perform the TWIN cerclage study.

The objective of the present study is to assess the effectiveness of a vaginal cerclage in pregnant women with a twin who had a midpregnancy asymptomatic short cervix or dilatation in the prevention of extreme PTB and perinatal mortality compared with the standard treatment (no cerclage).

## Methods and analysis

### Design and setting

We will conduct two multicentre RCTs within the Dutch Consortium for Healthcare Evaluation and Research in Obstetrics and Gynaecology (eight hospitals). Sites in other countries may be added at a later date.

### Participants and eligibility criteria

RCT I: Women (>16 years of age) with a twin pregnancy with a short cervix of ≤25 mm below 24 weeks of pregnancy are eligible for this trial.

RCT II: Women (>16 years of age) with a twin pregnancy with dilatation on transvaginal ultrasound or physical examination below 24 weeks of pregnancy are eligible for this trial.

Women who meet any of the following criteria will be excluded from participation in these trials:

- Women with a mono-amniotic twin pregnancy.
- Women with twin pregnancy in which one or both children are diagnosed with a major structural, congenital or chromosomal abnormality (at time of inclusion) that is likely to influence the composite adverse neonatal outcome.
- Women with signs of clinical intrauterine infection, defined by the presence of fever at ≥38°C.
- Women with overt symptoms of preterm labour at the time of measurement of short cervix or dilatation (regular contractions, premature rupture of membranes (PPROM), recurrent blood loss).
- Women with a placenta previa, defined as a placenta position covering the internal ostium of the cervix.
- Women who do not master the Dutch or English language and are therefore not able to give written consent.

### Sample size calculation

In women with a twin pregnancy and an asymptomatic short cervix, the sample size was calculated based on a meta-analysis published in 2019.^11^ In this study, the incidence of PTB before 28 weeks was lower in the cerclage group (13.8%) compared with the expected management group (23.0%) (RR 0.64; CI 0.40 to 1.02).

- Stratum I short cervix group: To demonstrate a risk reduction from 25% to 10%, 97 patients need to be recruited per arm (194 in total). The sample size for this stratum was calculated using a Z-test with unpooled variance, a two-sided alpha of 0.05 and a target power of 80%.

In women with a twin pregnancy and asymptomatic cervical dilatation, the sample size was calculated based on a systematic review published in December 2021, which included one RCT and four retrospective cohort studies.^13^ In this study, the incidence of PTB before 28 weeks was significantly lower in the cerclage group (37.2%) compared with the expected management group (76.9%) (RR 0.44; CI 0.33 to 0.58).

- Stratum II dilatation group: To demonstrate a risk reduction from 80% to 35%, 22 patients need to be recruited per arm (44 in total). The sample size for this stratum was calculated using Fisher’s exact test, with a two-sided alpha of 0.05 and a target power of 80%. Numbers are not corrected for loss of follow-up, as it is highly unlikely to have loss to follow-up in these high-risk pregnancies.

### Treatment

The investigated intervention is a vaginal cerclage. Potential complications of a cerclage are infection, premature rupture of membranes, cervical laceration or bleeding and anaesthesia-related complications, occurring in approximately 0.3–2.5%.^14^

The comparator will be standard treatment according to the current guideline in the Netherlands from 2018, which is to not perform or offer an intervention such as vaginal cerclage.^15^

### Study procedures

Measurement of cervical length in twin pregnancies will be performed as part of the standard 20-week ultrasound scan in all hospitals in the Netherlands. In case of a short cervical length or dilatation, patients will be seen at the nearest participating centre, where women will be counselled for participation in the study. Inclusion is possible below 24 weeks of gestation, and if randomised to the surgical treatment, the cerclage needs to be placed before 24^+0^ weeks of gestation.

After women give written consent to participate in the trials, patients will be randomised for two treatment options: (1) surgical treatment with cerclage and additional standard care according to Dutch guidelines or (2) standard care according to Dutch guidelines (no cerclage). Randomisation will be done using a web-based interface for each of the two trials. For the trial recruiting women with dilatation, random permuted blocks will be used of sizes 2 and 4 to minimise the risk of disbalance. Participants and investigators will not be blinded for the intervention.

In addition, participants are treated according to the local protocol in the participating clinics, that is, the use of progesterone during pregnancy and other interventions such as tocolysis and corticosteroids in case of a threatened PTB. Follow-up of the pregnancy and delivery will be in the referral centre until delivery. The cerclage will be removed according to the protocol between 36 and 37 weeks of gestation, or in case of labour, whatever comes first. Delivery will take place by either spontaneous onset of labour or induction of labour or elective caesarean section according to national guidelines for twin pregnancies.

The referral hospitals will be contacted for data collection of outcomes as mentioned above by the studies’ research midwives or nurses. Primary and secondary outcome measures will be obtained from clinical patient data. Outcomes such as PTB will be assessed at birth, and additional outcomes will be assessed at the time of discharge of the child from the hospital to the home address and at 3 months after discharge.

### Outcome measures

#### Main study parameter/endpoint

The primary outcome will be extreme PTB at <28 weeks.

#### Secondary study parameters/endpoints

Secondary outcomes include:

- A composite for adverse neonatal outcomes (including bronchopulmonary dysplasia, periventricular leucomalacia of grade >1, intraventricular hemorrhage of grade >2, necrotising enterocolitis of stage >2, retinopathy of prematurity of stage >2, proven sepsis and perinatal death).
- All components of the composite outcome will also be assessed separately.
- PTB (indicated and spontaneous) at less than 24, 32, 34 and 37 weeks.
- PPROM.
- Gestational age at delivery.
- Days on ventilation support.
- Days in NICU.
- Maternal quality of life.
- Maternal outcomes, including sepsis, need for antibiotics, need to remove cerclage in the operating room and mode of delivery (% caesarean delivery).
- Healthcare costs.

Outcome parameters are in line with the core outcome set for studies on the prevention of PTB defined by members of GONet and the Core Outcomes in Women’s health initiative.^16^

Parents will be asked for consent to approach for long-term follow-up of the children aged 2 and 4 years. Maternal quality of life will be assessed at inclusion, discharge after birth and 3 months postpartum using the five-level version of the EuroQol (EQ-5D-5L).^17^ Costs will be measured from a societal perspective using web-based questionnaires based on the iMCQ and iPCQ (Medical and Productivity Cost Questionnaires) at discharge after birth and at 3 months after discharge for the mother and the neonate.

In addition, pregnancy outcome data will also be collected for women opting not to participate in the trials but giving informed consent to collect data on the pregnancy outcome from hospital records. Women give permission for this voluntarily.

### Randomisation and treatment allocation

Randomisation will be done using a web-based interface by a member of the project team or a qualified research nurse. Randomisation will be stratified for the presence of dilatation at the time of recruitment. Allocation will be 1:1, and permuted blocks sized 2 and 4 will be used of to minimise the risk of disbalance. Participants and investigators will not be blinded for the intervention.

### Withdrawal

Participants can leave the study at any time for any reason if they wish to do so, without any consequences. Patients who withdraw from the study will remain in their treatment group for the intent-to-treat analysis, and their data will be used for analysis. The investigator can decide to withdraw a participant from the study for urgent medical reasons.

### Follow-up of participants withdrawn from treatment

As the statistical analysis is planned according to the intention-to-treat principle, patients who discontinue the intervention will be analysed in the group that they were allocated to.

### Safety reporting

#### Adverse events (AEs)

An adverse event is defined as an event during or following medicinal treatment or follow-up which was not intended to happen and is suspected to be a complication of the intervention performed.

#### Serious adverse events (SAEs)

SAEs will be reported from the first study-related procedure until 3 months after delivery. A serious adverse event is any untoward medical occurrence or effect that:

- Results in death (maternal or perinatal)
- Is life threatening (at the time of the event)
- Requires hospitalisation or prolongation of existing inpatients’ hospitalisation
- Results in persistent or significant disability or incapacity
- Any other important medical event that did not result in any of the outcomes listed above due to medical or surgical intervention but could have been based on appropriate judgement by the investigator

SAEs must be reported by e-mail to the study project team within 24 hours after the event was reported to the investigator, using the provided SAE report form. This initial report should minimally contain information with respect to the event, associated treatment and patient identification, as described in the detail in the instructions for the SAE report form. If necessary, more detailed information should be provided in a follow-up report within a further two business days. SAEs need to be reported until the end of the study as defined in the protocol. SAEs that result in death or are life-threatening should be reported immediately with a maximum of 7 days for a preliminary report with another 8 days for completion of the report. All other SAEs will be reported within a maximum period of 15 days after the sponsor initially received knowledge of the serious adverse events.

If required by national laws or regulations or by the procedures of the authorities, the sponsor will ensure that a 6 monthly line listing of all reported SAEs is submitted to the ethics committee(s).

#### Events inherent to the study population

Because the study population is pregnant with a notable risk of premature birth, events inherent to the study population are to be expected and will not be considered as SAE. The study project group will report these events by line listing to the health authorities, competent authorities and research ethics committee once a year.

The following events are not considered SAE:

- Maternal hospital admission during pregnancy, delivery or postpartum (exception admission to the intensive care unit or coronary care unit, which will be considered as SAEs).
- Maternal hospitalisation for a procedure that was planned before study participation (ie, before registration or randomisation). This should be recorded in the source documents.
- Prolonged maternal hospitalisation for a complication of such procedures remains a reportable SAE.
- NICU admission.
- Neonatal hospital admission other than the NICU.
- Neonatal disease expected in prematurity such as bronchopulmonary dysplasia, periventricular leucomalacia, intraventricular haemorrhage, necrotising enterocolitis, retinopathy of prematurity and culture-proven sepsis.

#### Follow-up of SAEs

AEs occurring from the first study-related procedure until 3 months after delivery will be recorded in the CRF. All AEs will be followed until they have abated or until a stable situation has been reached. Depending on the event, follow-up may require additional tests or medical procedures as indicated and/or referral to the general physician or a medical specialist. All serious adverse events will be followed clinically until they are resolved or a stable situation has been reached. Depending on the event, follow-up may require additional tests or medical procedures as indicated and/or referral to a general physician or a medical specialist. Follow-up information on SAEs should be reported until recovery or until a stable clinical situation has been reached. Theoutcome of the SAE should be reported in the final SAE report.

#### Monitoring and safety

An independent data safety monitoring board (DSMB) will monitor the trials for safety. The DSMB consists of several members with expertise in the relevant fields of obstetrics, neonatology, epidemiology and statistics. SAEs will be collected from the first study-related procedure until 3 months after delivery. SAEs must be reported by e-mail to the study project team within 24 hours after the event was reported to the investigator.

An interim analysis will be conducted for safety (ie, interim safety review) at least after the outcomes for the first 50 and 100 patients have become available in RCT I (short cervix) or the first 22 patients (half the planned sample size) in RCT II (dilatation group).

#### Premature termination of the study

The DSMB can advise to terminate the study prematurely in case of safety concerns. The DSMB can advise to terminate the study prematurely in case the interim analysis shows harm of either one of the interventions (cerclage or no cerclage) or due to external evidence.

An interim analysis will be conducted for safety (ie, interim safety review) after the outcomes for the first 50 patients have become available for stratum I, and the first 22 patients (half the planned sample size) in stratum II. In addition, after the outcomes of 100 patients have become available from stratum I, the DSMB will meet again for an interim safety review.

### Statistical analysis

Data analysis will be performed according to the intention-to-treat principle. A supplementary per-protocol analysis is considered if any patients in the standard care arm would have received a cerclage (protocol deviation). Categorical variables will be expressed as a number with the percentage of the total allocation arm. Continuous variables will be presented as means with SDs, as geometric mean with 95% CI or as medians with IQRs, whenever appropriate.

The primary outcome will be evaluated using the intention-to-treat population. Estimand for both RCT I and RCT II is the RR for extreme PTB after cerclage, as compared with the control group not undergoing cerclage. CI will be a two-sided 95% interval, with statistical inference based on the Chi-squared test for RCT I, and Fisher’s exact test for RCT II. Heterogeneity of treatment effect is anticipated to be substantial for the two trials, and a pooled estimate will only be provided if heterogeneity is low. Heterogeneity will be assessed using a forest plot and tests for interaction in a generalised linear model. The two trials may be reported separately if not completed within a reasonable time from each other to allow timely communication of results.

Missing data for the primary outcome will be very low, given that the primary outcome is both an outcome recorded for routine care and there is a short follow-up time. If patients would deliver in other hospitals than the hospital they were recruited from, the data can still be obtained. Only patients who actively revoke consent cannot be analysed for the primary outcome.

The secondary outcome measures are approached similarly to the primary outcome measure. Dichotomous outcomes will be presented as RRs and absolute with 95% CIs and chi-squared tests or Fisher’s exact test. For continuous secondary outcomes, differences between groups will be assessed with the Student’s t-test if the outcome is normally distributed and with a non-parametric Mann–Whitney U-test if skewed. These outcomes will be presented as means with SD, geometric means with 95% CI or as medians with IQR, whichever is appropriate. A nominal level of 5% will be used for significance. From the perspective of type I error control, the two trials are considered independent tests.

### Subgroup analyses

The following subgroup analyses are planned:

- Cervical length of ≤15 mm and 16–25 mm
- Dichorionic and monochorionic pregnancies
- Multiparous women with a history of PTB and women without a history of PTB
- Nulliparous women and multiparous women with a term birth in their obstetric history

### Economic evaluation

Both a cost-effectiveness analysis and a cost-utility analysis will be performed from a societal and healthcare perspective according to Dutch guidelines with a time horizon of 3 months after discharge. All statistical analyses will be done according to the intention-to-treat principle. Missing cost and effect data will be imputed using multiple imputations according to the MICE algorithm. Rubin’s rules will be used to pool the results from the different multiply imputed datasets. Linear regression analyses will be used to estimate cost and effect differences between the intervention and control groups while adjusting for confounders if necessary. Incremental cost-effectiveness ratios (ICERs) will be calculated by dividing the difference in the mean total costs between the treatment groups by the difference in mean effects between the treatment groups. Bias-corrected and accelerated bootstrapping with 5000 replications will be used to estimate 95% CIs around the cost differences and statistical uncertainty surrounding the ICERs. Uncertainty surrounding the ICERs will be graphically presented on cost-effectiveness planes. Cost-effectiveness acceptability curves will also be estimated showing the probability that the intervention is cost-effective in comparison with control for a range of different ceiling ratios thereby showing decision uncertainty.^18^

### Budget impact analysis

A budget impact analysis (BIA) will be conducted from the perspective of healthcare decision-makers according to the Dutch guidelines and the recommendations from Sullivan *et al* using the Dutch BIA tool.^19^ In the BIA, data from the effectiveness and cost-effectiveness analyses regarding the differences in costs and health outcomes will be combined with national prevalence and incidence data to extrapolate the findings to a time horizon of 5 years. The BIA will be conducted from the government (Budget Kader Zorg) and societal perspectives. Actual NZA tariffs will be used to calculate costs. The budget analysis will differentiate between incidental and structural costs (savings) and will take into account the budgetary consequences of changes within these cost components. Sensitivity analyses will be performed for subgroups of patients if applicable, providing budget information for relevant subgroups to decision-makers. In addition, sensitivity analyses will address the impact of variations in the main assumptions and input parameters for the BIA.

### Handling and storage of data and documents

Data will be collected using Castor EDC. Data monitoring will be done by the primary investigator. Data processing will be coded, as the patient code only available to the local investigators. All data will be stored for 15 years in a secured database on a secured computer, to which only the investigators will have access. All personal data are protected conforming to the rules of the General Data Protection Regulation. Data on ethnicity and educational level will be collected from the patients’ medical files because this is one of the known risk factors for PTB. The participant will give her consent for this on the consent form (online supplemental file 1).

10.1136/bmjopen-2023-081561.supp1 Supplementary data

### Monitoring and quality assurance

Data monitoring will be performed by a certified clinical research associate of the participating institute or its delegate. The monitor will compare the data entered into the database with the hospital or clinic records (source documents). The nature and location of all source documents will be identified to ensure that all sources of original data required to complete the database are known to the investigational staff and are accessible for verification. The frequency of monitoring will be further discussed with the monitoring party.

### Patient and public involvement

The Dutch consortium collaborates with three Dutch patient associations, Care4Neo (neonatal patient and parent advocacy organisation), the ‘Vereniging voor Ouders van Meerlingen’ (society of parents of multiples) and the patient organisation for extreme PTB. They are project members of the TWINC Cerclage study and are involved in the study design. A survey was performed during the study design among members of the closed Facebook group of both patient associations to evaluate the interest in participation in the TWIN Cerclage study. The Dutch consortium has a website where it publishes all results of completed studies and protocols of currently recruiting studies. Participants will be informed about the results.

### Ethics and dissemination

The research ethics committee at the Amsterdam University Medical Centres approved this study (reference number: 2022.0885 NL82609.018.22). Additional regional approval was obtained for the remaining participating hospitals in the Netherlands. If other study sites are added, ethical approval will be required as relevant. Protocol amendments will be communicated to relevant parties.

### Recruitment and consent

Eligible participants will receive the participant information leaflet and will be informed about the study during an outpatient clinic (online supplemental file 1). Because of the need to initiate treatment as soon as possible (<24 weeks of pregnancy), we will call the participant in 1 to 5 days (based on her gestational age at the time of counselling) to get the opportunity to ask questions if she wants to participate in the study. If the participant wants to participate, she will be asked to sign the informed consent and ask to send a photo of her signed informed consent. To schedule the cerclage placement, randomisation will be performed after receiving a copy of the informed consent. If women are randomised to the cerclage group, they will be asked to bring the original signed informed consent on the day of their admission for the cerclage. If women randomise for the standard care, they will be asked to send the original signed informed consent back with the return envelope.

### Public disclosure and publication policy

The study results will be published in peer-reviewed, indexed journals with an open-access policy, and the results will be presented at academic conferences. A separate manuscript will be prepared reporting the cost-effectiveness analysis. Authorship eligibility will be in accordance with The International Committee of Medical Journal Editors. Participants will be informed about the results.

### Study status

Participants are currently being recruited and enrolled (start date: 16-05-2023). The estimated completion is 01-12-2028.

## Supplementary Material

Reviewer comments

Author's manuscript

## Data Availability

All data relevant to the study are included in the article or uploaded as supplementary information.
