# Supplementary material for: Study protocol for two randomised controlled trials evaluating the effects of Cerclage in the reduction of extreme preterm birth and perinatal mortality in twin pregnancies with a short cervix or dilatation: the TWIN Cerclage studies
Source: BMJ Open. 2024 May 10;14(5):e081561. doi: 10.1136/bmjopen-2023-081561 (PMC11097875; doi:10.1136/bmjopen-2023-081561)
Supplement: Supplementary data [file bmjopen-2023-081561supp001.pdf]

# Patient Information – TWIN cerclage study

**The TWIN Cerclage Study**: *The effectiveness of Cerclage for the reduction of extreme preterm birth and perinatal mortality in Twin pregnancies with a short cervix.*

Dear Madam,

You are asked to participate in a medical scientific study. Participation is voluntary. Your written consent is required to participate. You are receiving this letter because you are pregnant with twins and are at risk of having a preterm delivery. You can read about the medical study in this information sheet, what it means for you, and about the advantages and disadvantages. It's a lot of information. Would you please read the information and decide if you want to participate? If you would like to participate, please fill out the form found in Appendix C.

**Ask your questions**

You can make your decision with the information you find in this information letter. In addition, we encourage you to:

- Ask the researcher giving you this information any questions you have.
-Talk to your partner, family or friends about this study.
-Pose your questions to the independent expert, see Appendix A for contact information.
-Read the information at www.rijksoverheid.nl/mensenonderzoek

**1. General information**

This study has been designed by the Department of Obstetrics and Gynecology of the Amsterdam University Medical Center, location AMC. The study will be conducted by gynecologists, clinicians or midwives. A total of 194 pregnant women with a short cervix and 44 women with dilatation of the cervix are required for this study. This study is approved by the Medical Ethics Committee (METC) Academic Medical Centre.

**2. Purpose of the study**

The purpose of this study is to investigate the effectiveness of a vaginal cerclage in women with a twin pregnancy and a midpregnancy short cervix compared to standard treatment (no cerclage) in the prevention of extreme preterm birth < 28 weeks of gestational age. The effectiveness of a vaginal cerclage will be compared with standard care according to the (inter)national guidelines, no cerclage.

**3. Background of the study**

Twin pregnancies have a high risk of extreme preterm birth (PTB) at less than 28 weeks of gestation which is associated with increased risk of neonatal morbidity and mortality. In the Netherlands, per year 250 women with a twin pregnancy deliver at < 28 weeks, resulting in 157 perinatal deaths. One of the risk factors for extreme preterm birth is a short cervix. If a short cervix is ≤ 25mm at the 20-week ultrasound, there is a high risk of an extremely premature birth. A possible effective surgical method to reduce extreme PTB in twin pregnancies with a short cervix or asymptomatic dilatation at midpregnancy is a minor operative procedure, the placement of a vaginal cerclage

**4. What does participation mean?**

*How long will the study last?*
Are you participating in the study? Then the study will run until three months after you gave birth. A few years after the study is finished, we may want to contact you to inquire through a questionnaire how you and your child are doing. At that time, you can decide whether you wish to answer our questions. No additional diagnostic tests are performed on you or your baby.

*Are you suitable for participation in the study?*
Women above the age of 16 years with a twin pregnancy and a short cervix or cervical dilatation below the 24$^{th}$ week of pregnancy can participate.  It may happen that you are healthy, but that you are not suitable to participate. The researcher will tell you more about this.

*The treatment*
If you consent to participate in the study you will be randomly assigned to either the group receiving standard care (no cerclage) or a cerclage. A vaginal cerclage is a minor surgical procedure in which an unabsorbable suture is placed around/through the cervix to close the cervical canal and to increase its firmness, in order to reduce cervical insufficiency and this pathway to preterm birth. The chance to end up in either one of the groups is equal.

*Examinations and measurements*
During the standard 20-week ultrasound (SEO), the cervix is measured in your own hospital. If the cervix is short (<25mm), you will be referred to a hospital that participates in this study. The participating hospital can decide to measure the cervical length again and if indicated perform physical examination to detect possible cervical dilatation. If you have drawn for vaginal cerclage group, the cerclage will be placed under anesthesia or epidural. You will be admitted to the hospital for one day.

During the study,  data about yourself, the pregnancy, the delivery and your children will be collected. For example, data such as your date of birth, ethnicity and level of education. Collecting data on ethnicity is important because people of different ethnicities may have different cervical lengths and may also respond differently to treatment.

Furthermore, we ask you to complete three online questionnaires: at the start of the treatment, after discharge from the hospital after the delivery and three months after delivery. Completing the questionnaires will take approximately 30 minutes. The questionnaires are completed online on a safe and private server. We ask your permission for using your email address to send you the questionnaires.

*Follow-up: What is different from regular care?*
If you have drawn for standard care, the pregnancy will be followed up by your own gynecologist according to the usual care. If you have drawn for a cerclage, the pregnancy will also be followed up by your own gynecologist with the usual check-ups after placement. . In addition to the treatment, no additional checks are necessary and no additional examinations will be performed. The cerclage will be removed at 36-37 weeks of pregnancy or sooner if labor starts before then.



van Gils L, *et al. BMJ Open* 2024; 14:e081561. doi: 10.1136/bmjopen-2023-081561

**5. What do we expect from you?**

We strive for the research to proceed as planned. Therefore, we will make the following agreements with you:

- You will come to every control visit
- You contact the investigator if:
    - You are hospitalized
    - You suddenly start having problems with your health
    - You no longer want to participate in the study
    - Your phone number, address or email address has changed

**6. What side effects, adverse reactions or discomforts may you experience?**

A vaginal cerclage is a minor and safe surgical procedure commonly performed in singleton pregnancies with a short cervix or dilatation and a previous preterm birth in all the participating centers, thus there is experience in the participating hospitals. Potential complications of a cerclage are infection, premature rupture of membranes, cervical laceration or bleeding and anesthesia-related complications, occurring in approximately 0.3-2.5 %. In addition, placing a cerclage is a surgical procedure and there are the small general risks of surgery and anesthesia or an epidural.

**7. Possible advantages and disadvantages**

It is important to weigh the pros and cons before you decide to participate. It is currently unclear whether a vaginal cerclage reduces preterm birth rates in women with a twin pregnancy and a short cervix. We cannot guarantee you will benefit from participating in this study. The study may however be useful for other pregnant women in the future and help in the search for better treatment of premature birth in twin pregnancies

*If you do not wish to participate or wish to stop your participation*

Your participation in this study is completely voluntary. If you do not wish to participate, you will be treated according to local protocol (no cerclage). If you decide not to participate, we would like to ask for your consent to collect, use and store data from your pregnancy and childbirth in order to gain insight into the treatment of preterm birth in twin pregnancies. If you decide that we are allowed to collect your data from your pregnancy and childbirth, we would like to ask you to sign the consent statement in Appendix D.

**8. The end of the study**

If there is new information about the study that is of interest to you, the investigator will inform you. You will then be asked whether you want to continue your participation.

Your participation in the study stops when:

- Three months after you gave birth
- You decide to withdraw from the study. You can decide to stop at any time during the study and you can inform the investigator about this. You do not have to explain why you want to discontinue participation.
- If you physician decides it's better for you to stop-If the investigator, the government or the judging medical ethical committee decide to stop the study

*What happens when you discontinue participation?*

The researchers will use your data that is collected until the moment you decide to discontinue the study. If you are assigned to the cerclage group, your physician will discuss your wishes regarding the cerclage. Overall, the study is concluded once all the participants have completed the study.



van Gils L, *et al. BMJ Open* 2024; 14:e081561. doi: 10.1136/bmjopen-2023-081561

**9. Usage and storage of your data**

Are you participating in the study? If so, then you also give us permission to use and store your personal data.

What personal data do we keep?

- Your and your babies' names
- Your and your babies' genders
- Your address
- Your and your babies date of birth
- Your ethnicity
- Your level of education
- Information about your and your babies' health
- Data about your pregnancy and delivery
- (Medical) data that we collect during the study

*Why do we collect, use and store your data?*

We collect, use and store your and your children's data to answer the research questions and to be able to publish the results.

*How do we protect your privacy?*

To protect your privacy, your data is encrypted. All your data can only be traced back to you with a key of the code. The key to the code remains securely stored in the local research facility. When processing your data, we only use this code. The data cannot be traced back to you in reports and publications about the study.

*Who will have access to my data?*

Some people can access all the data at the study location, including the data without a code. This is necessary to check whether the study is being conducted in a good and reliable manner. Persons who have access to your data for review are:

-Members of the committee that monitors the safety of the study

- A monitor hired by the sponsor(Amsterdam UMC, location AMC)

-National and international supervisory authorities. They keep your information secret. We ask you to give permission for this access. The Health and Youth Care Inspectorate (IGJ) can access your data without your permission.

*How long will your data be stored?*

We keep your records stored in the hospital and at the sponsor for 15 years.

*Can we use your data for other research?*

After this study, your data may also be important for other scientific research in the field of prevention of preterm birth in twin pregnancies. To this end, your data will be stored in the hospital for 15 years. You can indicate on the consent form whether you agree with this. If you do not agree to this, you can still participate in the current study. You will receive the same care as any other pregnant women in your situation.

*Can you withdraw your consent for the use of your personal data?*



van Gils L, *et al. BMJ Open* 2024; 14:e081561. doi: 10.1136/bmjopen-2023-081561

You can withdraw your consent to the use of your personal data and that of your child at any time. This applies to the use of your data for this current study and also to storage and use for future research. Please note: when you withdraw your consent, the data collected up to the time you withdraw your consent may still be used in the research.

*More information about your rights when processing data*
-Would you like to know more about your rights in the processing of personal data? Then please visit the following website: www.autoriteitpersoonsgegevens.nl.
-Do you have questions about your rights? Or do you have a complaint about the processing of your personal data? Please contact the person responsible for processing your personal data. For this study: [Name of institute] See Appendix A for the contact details and website.
-If you have questions or complaints about the processing of your personal data, we advise you to first contact the research team. You can also contact the Data Protection Officer of [the instite],Amsterdam UMC, or the Dutch Data Protection Authority.

*Where can you find additional information on the study?*
The following website provides more information about the study: https://www.zorgevaluatienederland.nl. You can find the study by searching for TWIN Cerclage.

**10. Do you have an insurance during the study**
Insurance has been provided for everyone who participates in this study. The insurance pays for damage caused by the research. But not for all damages. Appendix B contains more information about the insurance and the exceptions. It also tells you who you can report damage to.

**11. We will inform your general practitioner and/or treating specialist.**
The researcher will send your treating specialist a letter or e-mail to let him/her know that you are participating in this study. We will also inform your general practitioner if you get a cerclage.

**12. You have any questions?**
You can ask questions about the study to the investigators of this study. Would you like advice from an independent expert? If so, contact details can be found in appendix A. If you have any complaints about the study, you can discuss this with the investigator or your treating specialist. If you prefer not to do so, you may contact the complaints' officer/committee at your hospital. All the relevant details can be found in Appendix A.

**13.How do you give consent for the study?**
You should first think carefully about participating in this study. Afterwards, you tell the researcher whether you understand the information and whether you want to participate or not. If you would like to participate, please complete the consent form that you can find with this information sheet. You and the researcher will both receive a signed version of this consent form.

Thank you for your attention.

**14.Appendices to this information**
A.Contact details [each participating hospital needs to change this section]
B.Insurance information

C.Informed Consent Form

D.Informed consent form for the collection, use and store of data from your pregnancy, your delivery and details of your children



van Gils L, *et al. BMJ Open* 2024; 14:e081561. doi: 10.1136/bmjopen-2023-081561

## Appendix A: Contact details for (name participating center)

(Researcher): [for principal investigator of centre: name, contact details and availability]

<if applicable>
[Research nurse/research doctor/nurse specialist]

Independent expert: If you have any doubts about participation, you can consult an independent expert who is not involved in the study herself, but knows a lot about this study. Also if you have questions before or during the study that you would rather not ask your doctor, you can contact the independent expert: Dr. A. Timmermans. She can be reached by telephone via the office of the women's clinic: 020-5663754 on working days during office hours

Complaints:
[service or person with contact details and accessibility]

The Data Protection Officer::
Name of institute: ……………………….
Data protection officier from the sponsor: privacy@amsterdamumc.nl.

For additional information on your rights with respect to the use of your personal data:
Name of institute: local PI
On behalf of the sponsor: Prof. dr. M.A. Oudijk twinc@amsterdamumc.nl

## Appendix B: Insurance information

Insurance has been taken out by the AMC for everyone participating in this study. The insurance covers damage due to participation in the study. This applies to damage manifesting during the study or within 4  years of the end of your participation in the study. You must notify the insurance company about the damage within those 4  years.

In the event of damage please contact the insurance company [or claims adjustor] directly.

The insurance company for the study is:
Name: Centramed B.A.
Address: Postbus 7374
2701 AJ Zoetermeer
Telephone number: 070 301 70 70
E-mail address: info@centramed.nl
Insurance number: 624.528.303

In case of damage, the subject is requested to contact Prof. Dr. M.A. Oudijk, 020-5663754.

The insurance offers a cover of €650,000 per study subject and €5,000,000 for the entire study. For all studies from the same sponsor, the maximum amount of coverage is. €7,500,000 annually.

The insurance policy does **not** cover the following damage:
-Damage as a result of a risk that you were informed about in the written information. This does not apply if the risk occurs in a more severe form than envisaged, or if the risk was very unlikely to occur;
-Damage to your health that would also have occurred if you had not participated in the study;
-Damage resulting from not or not entirely following directions or instructions;
-Damage to descendants as a result of a negative effect of the study on you or your descendants (unless the study specifically involves an existing pregnancy or birth, or embryos for the purpose of creating a pregnancy);
-Damage as a result of an existing treatment method or of research into existing methods of treatment.
- These provisions are set out in the 'Besluit verplichte verzekering bij medisch-wetenschappelijk onderzoek met mensen 2015''. This decision can be found in the government's Wettenbank (https://wetten.overheid.nl).



van Gils L, *et al. BMJ Open* 2024; 14:e081561. doi: 10.1136/bmjopen-2023-081561

## Appendix C: Informed consent form TWIN Cerclage studie

Belonging to TWIN Cerclage: The effectiveness of Cerclage for the reduction of extreme preterm birth and perinatal mortality in Twin pregnancies with a short cervix.

- I was able to read the information letter. I was also able to ask questions. My questions have been answered to my satisfaction. I had enough time to decide whether to participate.
- I know that participation is voluntary. I know that I may decide at any time not to participate after all or to withdraw from the study. I do not need to give a reason for this.
- I give the researcher permission to let my treating specialist know that I am participating in this study and your general practitioner if you have are classified to the cerclage group.
- I give consent that the researcher can ask additional information from my midwife, gynecologist general practitioner or specialist about my pregnancy, health and medical history.
- I consent to the collection and use of my data, including ethnicity and education level, and my child (ren)'s data in the manner and for the purposes set out in the information letter.
- I know that some people may have access to all my data to verify the study. These people are listed in this information sheet. I consent to the inspection by them.
- I give permission to keep my data and those of my child (ren) for 15 years after the end of this research.
- I give permission to forward my contact details (address and telephone number) to the research team at Amsterdam UMC so that they can approach me for possible follow-up research and sending the questionnaires

Would you please fill out yes or no in the following table?

| | | |
|---|---|---|
| I give permission to keep my data to use it for other research, as stated in the information letter. | Yes ☐ | No☐ |
| I give permission to be asked if I want to participate in a follow-up study after this study. | Yes ☐ | No ☐ |

- I want to take part in this study.

My name is (subject): ..............................................
My e-mail address:
My telephone number:

Signature: ...........................

Date: __/__/__

I declare that I have fully informed this subject about this study. If information comes to light during the course of the study that could affect the study subject's consent, I will inform him/her of this in a timely fashion. The study subject will receive the full information sheet, together with a signed copy of the consent form.

Investigator name (or their representative): ........................
Signature:

Date: __/__/__

## Appendix D. Statement of consent for the collection, use and storage of data about the pregnancy, childbirth and data of your children

If you decide not to participate in this study, we would like to ask for your consent to the collection, use and retention of data from your pregnancy and childbirth in order to gain a better understanding of threatened preterm birth in twin pregnancies. If you decide that we are allowed to collect your data from your pregnancy and childbirth, we would like to ask you to sign the consent statement.

- I was also able to ask questions. My questions have been answered to my satisfaction. I had enough time to decide whether to participate.
- I have the right to withdraw my consent at any time without having to give a reason.
- I give the researcher permission to request information about my pregnancy, delivery and children from my GP/specialist(s).
- I give permission to keep my data and those of my children for a period of 15 years after the end of the research.
- I give permission for the collection and use of my data, pregnancy and delivery and of my children for answering the research question in this study.
- I give permission to forward my contact details (address and telephone number) to the research team at Amsterdam UMC, so that they can approach me for possible follow-up research and sending the questionnaires.

| | | |
|---|---|---|
| I give permission to be asked if I want to participate in a follow-up study after this study. | Yes ☐ | No ☐ |

-

My name is (subject): ...............................................
E-mail address:
Telephone number:

Signature: ..........................

Date: __/__/__

I declare that I have fully informed this subject about this study. If information comes to light during the course of the study that could affect the study subject's consent, I will inform him/her of this in a timely fashion.



van Gils L, *et al. BMJ Open* 2024; 14:e081561. doi: 10.1136/bmjopen-2023-081561
