# Supplementary material for: Study protocol for two randomised controlled trials evaluating the effects of Cerclage in the reduction of extreme preterm birth and perinatal mortality in twin pregnancies with a short cervix or dilatation: the TWIN Cerclage studies
Source: BMJ Open. 2024 May 10;14(5):e081561. doi: 10.1136/bmjopen-2023-081561 (PMC11097875; doi:10.1136/bmjopen-2023-081561)
Supplement: Author's manuscript [file bmjopen-2023-081561.draft_revisions.pdf]

BMJ Open is committed to open peer review. As part of this commitment we make the peer review history of every article we publish publicly available.

When an article is published we post the peer reviewers' comments and the authors' responses online. We also post the versions of the paper that were used during peer review. These are the versions that the peer review comments apply to.

The versions of the paper that follow are the versions that were submitted during the peer review process. They are not the versions of record or the final published versions. They should not be cited or distributed as the published version of this manuscript.

BMJ Open is an open access journal and the full, final, typeset and author-corrected version of record of the manuscript is available on our site with no access controls, subscription charges or pay-per-view fees (http://bmjopen.bmj.com).

If you have any questions on BMJ Open's open peer review process please email info.bmjopen@bmj.com

BMJ Open

Study protocol for two randomized controlled trials evaluating the effect of cerclage in the reduction of extreme preterm birth and perinatal mortality in twin pregnancies with a short cervix or dilatation: the TWIN Cerclage studies.

Journal:	*BMJ Open*
Manuscript ID	bmjopen-2023-081561
Article Type:	Protocol
Date Submitted by the Author:	01-Nov-2023
Complete List of Authors:	van Gils, Lissa; Amsterdam UMC Locatie AMC, Department of Obstetrics and Gynaecology Amsterdam<br>de Boer, Marjon; Amsterdam UMC Locatie VUmc, Department of Obstetrics and Gynaecology<br>Bosmans, Judith; Amsterdam UMC Location AMC, Department of Health Sciences<br>Duijnhoven, Ruben; Amsterdam UMC Location AMC, Obstetrics and Gynaecology<br>Schoenmakers, Sam ; Erasmus Medical Center, Department of Obstetrics and Gynaecology<br>Derks, J. ; University Medical Centre Utrecht, Obstetrics<br>Prins, Jelmer; University Medical Center Groningen, Department of Obstetrics and Gynaecology<br>Al-Nasiry, Salwan; Maastricht UMC+, Department of Obstetrics and Gynaecology<br>Lutke Holzik, Margo; Leiden Universitair Medisch Centrum, Department of Obstetrics and Gynecology<br>Lopriore, Enrico; Leiden University Medical Center, Department of neonatology and fetal medicine<br>van Drongelen, J. ; Radboud Universiteit, Department of Obstetrics and Gynecology<br>Knol, H. ; Isala Zwolle, Department of Obstetrics and Gynaecology<br>van Laar, J.; Máxima Medical Center, Department of Obstetrics & Gynaecology<br>Jacquemyn, Y.; University Hospital Antwerp<br>van Holsbeke, Caroline; AZ Sint-Lucas Brugge<br>Dehaene, Isabelle; University Hospital Ghent,<br>Lewi, Liesbeth; KU Leuven University Hospitals Leuven, Fetal Medicine Unit<br>van der Merwe, Hannes; KU Leuven University Hospitals Leuven<br>Gyselaers, Wilfried; Hospital Oost-Limburg, Department of Obstetrics and Gynecology<br>Obermann-Borst, Sylvia; Care4Neo, Neonatal patient and parent advocacy organization.<br>Holthuis, Mayella; Patiëntenorganisaties Extreme Vroeggeboorte<br>Mol, Ben; Monash Medical School, OB/GYN<br>Pajkrt, Eva; Amsterdam UMC Location AMC, Obstetrics and Gynaecology<br>Oudijk, Martijn; AMC, Obstetrics

Keywords:	Fetal medicine < OBSTETRICS, Maternal medicine < OBSTETRICS, OBSTETRICS

SCHOLARONE™
Manuscripts

Study protocol for two randomized controlled trials evaluating the effect of cerclage in the reduction of extreme preterm birth and perinatal mortality in twin pregnancies with a short cervix or dilatation: the TWIN Cerclage studies.

Authors

Drs. Lissa van Gils [1]

Dr. Marjon A. de Boer [1]

Prof. Dr. J. Bosmans [2]

Dr. Ruben Duijnhoven[1]

Dr. Sam Schoenmakers [3]

Dr. Jan B. Derks [4]

Dr. Jelmer R. Prins [5]

Dr. Salwan Al-Nasiry [6]

Dr. Margo Lutke Holzik [7]

Dr. Enrico Lopriore [8]

Dr. Joris van Drongelen [9]

Dr. Marieke H. Knol [10]

Dr. Judith O.E.H. van Laar [11]

Prof. Dr. Yves Jacquemyn [12]

Dr. Caroline van Holsbeke [13]

Dr. Isabelle Dehaene [14]

Prof. Dr. Liesbeth Lewi [15]

Dr. Hannes van der Merwe [15]

Dr. Wilfried Gyselaers [16]

Dr. Sylvia A. Obermann-Borst [17]

Mayella Holthuis [18]

Prof. Dr. Ben W. Mol [19]

Prof. Dr. Eva Pajkrt [1]

Prof Dr. Martijn A. Oudijk [1]

For peer review only

Affiliations

[1] Department of Obstetrics and Gynaecology, Amsterdam University Medical Center, De Boelelaan 1118, 1081 HZ Amsterdam, The Netherlands

[2] Department of Health Sciences and the EMGO Institute for Health and Care Research, Faculty of Earth and Life Sciences, Vrije Universiteit Amsterdam, De Boelelaan 1085, 1081 HV Amsterdam, The Netherlands

[3] Department of Obstetrics and Gynaecology, Erasmus Medical Center, 's- Gravendijkwal 230, 3015 CE Rotterdam, The Netherlands

[4] Department of Obstetrics and Gynaecology, Birth Centre Wilhelmina Children Hospital, University Medical Center Utrecht, Heidelberglaan 100, 3584 CX Utrecht, The Netherlands

[5] Department of Obstetrics and Gynaecology, University Medical Center Groningen, Hanzeplein 1, 9713 GZ Groningen, The Netherlands

[6] Department of Obstetrics and Gynaecology, Maastricht University Medical Center, P. Debyelaan 25, 6229 HX Maastricht, The Netherlands

[7] Department of Obstetrics and Gynaecology, Leiden University Medical Center, Albinusdreef 2, 2333 ZA Leiden, The Netherlands

[8] Department of neonatology and fetal medicine, Leiden University Medical Center, Albinusdreef 2, 2333 ZA Leiden, The Netherlands

[9] Radboud University Medical Center, Department of Obstetrics and Gynaecology, Geert Grooteplein-Zuid 10, 6525 GA Nijmegen, The Netherlands

[10] Department of Obstetrics and Gynaecology, Isala Zwolle, Zwolle, Netherlands

[11] Department of Obstetrics and Gynaecology, Maxima Medical Center in Veldhoven, De Run 4600, 5504 DB Veldhoven, The Netherlands

[12] Department of Obstetrics and Gynaecology, University Hospital, Antwerpen, Belgium

[13] Department of Obstetrics and Gynaecology, AZ Sint-Lucas, Brugge, West-Vlaanderen, Belgium

[14] Department of Obstetrics and Gynaecology, University Hospital Ghent, Corneel Heymanslaan 10, Gent, BE 9000

[15] Leuven University Hospitals Leuven, Fetal Medicine Unit Leuven, Flanders, BE

[16] Hospital Oost-Limburg, Department of Obstetrics and Gynaecology, Genk, BE

[17] Care4Neo, Neonatal patient and parent advocacy organization.Rotterdam, NL

[18] Patiëntenorganisaties Extreme Vroeggeboorte, Amsterdam, NL

[19] Department of Obstetrics and Gynaecology, Robinson Research Institute, University of Adelaide, 72 King William St, North Adelaide, SA 5006 Australia

For peer review only

Corresponding author: L. van Gils, Department of Obstetrics and Gynaecology, Amsterdam University Medical Center, De Boelelaan 1118, 1081 HZ Amsterdam, The Netherlands. (+31)642811240. L.vangils1@amsterdamumc.nl

Authors contribution: All authors were involved in conception and design of the study. LG and MO drafted the manuscript. All authors reviewed and edited the manuscript. All authors mentioned in the manuscript are member of the TWIN Cerclage study group. They participated in the design of the study and are local investigators in the participating centres.

Funding: This study is funded by ZonMw (The Netherlands organisation for health research and development), grant number 10390012110017.

Competing interests: None.

For peer review only

Abstract

Introduction; Twin pregnancies have a high risk on extreme preterm birth (PTB) at less than 28 weeks of gestation which is associated with increased risk of neonatal morbidity and mortality. Currently there is a lack of effective treatments in women with a twin pregnancy and a short cervix or cervical dilatation. A possible effective surgical method to reduce extreme PTB in twin pregnancies with an asymptomatic short cervix or dilatation at midpregnancy is the placement of a vaginal cerclage.

Methods and analysis; We designed two international multicentre randomized trials. Women above the age of 16 years with a twin pregnancy < 24 weeks of gestation and an asymptomatic short cervix ≤ 25mm or cervical dilatation, will be randomly allocated to vaginal cerclage or standard treatment according to the current Dutch NVOG guideline (no cerclage). The primary outcome measure is PTB <28 weeks. Analyses will be by intention to treat. The first trial is to demonstrate a risk reduction from 25% to 10% in the short cervix group, for which 194 patients need to be recruited. The second trial is to demonstrate a risk reduction from 80% to 35% in the dilatation group and will need to recruit 44 women. A cost-effectiveness analysis will be performed from a societal perspective.

Ethics and dissemination: This study has been approved by the Research Ethics Committees (REC) in the Netherlands and Belgium. The results will be presented at conferences and published in a peer-reviewed journal. Participants will be informed about the results.

Trial registration number: The trials are registered at www.clinicaltrials.gov with ID NCT05968794.

Strengths of this study

- These are the largest randomized trials comparing the effect of cerclage on PTB in women with a twin pregnancy and a short cervix or dilatation.
- Outcomes assessed will be preterm birth at different gestational age thresholds and adverse maternal and neonatal outcomes.
- Intention to perform long term follow-up of children thus future parents can be counselled on the long-term effects of this intervention.

Limitation of this study

- The recruitment of patients could be a challenge as the incidence of women with a twin pregnancy and a short cervix ≤ 25 mm or dilatation at midpregnancy is low.

Key-words: Preterm birth, twins, cerclage, randomized controlled trial

For peer review only

Introduction

Twin pregnancies have a high risk on extreme preterm birth (PTB) at less than 28 weeks of gestation which is associated with increased risk of neonatal morbidity and mortality.(1) Worldwide, twins represent 0.5–4.0% of all births, with rates varying between ethnic groups and countries.(2) Compared with singletons, twins are at increased risk for adverse perinatal outcomes.(3) In the Netherlands, 250 (5,2%) women with a twin pregnancy deliver at < 28 weeks per year, resulting in 157 perinatal deaths and 343 surviving neonates of whom a large proportion suffer from long term neurodevelopmental problems associated with extreme preterm birth.(4) An asymptomatic short cervix (≤ 25 mm) or dilatation at midpregnancy in twin pregnancies is associated with high rates of extreme PTB and proven to be the best predictor for extreme PTB.(5, 6)

Effective interventions for twin pregnancies with short cervix are limited. The EPPPIC individual participant data (IPD) meta-analysis on progesterone treatment in multiple pregnancies with a short cervix showed no reduction of PTB, however a recent IPD meta-analysis did show a possible effect of progesterone.(7, 8) The use of a vaginal pessary in women with a twin pregnancy and a short cervix does not seem to result in a reduction of PTB. (9)

A possible surgical method to reduce extreme PTB in twin pregnancies with an asymptomatic short cervix or dilatation at midpregnancy is a vaginal cerclage. An IPD meta-analysis from 2015 assessed its impact in 49 women with a twin pregnancy and a short cervix (< 25 mm) across three randomized controlled trials.(10) No significant differences were present between the groups on outcomes such as PTB or perinatal death. However, a more recent systematic review with a total of 471 women also included six cohort studies that focused on the outcome of PTB at different cut-offs.(11) The pooled results of these cohort studies showed a significant beneficial effect of a cerclage on PTB <32 weeks (Relative risk (RR) 0.68; 95% confidence interval (CI) 0.51-0.92) and <34 weeks (RR 0.73; 95% CI 0.59-0.90). In addition, a cerclage significantly prolonged pregnancy (mean 2.53 weeks (95% CI 1.25-3.81)). No significant difference was seen in the number of PTBs <24 and 28 weeks or perinatal mortality, although the trend was in favour of the cerclage group.

Furthermore, a subgroup analysis by cervical length was carried out, distinguishing between a cervical length between 16 and 24mm and ≤15mm.(11) For the group with a cervical length <15mm, cerclage placement was associated with less PTB <37 weeks (RR 0.86; 95% CI 0.74-0.99), <34 weeks (RR 0.57; 95% CI 0.43-0.75) and <32 weeks (RR 0.61; 95% CI 0.41-0.90) compared to the control group. Cerclage also resulted in a significant pregnancy prolongation by 3.89 weeks (95% CI 2.19-5.59). In the group of women with a cervical length of 16-24mm, no significant differences were seen in the outcomes.

In women with a twin pregnancy and asymptomatic cervical dilatation, the most recent systematic review was published in August 2023 and included 1 RCT and 5 retrospective cohort studies.(12) These studies showed that a vaginal cerclage may result in a reduction of PTB at less than 24 weeks (RR 0.32; 95% CI 0.21-0.48), 28 weeks (RR 0.47; 95% CI 0.36-0.62), 32 weeks (RR 0.59; 95% CI 0.50–0.70) and 34 weeks (RR 0.68; 95% CI 0,57–0,80). In addition, women with a cerclage had a reduced risk of perinatal death (RR 0.30; 95% CI 0.16-0.55), 5 minute Apgar score < 7 (RR 0.49; 95% CI 0.27-0.90), birthweight <1500 grams (RR 0.41; 95% CI 0.31-0.55) and admission to the neonatal intensive care unit (NICU) (RR 0.67; 95% CI 0.52-0.88).

For peer review only

In conclusion, there is a lack of well-designed RCT's on the effect of vaginal cerclage, on PTB and neonatal outcomes in women with an twin pregnancy with a short cervix or asymptomatic dilatation at midpregnancy. Long term follow-up of children is lacking in general, thus future parents cannot be counselled on the long term effects of this intervention. The possible effect of a cerclage in cohort studies justifies a well-designed large multicentre randomized trial. We therefore will perform the TWIN Cerclage study.

Objectives: To assess the effectiveness of a vaginal cerclage in women pregnant with a twin pregnancy with a midpregnancy asymptomatic short cervix or dilatation in the prevention of extreme PTB and perinatal mortality compared to standard treatment (no cerclage).

Methods and analysis

Design and setting

We will conduct two international multicentre randomized controlled trials within the Dutch Consortium for Healthcare Evaluation and Research in Obstetrics and Gynaecology and participating hospitals in Belgium.

Participants / eligibility criteria

RCT I: Women (> 16 years of age) with a twin pregnancy with a short cervix ≤ 25 mm below 24 weeks of pregnancy are eligible for this trial.

RCT II: Women (> 16 years of age) with a twin pregnancy with dilatation on transvaginal ultrasound or physical examination below 24 weeks of pregnancy are eligible for this trial.

Women who meet any of the following criteria will be excluded from participation in these trials:

- Women with a mono-amniotic twin pregnancy
- Women with twin pregnancy in which one or both children are diagnosed with a major structural, congenital or chromosomal abnormality (at time of inclusion) that is likely to influence the composite adverse neonatal outcome.
- Women with signs of clinical intra-uterine infection, defined by the presence of fever ≥ 38 degrees Celsius.
- Women with overt symptoms of preterm labour at time of measurement of short cervix or dilatation (regular contractions, PPROM, recurrent blood loss).
- Women with a placenta previa, defined as a placenta position covering the internal ostium of the cervix.
- Women who do not master the Dutch or English language and are therefore not able to give written consent

Treatment

The investigated intervention is a vaginal cerclage. Potential complications of a cerclage are infection, premature rupture of membranes, cervical laceration or bleeding and anaesthesia-related complications, occurring in approximately 0.3-2.5 %.(13)

The comparator will be standard treatment according to the current guideline in the Netherlands from 2018, which is to not perform or offer an intervention such as vaginal cerclage.(14) This is in line with the current standard of care in most of the participating hospitals in Belgium.

For peer review only

Study procedures

Measurement of cervical length in twin pregnancies will be performed as part of the standard 20 week ultrasound scan in all hospitals in the Netherlands and participating centres in Belgium. In case of a short cervical length or dilatation patients will be seen at the nearest participating centre, where women will be counselled for participation in the study. Inclusion is possible below 24 weeks of gestation, and if randomized to the surgical treatment, the cerclage needs to be placed before 24^{+0} weeks of gestation.

After women give written consent to participate in the trials, patients will be randomized for two treatment options: 1. surgical treatment with cerclage and additional standard care according to Dutch guidelines or 2. Standard care according to Dutch guidelines (no cerclage). Randomization will be done using a web-based interface for each of the two trials. For the trial recruiting women with dilatation, random permuted blocks will be used of sizes 2 and 4 to minimize the risk of disbalance. Participants and investigators will not be blinded for the intervention.

In addition, participants are treated according to the local protocol in the participating clinics i.e. the use of progesterone during pregnancy and other interventions such as tocolysis and corticosteroids in case of a threatened PTB. Follow up of the pregnancy and delivery will be in the referral centre until delivery. The cerclage will be removed according to protocol between 36-37 weeks of gestation, or in case of labour, whatever comes first. Delivery will take place by either spontaneous onset of labour, or induction of labour or elective caesarean section according to national guidelines for twin pregnancies.

The referral hospitals will be contacted for data collection of outcomes as mentioned above by the studies' research midwifes or nurses. Primary and secondary outcome measures will be obtained from clinical patient data. Outcomes such as PTB will be assessed at birth, additional outcomes will be assessed at the time of discharge of the child from hospital to home address and at 3 months after discharge.

Outcome measures

Outcome parameters are in line with the core outcome set for studies on prevention of PTB defined by members of GONet and the Core Outcomes in Women's health (CROWN) initiative.(15) The primary outcome measure is PTB <28 weeks. The main secondary outcome will be a composite for adverse neonatal outcome (including bronchopulmonary dysplasia, periventricular leucomalacia > grade 1, intraventricular haemorrhage > grade 2, necrotizing enterocolitis > stage 2, retinopathy of prematurity > stage 2, proven sepsis and perinatal death). All components of the composite outcome will also be assessed separately.

Further secondary outcomes will be PTB (indicated and spontaneous) at less than 24, 32, 34 and 37 weeks, premature rupture of membranes (PPROM), gestational age at delivery, number of days on ventilation support and number of days in NICU. Other maternal outcomes will be mortality, infection with need for antibiotics or sepsis and need to remove cerclage in operation room <36 weeks.

Parents will be asked consent to approach the parents for long-term follow-up of the children at 2 and 4 years of age. Maternal quality of life will be assessed at inclusion, discharge after birth and 3 months postpartum using the five level version of the EuroQol (EQ-5D-5L).(16) Costs will be

For peer review only

For peer review only - http://bmjopen.bmj.com/site/about/guidelines.xhtml

measured from a societal perspective using web-based questionnaires based on the iMCQ and iPCQ (Medical and Productivity Cost Questionnaires) at discharge after birth and at 3 months after discharge for the mother and the neonate.

In addition, pregnancy outcome data will also be collected for women opting not to participate in the trials but giving informed consent to collect data on pregnancy outcome from hospital records. Women give permission for this on a voluntary basis.

Withdrawal of subjects

Subjects can leave the study at any time for any reason if they wish to do so, without any consequences. Patients who withdraw from the study will remain in their treatment group for the intent-to-treat analysis, their data will be used for analysis. The investigator can decide to withdraw a subject from the study for urgent medical reasons.

Monitoring and safety

An independent data safety monitoring board (DSMB) will monitor the trials for safety. The DSMB consists of several members with expertise in the relevant fields of obstetrics, neonatology, epidemiology and statistics. Serious adverse events (SAEs) will be collected from the first study-related procedure until 3 months after delivery. SAE's must be reported by e-mail to the study project team within 24 hours after the event was reported to the investigator.

An interim analysis will be conducted for safety (i.e. interim safety review) at least after the outcomes for the first 50 and 100 patients have become available in RCT I (short cervix), or the first 22 patients (half the planned sample size) in RCT II (dilatation group).

Sample size

To demonstrate a risk reduction from 25% to 10% in the short cervix trial, 97 patients need to be recruited per arm (194 in total). This sample size was calculated using a Z-test with unpooled variance, a two-sided alpha of 0.05 and a target power of 80%.

In addition, for a risk reduction from 80% to 35% in the dilatation trial, 22 patients need to be recruited per arm (44 in total). The sample size for this trial was calculated using Fisher's exact test, with a two-sided alpha of 0.05 and a target power of 80%. Numbers are not corrected for loss of follow-up, as it is highly unlikely to have loss to follow-up for short-term outcomes in these high-risk pregnancies.

Statistical analysis

Data analysis will be performed according to the intention-to-treat principle. A supplementary per-protocol analysis is considered if any patients in the standard care arm would have received a cerclage (protocol deviation). Categorical variables will be expressed as a number with the percentage of the total allocation arm. Continuous variables will be presented as mean with standard deviation, as geometric mean with 95% confidence interval (CI) or as median with interquartile range, whenever appropriate.

The primary outcome will be evaluated using the intention-to-treat population. Estimand for both RCT I and RCT II is the relative risk for extreme PTB after cerclage, as compared to the control group not undergoing cerclage. Confidence interval will be a two-sided 95% interval, with statistical

For peer review only

inference based on the Chi-squared test for RCT I, and Fisher's exact test for RCT II. Heterogeneity of treatment effect is anticipated to be substantial for the two trials, and a pooled estimate will only be provided would heterogeneity be low. Heterogeneity will be assessed using a forest plot and tests for interaction in a generalized linear model. The two trials may be reported separately if not completed within a reasonable time from each another to allow timely communication of results.

Missing data for the primary outcome will be very low, given that the primary outcome is both an outcome recorded for routine care and there is a short follow-up time. If patients would deliver in other hospitals than the hospital they were recruited from, the data can still be obtained. Only patients who actively revoke consent cannot be analysed for the primary outcome.

The secondary outcome measures be approached similarly to the primary outcome measure. Dichotomous outcomes will be presented as relative risks and absolute with 95% confidence intervals and chi-squared tests or Fisher's exact test. Continuous secondary outcomes, differences between groups will be assessed with the student's t-test if the outcome is normally distributed and with a non-parametric Mann-Whitney U test if skewed. These outcomes will be presented as means with standard deviation, geometric means with 95% CI, or as median with interquartile range, whichever appropriate. A nominal level of 5% will be used for significance. From the perspective of type I error control the two trials are considered as independent tests.

Subgroup analyses

The following subgroup analyses are planned:

- Cervical length ≤ 15 mm and 16-25 mm
- Dichorionic and monochorionic pregnancies
- Multiparous women with a history of PTB and women without a history of PTB
- Nulliparous women and multiparous women with a term birth in their obstetric history

Economic evaluation

Both a cost-effectiveness analysis (CEA) and a cost-utility analysis (CUA) will be performed from a societal and healthcare perspective according to Dutch guidelines with a time horizon of 3 months after discharge. All statistical analyses will be done according to the intention-to treat principle. Missing cost and effect data will be imputed using multiple imputation according to the MICE algorithm. Rubin's rules will be used to pool the results from the different multiply imputed datasets. Linear regression analyses will be used to estimate cost and effect differences between intervention and control while adjusting for confounders if necessary. Incremental cost-effectiveness ratios (ICERs) will be calculated by dividing the difference in the mean total costs between the treatment groups by the difference in mean effects between the treatment groups. Bias-corrected and accelerated bootstrapping with 5000 replications will be used to estimate 95% confidence intervals around the cost differences and statistical uncertainty surrounding the ICERs. Uncertainty surrounding the ICERs will be graphically presented on cost-effectiveness planes. Cost-effectiveness acceptability curves will also be estimated showing the probability that the intervention is cost-effective in comparison with control for a range of different ceiling ratios thereby showing decision uncertainty.(17)

For peer review only

Budget impact analysis

A budget impact analysis (BIA) will be conducted from the perspective of health-care decision makers according to the Dutch guidelines and the recommendations from Sullivan et al using the Dutch BIA tool.(18) In the BIA, data from the effectiveness and cost-effectiveness analyses regarding the differences in costs and health outcomes will be combined with national prevalence and incidence data to extrapolate the findings to a time horizon of 5 years. The BIA will be conducted from the government perspective (Budget Kader Zorg) and the societal perspective. Actual NZA tariffs will be used to calculate costs. The budget analysis will differentiate between incidental and structural cost (savings), and will take into account budgetary consequences of changes within these cost components. Sensitivity analyses will be performed for subgroups of patients if applicable, providing budget information for relevant subgroups to decision makers. In addition, sensitivity analyses will address the impact of variations of the main assumptions and input parameters for the BIA.

Patient and public involvement

The Dutch consortium collaborates with three Dutch patient associations, Care4Neo (Neonatal patient and parent advocacy organization), the 'Vereniging voor Ouders van Meerlingen (NVOM)' (society of parents of multiples) and the patient organization for extreme PTB. They are project members of the TWINC Cerclage study and involved in the design of the study. A survey was performed during the design of the study among members of the closed Facebook group of both patient associations, to evaluate the interest in participation in the TWIN Cerclage study. The Dutch consortium has a website where it publishes all results of completed studies, and publishes the protocols of currently recruiting studies.

Ethics and dissemination

The Research Ethics Committee (REC) at the Amsterdam University Medical Centres approved this study. Additional regional approval was obtained for the remaining participating hospitals in The Netherlands. Protocol amendments will be communicated to relevant parties.

A manuscript with the results of the primary study will be published in a peer-reviewed journal and presented at conferences. A separate manuscript will be written on the cost-effectiveness analysis.

References

1. Schaaf JM, Mol BW, Abu-Hanna A, Ravelli AC. Trends in preterm birth: singleton and multiple pregnancies in the Netherlands, 2000-2007. BJOG. 2011;118(10):1196-204.
2. Deng C, Dai L, Yi L, Li X, Deng K, Mu Y, et al. Temporal trends in the birth rates and perinatal mortality of twins: A population-based study in China. PLoS One. 2019;14(1):e0209962.
3. Russo FM, Pozzi E, Pelizzoni F, Todyrenchuk L, Bernasconi DP, Cozzolino S, Vergani P. Stillbirths in singletons, dichorionic and monochorionic twins: a comparison of risks and causes. Eur J Obstet Gynecol Reprod Biol. 2013;170(1):131-6.
4. data P. Perinatale registratie Nederland. 2019.
5. Honest H, Bachmann LM, Coomarasamy A, Gupta JK, Kleijnen J, Khan KS. Accuracy of cervical transvaginal sonography in predicting preterm birth: a systematic review. Ultrasound Obstet Gynecol. 2003;22(3):305-22.
6. Lim AC, Hegeman MA, Huis In TVMA, Opmeer BC, Bruinse HW, Mol BW. Cervical length measurement for the prediction of preterm birth in multiple pregnancies: a systematic review and bivariate meta-analysis. Ultrasound Obstet Gynecol. 2011;38(1):10-7.

For peer review only

7. Group E. Evaluating Progestogens for Preventing Preterm birth International Collaborative (EPPPIC): meta-analysis of individual participant data from randomised controlled trials. Lancet. 2021;397(10280):1183-94.
8. Romero R, Conde-Agudelo A, Rehal A, Da Fonseca E, Brizot ML, Rode L, et al. Vaginal progesterone for the prevention of preterm birth and adverse perinatal outcomes in twin gestations with a short cervix: an updated individual patient data meta-analysis. Ultrasound Obstet Gynecol. 2022;59(2):263-6.
9. Hoffman M. Randomized trial of cervical pessary for short cervix in singleton pregnancies. American journal of obstetrics and gynecology. 2023;228(1):S5-S6.
10. Saccone G, Rust O, Althuisius S, Roman A, Berghella V. Cerclage for short cervix in twin pregnancies: systematic review and meta-analysis of randomized trials using individual patient-level data. Acta Obstet Gynecol Scand. 2015;94(4):352-8.
11. Li C, Shen J, Hua K. Cerclage for women with twin pregnancies: a systematic review and metaanalysis. Am J Obstet Gynecol. 2019;220(6):543-57 e1.
12. D'Antonio F, Eltaweel N, Prasad S, Flacco ME, Manzoli L, Khalil A. Cervical cerclage for prevention of preterm birth and adverse perinatal outcome in twin pregnancies with short cervical length or cervical dilatation: A systematic review and meta-analysis. PLoS medicine. 2023;20(8):e1004266.
13. van Dijk CE, Breuking SH, Jansen S, Limpens J, Kazemier BM, Pajkrt E. Perioperative complications of a transvaginal cervical cerclage in singleton pregnancies: a systematic review and meta-analysis. AMERICAN JOURNAL OF OBSTETRICS AND GYNECOLOGY. 2023;228(5):521-52+.
14. (NVOG) DSfOaGNVvOeG. Preventie-Vroeggeboorte-bij-Meerlingzwangerschappen-def-2018. 2018.
15. van 't Hooft J. A core outcome set for evaluation of interventions to prevent preterm birth: summary for CROWN. BJOG. 2016;123 Suppl 3:107.
16. M MV, K MV, S MAAE, de Wit GA, Prenger R, E AS. Dutch Tariff for the Five-Level Version of EQ-5D. Value Health. 2016;19(4):343-52.
17. Fenwick E, O'Brien BJ, Briggs A. Cost-effectiveness acceptability curves--facts, fallacies and frequently asked questions. Health Econ. 2004;13(5):405-15.
18. Sullivan SD, Mauskopf JA, Augustovski F, Jaime Caro J, Lee KM, Minchin M, et al. Budget impact analysis-principles of good practice: report of the ISPOR 2012 Budget Impact Analysis Good Practice II Task Force. Value Health. 2014;17(1):5-14.

For peer review only

BMJ Open

Study protocol for two randomized controlled trials evaluating the effect of cerclage in the reduction of extreme preterm birth and perinatal mortality in twin pregnancies with a short cervix or dilatation: the TWIN Cerclage studies.

Journal:	*BMJ Open*
Manuscript ID	bmjopen-2023-081561.R1
Article Type:	Protocol
Date Submitted by the Author:	15-Nov-2023

Complete List of Authors:	van Gils, Lissa; Amsterdam UMC Locatie AMC, Department of Obstetrics and Gynaecology Amsterdam<br>de Boer, Marjon; Amsterdam UMC Locatie VUmc, Department of Obstetrics and Gynaecology<br>Bosmans, Judith; Amsterdam UMC Location AMC, Department of Health Sciences<br>Duijnhoven, Ruben; Amsterdam UMC Location AMC, Obstetrics and Gynaecology<br>Schoenmakers, Sam ; Erasmus Medical Center, Department of Obstetrics and Gynaecology<br>Derks, J. ; University Medical Centre Utrecht, Obstetrics<br>Prins, Jelmer; University Medical Center Groningen, Department of Obstetrics and Gynaecology<br>Al-Nasiry, Salwan; Maastricht UMC+, Department of Obstetrics and Gynaecology<br>Lutke Holzik, Margo; Leiden Universitair Medisch Centrum, Department of Obstetrics and Gynecology<br>Lopriore, Enrico; Leiden University Medical Center, Department of neonatology and fetal medicine<br>van Drongelen, J. ; Radboud Universiteit, Department of Obstetrics and Gynecology<br>Knol, H. ; Isala Zwolle, Department of Obstetrics and Gynaecology<br>van Laar, J.; Máxima Medical Center, Department of Obstetrics & Gynaecology<br>Jacquemyn, Y.; University Hospital Antwerp<br>van Holsbeke, Caroline; AZ Sint-Lucas Brugge<br>Dehaene, Isabelle; University Hospital Ghent,<br>Lewi, Liesbeth; KU Leuven University Hospitals Leuven, Fetal Medicine Unit<br>van der Merwe, Hannes; KU Leuven University Hospitals Leuven<br>Gyselaers, Wilfried; Hospital Oost-Limburg, Department of Obstetrics and Gynecology<br>Obermann-Borst, Sylvia; Care4Neo, Neonatal patient and parent advocacy organization.<br>Holthuis, Mayella; Patiëntenorganisaties Extreme Vroeggeboorte<br>Mol, Ben; Monash Medical School, OB/GYN<br>Pajkrt, Eva; Amsterdam UMC Location AMC, Obstetrics and Gynaecology<br>Oudijk, Martijn; AMC, Obstetrics

**Primary Subject Heading**:	Obstetrics and gynaecology
Secondary Subject Heading:	Obstetrics and gynaecology
Keywords:	Fetal medicine < OBSTETRICS, Maternal medicine < OBSTETRICS, OBSTETRICS

SCHOLARONE™ Manuscripts

Study protocol for two randomized controlled trials evaluating the effect of cerclage in the reduction of extreme preterm birth and perinatal mortality in twin pregnancies with a short cervix or dilatation: the TWIN Cerclage studies.

Authors

Drs. Lissa van Gils [1]

Dr. Marjon A. de Boer [1]

Prof. Dr. J. Bosmans [2]

Dr. Ruben Duijnhoven[1]

Dr. Sam Schoenmakers [3]

Dr. Jan B. Derks [4]

Dr. Jelmer R. Prins [5]

Dr. Salwan Al-Nasiry [6]

Dr. Margo Lutke Holzik [7]

Dr. Enrico Lopriore [8]

Dr. Joris van Drongelen [9]

Dr. Marieke H. Knol [10]

Dr. Judith O.E.H. van Laar [11]

Prof. Dr. Yves Jacquemyn [12]

Dr. Caroline van Holsbeke [13]

Dr. Isabelle Dehaene [14]

Prof. Dr. Liesbeth Lewi [15]

Dr. Hannes van der Merwe [15]

Dr. Wilfried Gyselaers [16]

Dr. Sylvia A. Obermann-Borst [17]

Mayella Holthuis [18]

Prof. Dr. Ben W. Mol [19]

Prof. Dr. Eva Pajkrt [1]

Prof Dr. Martijn A. Oudijk [1]

For peer review only - http://bmjopen.bmj.com/site/about/guidelines.xhtml

Affiliations

[1] Department of Obstetrics and Gynaecology, Amsterdam University Medical Center, De Boelelaan 1118, 1081 HZ Amsterdam, The Netherlands

[2] Department of Health Sciences and the EMGO Institute for Health and Care Research, Faculty of Earth and Life Sciences, Vrije Universiteit Amsterdam, De Boelelaan 1085, 1081 HV Amsterdam, The Netherlands

[3] Department of Obstetrics and Gynaecology, Erasmus Medical Center, 's- Gravendijkwal 230, 3015 CE Rotterdam, The Netherlands

[4] Department of Obstetrics and Gynaecology, Birth Centre Wilhelmina Children Hospital, University Medical Center Utrecht, Heidelberglaan 100, 3584 CX Utrecht, The Netherlands

[5] Department of Obstetrics and Gynaecology, University Medical Center Groningen, Hanzeplein 1, 9713 GZ Groningen, The Netherlands

[6] Department of Obstetrics and Gynaecology, Maastricht University Medical Center, P. Debyelaan 25, 6229 HX Maastricht, The Netherlands

[7] Department of Obstetrics and Gynaecology, Leiden University Medical Center, Albinusdreef 2, 2333 ZA Leiden, The Netherlands

[8] Department of neonatology and fetal medicine, Leiden University Medical Center, Albinusdreef 2, 2333 ZA Leiden, The Netherlands

[9] Radboud University Medical Center, Department of Obstetrics and Gynaecology, Geert Grooteplein-Zuid 10, 6525 GA Nijmegen, The Netherlands

[10] Department of Obstetrics and Gynaecology, Isala Zwolle, Zwolle, Netherlands

[11] Department of Obstetrics and Gynaecology, Maxima Medical Center in Veldhoven, De Run 4600, 5504 DB Veldhoven, The Netherlands

[12] Department of Obstetrics and Gynaecology, University Hospital, Antwerpen, Belgium

[13] Department of Obstetrics and Gynaecology, AZ Sint-Lucas, Brugge, West-Vlaanderen, Belgium

[14] Department of Obstetrics and Gynaecology, University Hospital Ghent, Corneel Heymanslaan 10, Gent, BE 9000

[15] Leuven University Hospitals Leuven, Fetal Medicine Unit Leuven, Flanders, BE

[16] Hospital Oost-Limburg, Department of Obstetrics and Gynaecology, Genk, BE

[17] Care4Neo, Neonatal patient and parent advocacy organization.Rotterdam, NL

[18] Patiëntenorganisaties Extreme Vroeggeboorte, Amsterdam, NL

[19] Department of Obstetrics and Gynaecology, Robinson Research Institute, University of Adelaide, 72 King William St, North Adelaide, SA 5006 Australia

For peer review only - http://bmjopen.bmj.com/site/about/guidelines.xhtml

Administrative information

Primary Registry and trial identifying number : The trials are registered at www.clinicaltrials.gov with ID NCT05968794. Date of registration in primary registry 01-08-2023.

Secondary Identifying Numbers:

- Protocol ID assigned by Amsterdam University Medical Center NL82609.018
- Research Ethics Committees (REC) in the Netherlands reference number: 2022.0885 - NL82609.018.22.

Trial sponsor: Amsterdam University Medical Center, De Boelelaan 1118, 1081 HZ Amsterdam, The Netherlands. Role of coordinating centre which will perform the analysis and interpreted the data. All members of the TWIN Cerclage project group will participate in writing the report of the final analysis and the decision to submit the report for publication.

Corresponding author: L. van Gils, Department of Obstetrics and Gynaecology, Amsterdam University Medical Center, De Boelelaan 1118, 1081 HZ Amsterdam, The Netherlands. L.vangils1@amsterdamumc.nl

Contact for Public Queries: L. van Gils, Department of Obstetrics and Gynaecology, Amsterdam University Medical Center, De Boelelaan 1118, 1081 HZ Amsterdam, The Netherlands. TWINC@Amsterdamumc.nl

Contact for Scientific Queries: L. van Gils, Department of Obstetrics and Gynaecology, Amsterdam University Medical Center, De Boelelaan 1118, 1081 HZ Amsterdam, The Netherlands. (+31)642811240. TWINC@Amsterdamumc.nl

Recruitment Status: Participants are currently being recruited and enrolled. Start date: 16-05-2023.

Study completion (estimated): 01-12-2028

Protocol version number: Version 1 (6 November 2023).

For peer review only - http://bmjopen.bmj.com/site/about/guidelines.xhtml

Abstract

Introduction: Twin pregnancies have a high risk on extreme preterm birth (PTB) at less than 28 weeks of gestation which is associated with increased risk of neonatal morbidity and mortality. Currently there is a lack of effective treatments in women with a twin pregnancy and a short cervix or cervical dilatation. A possible effective surgical method to reduce extreme PTB in twin pregnancies with an asymptomatic short cervix or dilatation at midpregnancy is the placement of a vaginal cerclage.

Methods and analysis: We designed two international multicentre randomized trials spanned across eight hospitals in the Netherlands and five hospitals in Belgium. Women above the age of 16 years with a twin pregnancy < 24 weeks of gestation and an asymptomatic short cervix ≤ 25mm or cervical dilatation, will be randomly allocated (1:1) in both trials to vaginal cerclage or standard treatment according to the current Dutch NVOG guideline (no cerclage). Permuted blocks will be used of sizes 2 and 4 to minimize the risk of disbalance. The primary outcome measure is PTB <28 weeks. Analyses will be by intention to treat. The first trial is to demonstrate a risk reduction from 25% to 10% in the short cervix group, for which 194 patients need to be recruited. The second trial is to demonstrate a risk reduction from 80% to 35% in the dilatation group and will need to recruit 44 women. A cost-effectiveness analysis will be performed from a societal perspective.

Ethics and dissemination: This study has been approved by the Research Ethics Committees (REC) in the Netherlands on 3/30/2023. The Research Ethics Committees (REC) in Belgium is currently reviewing the study. The results will be presented at conferences and published in a peer-reviewed journal. Participants will be informed about the results.

Trial registration number: The trials are registered at www.clinicaltrials.gov with ID NCT05968794.

Strengths and limitations of this study

- These are the largest randomized trials comparing the effect of cerclage on PTB in women with a twin pregnancy and a short cervix or dilatation.
- A strength of the study lies in its comprehensive assessment of outcomes, including preterm birth at various gestational age thresholds, as well as a thorough examination of adverse maternal and neonatal outcomes.
- The Intention to perform long term follow-up of children, thus future parents can be counselled on the long-term effects of this intervention, enhances the strength of this study
- The recruitment of patients could be a potential challenge as the incidence of women with a twin pregnancy and a short cervix ≤ 25 mm or dilatation at midpregnancy is low.

Key-words: Preterm birth, twins, cerclage, randomized controlled trial

Introduction

For peer review only

Twin pregnancies have a high risk on extreme preterm birth (PTB) at less than 28 weeks of gestation which is associated with increased risk of neonatal morbidity and mortality.(1) Worldwide, twins represent 0.5–4.0% of all births, with rates varying between ethnic groups and countries.(2) Compared with singletons, twins are at increased risk for adverse perinatal outcomes.(3) In the Netherlands, 250 (5,2%) women with a twin pregnancy deliver at < 28 weeks per year, resulting in 157 perinatal deaths and 343 surviving neonates of whom a large proportion suffer from long term neurodevelopmental problems associated with extreme preterm birth.(4) An asymptomatic short cervix (≤ 25 mm) or dilatation at midpregnancy in twin pregnancies is associated with high rates of extreme PTB and proven to be the best predictor for extreme PTB.(5, 6)

Effective interventions for twin pregnancies with short cervix are limited. The EPPPIC individual participant data (IPD) meta-analysis on progesterone treatment in multiple pregnancies with a short cervix showed no reduction of PTB, however a recent IPD meta-analysis did show a possible effect of progesterone.(7, 8) The use of a vaginal pessary in women with a twin pregnancy and a short cervix does not seem to result in a reduction of PTB. (9)

A possible surgical method to reduce extreme PTB in twin pregnancies with an asymptomatic short cervix or dilatation at midpregnancy is a vaginal cerclage. An IPD meta-analysis from 2015 assessed its impact in 49 women with a twin pregnancy and a short cervix (< 25 mm) across three randomized controlled trials.(10) No significant differences were present between the groups on outcomes such as PTB or perinatal death. However, a more recent systematic review with a total of 471 women also included six cohort studies that focused on the outcome of PTB at different cut-offs.(11) The pooled results of these cohort studies showed a significant beneficial effect of a cerclage on PTB <32 weeks (Relative risk (RR) 0.68; 95% confidence interval (CI) 0.51-0.92) and <34 weeks (RR 0.73; 95% CI 0.59-0.90). In addition, a cerclage significantly prolonged pregnancy (mean 2.53 weeks (95% CI 1.25-3.81)). No significant difference was seen in the number of PTBs <24 and 28 weeks or perinatal mortality, although the trend was in favour of the cerclage group.

Furthermore, a subgroup analysis by cervical length was carried out, distinguishing between a cervical length between 16 and 24mm and ≤15mm.(11) For the group with a cervical length <15mm, cerclage placement was associated with less PTB <37 weeks (RR 0.86; 95% CI 0.74-0.99), <34 weeks (RR 0.57; 95% CI 0.43-0.75) and <32 weeks (RR 0.61; 95% CI 0.41-0.90) compared to the control group. Cerclage also resulted in a significant pregnancy prolongation by 3.89 weeks (95% CI 2.19-5.59). In the group of women with a cervical length of 16-24mm, no significant differences were seen in the outcomes.

In women with a twin pregnancy and asymptomatic cervical dilatation, the most recent systematic review was published in August 2023 and included 1 RCT and 5 retrospective cohort studies.(12) These studies showed that a vaginal cerclage may result in a reduction of PTB at less than 24 weeks (RR 0.32; 95% CI 0.21-0.48), 28 weeks (RR 0.47; 95% CI 0.36-0.62), 32 weeks (RR 0.59; 95% CI 0.50–0.70) and 34 weeks (RR 0.68; 95% CI 0,57–0,80). In addition, women with a cerclage had a reduced risk of perinatal death (RR 0.30; 95% CI 0.16-0.55), 5 minute Apgar score < 7 (RR 0.49; 95% CI 0.27-0.90), birthweight <1500 grams (RR 0.41; 95% CI 0.31-0.55) and admission to the neonatal intensive care unit (NICU) (RR 0.67; 95% CI 0.52-0.88).

In conclusion, there is a lack of well-designed RCT's on the effect of vaginal cerclage, on PTB and neonatal outcomes in women with an twin pregnancy with a short cervix or asymptomatic dilatation

For peer review only - http://bmjopen.bmj.com/site/about/guidelines.xhtml

at midpregnancy. Long term follow-up of children is lacking in general, thus future parents cannot be counselled on the long term effects of this intervention. The possible effect of a cerclage in cohort studies justifies a well-designed large multicentre randomized trial. We therefore will perform the TWIN Cerclage study.

Objectives: To assess the effectiveness of a vaginal cerclage in women pregnant with a twin pregnancy with a midpregnancy asymptomatic short cervix or dilatation in the prevention of extreme PTB and perinatal mortality compared to standard treatment (no cerclage).

Methods and analysis

Design and setting

We will conduct two international multicentre randomized controlled trials within the Dutch Consortium for Healthcare Evaluation and Research in Obstetrics and Gynaecology (total of eight hospitals) and participating five hospitals in Belgium.

Participants / eligibility criteria

RCT I: Women (> 16 years of age) with a twin pregnancy with a short cervix ≤ 25 mm below 24 weeks of pregnancy are eligible for this trial.

RCT II: Women (> 16 years of age) with a twin pregnancy with dilatation on transvaginal ultrasound or physical examination below 24 weeks of pregnancy are eligible for this trial.

Women who meet any of the following criteria will be excluded from participation in these trials:

- Women with a mono-amniotic twin pregnancy
- Women with twin pregnancy in which one or both children are diagnosed with a major structural, congenital or chromosomal abnormality (at time of inclusion) that is likely to influence the composite adverse neonatal outcome.
- Women with signs of clinical intra-uterine infection, defined by the presence of fever ≥ 38 degrees Celsius.
- Women with overt symptoms of preterm labour at time of measurement of short cervix or dilatation (regular contractions, PPROM, recurrent blood loss).
- Women with a placenta previa, defined as a placenta position covering the internal ostium of the cervix.
- Women who do not master the Dutch or English language and are therefore not able to give written consent.

Sample size calculation

In women with a twin pregnancy and an asymptomatic short cervix, the sample size was calculated based on a meta-analysis published in 2019.(11) In this study, the incidence of preterm birth before 28 weeks was lower in the cerclage group (13.8%) compared to the expected management group (23.0%) (RR 0.64; CI 0.40-1.02).

For peer review only

• Stratum I Short cervix group: To demonstrate a risk reduction from 25% to 10%, 97 patients need to be recruited per arm (194 in total). Sample size for this stratum was calculated using a Z-test with unpooled variance, a two-sided alpha of 0.05 and a target power of 80%.

In women with a twin pregnancy and asymptomatic cervical dilatation, the sample size was calculated based on a systematic review published in December 2021, which included 1 RCT and 4 retrospective cohort studies.(13) In this study, the incidence of preterm birth before 28 weeks was significantly lower in the cerclage group (37.2%) compared to the expected management group (76.9%) (RR 0.44; CI 0.33-0.58).

• Stratum II Dilatation group: To demonstrate a risk reduction from 80% to 35%, 22 patients need to be recruited per arm (44 in total). The sample size for this stratum was calculated using Fisher's exact test, with a two-sided alpha of 0.05 and a target power of 80%. Numbers are not corrected for loss of follow-up, as it is highly unlikely to have loss to follow-up in these high risk pregnancies.

Treatment

The investigated intervention is a vaginal cerclage. Potential complications of a cerclage are infection, premature rupture of membranes, cervical laceration or bleeding and anaesthesia-related complications, occurring in approximately 0.3-2.5 %.(14)

The comparator will be standard treatment according to the current guideline in the Netherlands from 2018, which is to not perform or offer an intervention such as vaginal cerclage.(15) This is in line with the current standard of care in most of the participating hospitals in Belgium.

Study procedures

Measurement of cervical length in twin pregnancies will be performed as part of the standard 20 week ultrasound scan in all hospitals in the Netherlands and participating centres in Belgium. In case of a short cervical length or dilatation patients will be seen at the nearest participating centre, where women will be counselled for participation in the study. Inclusion is possible below 24 weeks of gestation, and if randomized to the surgical treatment, the cerclage needs to be placed before 24^{+0} weeks of gestation.

After women give written consent to participate in the trials, patients will be randomized for two treatment options: 1. surgical treatment with cerclage and additional standard care according to Dutch guidelines or 2. Standard care according to Dutch guidelines (no cerclage). Randomization will be done using a web-based interface for each of the two trials. For the trial recruiting women with dilatation, random permuted blocks will be used of sizes 2 and 4 to minimize the risk of disbalance. Participants and investigators will not be blinded for the intervention.

In addition, participants are treated according to the local protocol in the participating clinics i.e. the use of progesterone during pregnancy and other interventions such as tocolysis and corticosteroids in case of a threatened PTB. Follow up of the pregnancy and delivery will be in the referral centre until delivery. The cerclage will be removed according to protocol between 36-37 weeks of gestation, or in case of labour, whatever comes first. Delivery will take place by either spontaneous onset of labour, or induction of labour or elective caesarean section according to national guidelines for twin pregnancies.

For peer review only - http://bmjopen.bmj.com/site/about/guidelines.xhtml

The referral hospitals will be contacted for data collection of outcomes as mentioned above by the studies' research midwifes or nurses. Primary and secondary outcome measures will be obtained from clinical patient data. Outcomes such as PTB will be assessed at birth, additional outcomes will be assessed at the time of discharge of the child from hospital to home address and at 3 months after discharge.

Outcome measures

Main study parameter/endpoint

The primary outcome will be extreme preterm birth < 28 weeks.

Secondary study parameters/endpoints

Secondary outcomes include:

- A composite for adverse neonatal outcome (including bronchopulmonary dysplasia, periventricular leucomalacia > grade 1, intraventricular hemorrhage > grade 2, necrotizing enterocolitis > stage 2, retinopathy of prematurity > stage 2, proven sepsis and perinatal death)
- All components of the composite outcome will also be assessed separately.
- Preterm birth (indicated and spontaneous) at less than 24, 32, 34 and 37 weeks.
- Premature rupture of membranes (PPROM)
- Gestational age at delivery
- Days on ventilation support
- Days in NICU
- Maternal quality of life
- Maternal outcomes, including sepsis, need for antibiotics, need to remove cerclage in operation room, mode of delivery (% caesarean delivery).
- Healthcare costs

Outcome parameters are in line with the core outcome set for studies on prevention of PTB defined by members of GONet and the Core Outcomes in Women's health (CROWN) initiative.(16)

Parents will be asked consent to approach the parents for long-term follow-up of the children at 2 and 4 years of age. Maternal quality of life will be assessed at inclusion, discharge after birth and 3 months postpartum using the five level version of the EuroQol (EQ-5D-5L).(17) Costs will be measured from a societal perspective using web-based questionnaires based on the iMCQ and iPCQ (Medical and Productivity Cost Questionnaires) at discharge after birth and at 3 months after discharge for the mother and the neonate.

In addition, pregnancy outcome data will also be collected for women opting not to participate in the trials but giving informed consent to collect data on pregnancy outcome from hospital records. Women give permission for this on a voluntary basis.

Randomisation and treatment allocation

Randomization will be done using a web-based interface a member of de project team or a qualified research nurse. Randomization will be stratified for the presence of dilatation at time of recruitment.

For peer review only

For peer review only - http://bmjopen.bmj.com/site/about/guidelines.xhtml

Allocation will be 1:1 and permuted blocks will be used of sizes 2 and 4 to minimize the risk of disbalance. Participants and investigators will not be blinded for the intervention.

Withdrawal of subjects

Subjects can leave the study at any time for any reason if they wish to do so, without any consequences. Patients who withdraw from the study will remain in their treatment group for the intent-to-treat analysis, their data will be used for analysis. The investigator can decide to withdraw a subject from the study for urgent medical reasons.

Follow-up of subjects withdrawn from treatment

As the statistical analysis is planned according to intention to treat principle, patients that discontinue the intervention will be analysed in the group that they were allocated to.

Safety reporting

Adverse events (AEs)

An adverse event is defined as an event during or following medicinal treatment or follow up which was not intended to happen and is suspected to be a complication of the intervention performed.

Serious adverse events (SAEs)

Serious Adverse Events (SAEs) will be reported from the first study-related procedure until 3 months after delivery. A serious adverse event is any untoward medical occurrence or effect that:

• results in death (maternal or perinatal)

• is life threatening (at the time of the event);

• requires hospitalisation or prolongation of existing inpatients' hospitalisation

• results in persistent or significant disability or incapacity

• any other important medical event that did not result in any of the outcomes listed above due to medical or surgical intervention but could have been based upon appropriate judgement by the investigator

SAE's must be reported by e-mail to the study project team within 24 hours after the event was reported to the investigator, using the provided SAE report form. This initial report should minimally contain information with respect to the event, associated treatment and patient identification, as described in the detail in the instructions for the SAE report form. If necessary, more detailed information should be provided in a follow-up report within a further 2 business days. SAE's need to be reported until end of study as defined in the protocol. SAE's that result in death or are life threatening should be reported immediately with a maximal 7 days for a preliminary report with another 8 days for completion of the report. All other SAEs will be reported within a period of maximum 15 days after the sponsor initially received knowledge of the serious adverse events.

If required by national laws or regulations or by the procedures of the authorities, the sponsor will ensure that a six-monthly line listing of all reported SAEs is submitted to the Ethics Committee(s).

For peer review only

For peer review only - http://bmjopen.bmj.com/site/about/guidelines.xhtml

Events inherent to the study population

Due to the fact that the population is pregnant with a notable risk of premature birth, events inherent to the study population are to be expected and will not be considered as SAE. The study project group will report these events by line listing to the Health Authorities, competent authorities, and MREC once a year.

The following events are not considered to be a Serious Adverse Event:

• Maternal hospital admission during pregnancy, delivery or postpartum (with exception of admission to the Intensive Care Unit or Coronary Care Unit, which will be considered as SAE's)

• Maternal hospitalization for a procedure that was planned prior to study participation (i.e. prior to registration or randomization). This should be recorded in the source documents.

• Prolonged maternal hospitalization for a complication of such procedures remains a reportable serious adverse event.

• Neonatal NICU admission

• Neonatal hospital admission other than NICU admission

• Neonatal disease expected in prematurity such as: bronchopulmonary dysplasia, periventricular leucomalacia, intraventricular hemorrhage, necrotizing enterocolitis, retinopathy of prematurity, culture proven sepsis.

Follow-up of (serious) adverse events

Adverse events occurring from the first study-related procedure until 3 months after delivery will be recorded in the CRF. All AEs will be followed until they have abated, or until a stable situation has been reached. Depending on the event, follow up may require additional tests or medical procedures as indicated, and/or referral to the general physician or a medical specialist. All serious adverse events will be followed clinically until they are resolved or until a stable situation has been reached. Depending on the event, follow up may require additional tests or medical procedures as indicated and/or referral to a general physician or a medical specialist. Follow-up information on SAEs should be reported until recovery or until a stable clinical situation has been reached. The final outcome of the SAE should be reported on a final SAE report.

Monitoring and safety

An independent data safety monitoring board (DSMB) will monitor the trials for safety. The DSMB consists of several members with expertise in the relevant fields of obstetrics, neonatology, epidemiology and statistics. Serious adverse events (SAEs) will be collected from the first study-related procedure until 3 months after delivery. SAE's must be reported by e-mail to the study project team within 24 hours after the event was reported to the investigator.

An interim analysis will be conducted for safety (i.e. interim safety review) at least after the outcomes for the first 50 and 100 patients have become available in RCT I (short cervix), or the first 22 patients (half the planned sample size) in RCT II (dilatation group).

For peer review only

Premature termination of the study

The DSMB can advise to terminate the study prematurely in case of safety concerns. The DSMB can advise to terminate the study prematurely in case the interim analysis shows harm of either one of the interventions (cerclage or no cerclage) or due to external evidence.

An interim analysis will be conducted for safety (i.e. interim safety review) after the outcomes for the first 50 patients have become available for stratum I, and the first 22 patients (half the planned sample size) in stratum II. In addition, after the outcomes of 100 patients have come available from stratum I, the DSMB will meet again for interim safety review.

Statistical analysis

Data analysis will be performed according to the intention-to-treat principle. A supplementary per-protocol analysis is considered if any patients in the standard care arm would have received a cerclage (protocol deviation). Categorical variables will be expressed as a number with the percentage of the total allocation arm. Continuous variables will be presented as mean with standard deviation, as geometric mean with 95% confidence interval (CI) or as median with interquartile range, whenever appropriate.

The primary outcome will be evaluated using the intention-to-treat population. Estimand for both RCT I and RCT II is the relative risk for extreme PTB after cerclage, as compared to the control group not undergoing cerclage. Confidence interval will be a two-sided 95% interval, with statistical inference based on the Chi-squared test for RCT I, and Fisher's exact test for RCT II. Heterogeneity of treatment effect is anticipated to be substantial for the two trials, and a pooled estimate will only be provided would heterogeneity be low. Heterogeneity will be assessed using a forest plot and tests for interaction in a generalized linear model. The two trials may be reported separately if not completed within a reasonable time from each another to allow timely communication of results.

Missing data for the primary outcome will be very low, given that the primary outcome is both an outcome recorded for routine care and there is a short follow-up time. If patients would deliver in other hospitals than the hospital they were recruited from, the data can still be obtained. Only patients who actively revoke consent cannot be analysed for the primary outcome.

The secondary outcome measures be approached similarly to the primary outcome measure. Dichotomous outcomes will be presented as relative risks and absolute with 95% confidence intervals and chi-squared tests or Fisher's exact test. Continuous secondary outcomes, differences between groups will be assessed with the student's t-test if the outcome is normally distributed and with a non-parametric Mann-Whitney U test if skewed. These outcomes will be presented as means with standard deviation, geometric means with 95% CI, or as median with interquartile range, whichever appropriate. A nominal level of 5% will be used for significance. From the perspective of type I error control the two trials are considered as independent tests.

Subgroup analyses

The following subgroup analyses are planned:

- Cervical length ≤ 15 mm and 16-25 mm
- Dichorionic and monochorionic pregnancies
- Multiparous women with a history of PTB and women without a history of PTB

For peer review only - http://bmjopen.bmj.com/site/about/guidelines.xhtml

- Nulliparous women and multiparous women with a term birth in their obstetric history

Economic evaluation

Both a cost-effectiveness analysis (CEA) and a cost-utility analysis (CUA) will be performed from a societal and healthcare perspective according to Dutch guidelines with a time horizon of 3 months after discharge. All statistical analyses will be done according to the intention-to treat principle. Missing cost and effect data will be imputed using multiple imputation according to the MICE algorithm. Rubin's rules will be used to pool the results from the different multiply imputed datasets. Linear regression analyses will be used to estimate cost and effect differences between intervention and control while adjusting for confounders if necessary. Incremental cost-effectiveness ratios (ICERs) will be calculated by dividing the difference in the mean total costs between the treatment groups by the difference in mean effects between the treatment groups. Bias-corrected and accelerated bootstrapping with 5000 replications will be used to estimate 95% confidence intervals around the cost differences and statistical uncertainty surrounding the ICERs. Uncertainty surrounding the ICERs will be graphically presented on cost-effectiveness planes. Cost-effectiveness acceptability curves will also be estimated showing the probability that the intervention is cost-effective in comparison with control for a range of different ceiling ratios thereby showing decision uncertainty.(18)

Budget impact analysis

A budget impact analysis (BIA) will be conducted from the perspective of health-care decision makers according to the Dutch guidelines and the recommendations from Sullivan et al using the Dutch BIA tool.(19) In the BIA, data from the effectiveness and cost-effectiveness analyses regarding the differences in costs and health outcomes will be combined with national prevalence and incidence data to extrapolate the findings to a time horizon of 5 years. The BIA will be conducted from the government perspective (Budget Kader Zorg) and the societal perspective. Actual NZA tariffs will be used to calculate costs. The budget analysis will differentiate between incidental and structural cost (savings), and will take into account budgetary consequences of changes within these cost components. Sensitivity analyses will be performed for subgroups of patients if applicable, providing budget information for relevant subgroups to decision makers. In addition, sensitivity analyses will address the impact of variations of the main assumptions and input parameters for the BIA.

Patient and public involvement

The Dutch consortium collaborates with three Dutch patient associations, Care4Neo (Neonatal patient and parent advocacy organization), the 'Vereniging voor Ouders van Meerlingen (NVOM)' (society of parents of multiples) and the patient organization for extreme PTB. They are project members of the TWINC Cerclage study and involved in the design of the study. A survey was performed during the design of the study among members of the closed Facebook group of both patient associations, to evaluate the interest in participation in the TWIN Cerclage study. The Dutch consortium has a website where it publishes all results of completed studies, and publishes the protocols of currently recruiting studies.

For peer review only - http://bmjopen.bmj.com/site/about/guidelines.xhtml

Ethics and dissemination

The Research Ethics Committee (REC) at the Amsterdam University Medical Centres approved this study (reference number: 2022.0885 - NL82609.018.22). Additional regional approval was obtained for the remaining participating hospitals in The Netherlands. The Research Ethics Committees (REC) in Belgium is currently reviewing the study. Protocol amendments will be communicated to relevant parties. A manuscript with the results of the primary study will be published in a peer-reviewed journal and presented at conferences. A separate manuscript will be written on the cost-effectiveness analysis.

Recruitment and consent

Eligible participants will receive the participant information leaflet and will be informed about the study during an outpatient clinic.(Supplementary file 1) Because of the need to initiate treatment as soon as possible (<24 weeks of pregnancy) we will call the subject in 1 to 5 days (based upon her gestational age at time of counselling) get the opportunity to ask questions to ask if she wants to participate in the study. If the participants wants to participate, she will be ask to sign the informed consent and ask to send a photo of her signed informed consent. To schedule the cerclage randomization will be performed after receiving a copy of the informed consent. If women are randomized to the cerclage group, they will be ask to bring the original signed informed consent at the day of their admission for the cerclage. If women randomize for standard care, they will be ask to send the original signed informed consent back with the return envelope.

Public disclosure and publication policy

The study protocol and results will be published in peer-reviewed, indexed, journals using an open access format, and the results will be presented at academic conferences. Authorship eligibility will be in accordance with The International Committee of Medical Journal Editors.

Administrative aspects and monitoring

Handling and storage of data and documents

Data will be collected using Castor EdC. Data monitoring will be done by the primary investigator. Data processing will be done coded, as the patient code only available to the local investigators. All data will be stored for 15 years in a secured database on a secured computer, to which only the investigators will have access. All personal data is protected conform the rules of the General Data Protection Regulation. Data on ethnicity and level of education will be collected from the patients medical file because this is one of the known risk factors for PTB. The participant will give her consent for this on the consent form.(Supplementary file 1)

Monitoring and Quality Assurance

Data monitoring will be performed by a certified clinical research associate of the participating institute or its delegate. The monitor will compare the data entered into the database with the hospital or clinic records (source documents). The nature and location of all source documents will be identified to ensure that all sources of original data required to complete the database are known to the investigational staff and are accessible for verification. Frequency of monitoring will be further discussed with the monitoring party.

For peer review only - http://bmjopen.bmj.com/site/about/guidelines.xhtml

Recruitment Status: Participants are currently being recruited and enrolled. Start date: 16-05-2023.

Study completion (estimated): 01-12-2028

Competing interests: None.

Funding: This study is funded by ZonMw (The Netherlands organisation for health research and development), grant number 10390012110017.

Contributorship: Drs. Lissa van Gils, Dr. Marjon A. de Boer, Prof. Dr. J. Bosmans, Dr. Ruben Duijnhoven, Dr. Sam Schoenmakers, Dr. Jan B. Derks, Dr. Jelmer R. Prins, Dr. Salwan Al-Nasiry, Dr. Margo Lutke Holzik, Dr. Enrico Lopriore, Dr. Joris van Drongelen, Dr. Marieke H. Knol, Dr. Judith O.E.H. van Laar , Prof. Dr. Yves Jacquemyn, Dr. Caroline van Holsbeke, Dr. Isabelle Dehaene, Prof. Dr. Liesbeth Lewi, Dr. Hannes van der Merwe, Dr. Wilfried Gyselaers, Dr. Sylvia A. Obermann-Borst, Mayella Holthuis, Prof. Dr. Ben W. Mol, Prof. Dr. Eva Pajkrt and Prof Dr. Martijn A. Oudijk were all involved in the conception and design of this study. All authors mentioned in the manuscript are member of the TWIN Cerclage study group. Dr. Sam Schoenmakers, Dr. Jan B. Derks, Dr. Jelmer R. Prins, Dr. Salwan Al-Nasiry, Dr. Margo Lutke Holzik, Dr. Enrico Lopriore, Dr. Joris van Drongelen, Dr. Marieke H. Knol, Dr. Judith O.E.H. van Laar , Prof. Dr. Yves Jacquemyn, Dr. Caroline van Holsbeke, Dr. Isabelle Dehaene, Prof. Dr. Liesbeth Lewi, Dr. Hannes van der Merwe and Dr. Wilfried Gyselaers are local investigators in the participating centres. Dr. Sylvia A. Obermann-Borst and Mayella Holthuis represent two Dutch patient associations (Care4Neo and the patient organization for extreme PTB). Drs. Lissa van Gils and Prof. Dr. Martijn A. Oudijk drafted this manuscript. All other authors reviewed and edited the manuscript. All authors approved the final version of this protocol and agreed to be accountable for all aspects of the work in ensuring that questions related to the accuracy or integrity of any part of the work are appropriately investigated and resolved.

References

1. Schaaf JM, Mol BW, Abu-Hanna A, Ravelli AC. Trends in preterm birth: singleton and multiple pregnancies in the Netherlands, 2000-2007. BJOG. 2011;118(10):1196-204.
2. Deng C, Dai L, Yi L, Li X, Deng K, Mu Y, et al. Temporal trends in the birth rates and perinatal mortality of twins: A population-based study in China. PLoS One. 2019;14(1):e0209962.
3. Russo FM, Pozzi E, Pelizzoni F, Todyrenchuk L, Bernasconi DP, Cozzolino S, Vergani P. Stillbirths in singletons, dichorionic and monochorionic twins: a comparison of risks and causes. Eur J Obstet Gynecol Reprod Biol. 2013;170(1):131-6.
4. data P. Perinatale registratie Nederland. 2019.
5. Honest H, Bachmann LM, Coomarasamy A, Gupta JK, Kleijnen J, Khan KS. Accuracy of cervical transvaginal sonography in predicting preterm birth: a systematic review. Ultrasound Obstet Gynecol. 2003;22(3):305-22.
6. Lim AC, Hegeman MA, Huis In TVMA, Opmeer BC, Bruinse HW, Mol BW. Cervical length measurement for the prediction of preterm birth in multiple pregnancies: a systematic review and bivariate meta-analysis. Ultrasound Obstet Gynecol. 2011;38(1):10-7.
7. Group E. Evaluating Progestogens for Preventing Preterm birth International Collaborative (EPPPIC): meta-analysis of individual participant data from randomised controlled trials. Lancet. 2021;397(10280):1183-94.
8. Romero R, Conde-Agudelo A, Rehal A, Da Fonseca E, Brizot ML, Rode L, et al. Vaginal progesterone for the prevention of preterm birth and adverse perinatal outcomes in twin gestations with a short cervix: an updated individual patient data meta-analysis. Ultrasound Obstet Gynecol. 2022;59(2):263-6.

For peer review only - http://bmjopen.bmj.com/site/about/guidelines.xhtml

9. Hoffman M. Randomized trial of cervical pessary for short cervix in singleton pregnancies. American journal of obstetrics and gynecology. 2023;228(1):S5-S6.
10. Saccone G, Rust O, Althuisius S, Roman A, Berghella V. Cerclage for short cervix in twin pregnancies: systematic review and meta-analysis of randomized trials using individual patient-level data. Acta Obstet Gynecol Scand. 2015;94(4):352-8.
11. Li C, Shen J, Hua K. Cerclage for women with twin pregnancies: a systematic review and metaanalysis. Am J Obstet Gynecol. 2019;220(6):543-57 e1.
12. D'Antonio F, Eltaweel N, Prasad S, Flacco ME, Manzoli L, Khalil A. Cervical cerclage for prevention of preterm birth and adverse perinatal outcome in twin pregnancies with short cervical length or cervical dilatation: A systematic review and meta-analysis. PLoS medicine. 2023;20(8):e1004266.
13. Su J, Li D, Yang Y, Cao Y, Yin Z. Cerclage placement in twin pregnancies with cervical dilation: a systematic review and meta-analysis. J Matern Fetal Neonatal Med. 2022;35(25):9112-8.
14. van Dijk CE, Breuking SH, Jansen S, Limpens J, Kazemier BM, Pajkrt E. Perioperative complications of a transvaginal cervical cerclage in singleton pregnancies: a systematic review and meta-analysis. AMERICAN JOURNAL OF OBSTETRICS AND GYNECOLOGY. 2023;228(5):521-52+.
15. (NVOG) DSfOaGNVvOeG. Preventie-Vroeggeboorte-bij-Meerlingzwangerschappen-def-2018. 2018.
16. van 't Hooft J. A core outcome set for evaluation of interventions to prevent preterm birth: summary for CROWN. BJOG. 2016;123 Suppl 3:107.
17. M MV, K MV, S MAAE, de Wit GA, Prenger R, E AS. Dutch Tariff for the Five-Level Version of EQ-5D. Value Health. 2016;19(4):343-52.
18. Fenwick E, O'Brien BJ, Briggs A. Cost-effectiveness acceptability curves--facts, fallacies and frequently asked questions. Health Econ. 2004;13(5):405-15.
19. Sullivan SD, Mauskopf JA, Augustovski F, Jaime Caro J, Lee KM, Minchin M, et al. Budget impact analysis-principles of good practice: report of the ISPOR 2012 Budget Impact Analysis Good Practice II Task Force. Value Health. 2014;17(1):5-14.

For peer review only

Patient Information – TWIN cerclage study

The TWIN Cerclage Study: *The effectiveness of Cerclage for the reduction of extreme preterm birth and perinatal mortality in Twin pregnancies with a short cervix.*

Dear Madam,

You are asked to participate in a medical scientific study. Participation is voluntary. Your written consent is required to participate. You are receiving this letter because you are pregnant with twins and are at risk of having a preterm delivery. You can read about the medical study in this information sheet, what it means for you, and about the advantages and disadvantages. It's a lot of information. Would you please read the information and decide if you want to participate? If you would like to participate, please fill out the form found in Appendix C.

Ask your questions

You can make your decision with the information you find in this information letter. In addition, we encourage you to:

- Ask the researcher giving you this information any questions you have.
-Talk to your partner, family or friends about this study.
-Pose your questions to the independent expert, see Appendix A for contact information.
-Read the information at www.rijksoverheid.nl/mensenonderzoek

1. General information

This study has been designed by the Department of Obstetrics and Gynecology of the Amsterdam University Medical Center, location AMC. The study will be conducted by gynecologists, clinicians or midwives. A total of 194 pregnant women with a short cervix and 44 women with dilatation of the cervix are required for this study. This study is approved by the Medical Ethics Committee (METC) Academic Medical Centre.

2. Purpose of the study

The purpose of this study is to investigate the effectiveness of a vaginal cerclage in women with a twin pregnancy and a midpregnancy short cervix compared to standard treatment (no cerclage) in the prevention of extreme preterm birth < 28 weeks of gestational age. The effectiveness of a vaginal cerclage will be compared with standard care according to the (inter)national guidelines, no cerclage.

3. Background of the study

Twin pregnancies have a high risk of extreme preterm birth (PTB) at less than 28 weeks of gestation which is associated with increased risk of neonatal morbidity and mortality. In the Netherlands, per year 250 women with a twin pregnancy deliver at < 28 weeks, resulting in 157 perinatal deaths. One of the risk factors for extreme preterm birth is a short cervix. If a short cervix is ≤ 25mm at the 20-week ultrasound, there is a high risk of an extremely premature birth. A possible effective surgical method to reduce extreme PTB in twin pregnancies with a short cervix or asymptomatic dilatation at midpregnancy is a minor operative procedure, the placement of a vaginal cerclage

For peer review only - http://bmjopen.bmj.com/site/about/guidelines.xhtml

4. What does participation mean?

How long will the study last?
Are you participating in the study? Then the study will run until three months after you gave birth. A few years after the study is finished, we may want to contact you to inquire through a questionnaire how you and your child are doing. At that time, you can decide whether you wish to answer our questions. No additional diagnostic tests are performed on you or your baby.

Are you suitable for participation in the study?
Women above the age of 16 years with a twin pregnancy and a short cervix or cervical dilatation below the 24th week of pregnancy can participate. It may happen that you are healthy, but that you are not suitable to participate. The researcher will tell you more about this.

The treatment
If you consent to participate in the study you will be randomly assigned to either the group receiving standard care (no cerclage) or a cerclage. A vaginal cerclage is a minor surgical procedure in which an unabsorbable suture is placed around/through the cervix to close the cervical canal and to increase its firmness, in order to reduce cervical insufficiency and this pathway to preterm birth. The chance to end up in either one of the groups is equal.

Examinations and measurements
During the standard 20-week ultrasound (SEO), the cervix is measured in your own hospital. If the cervix is short (<25mm), you will be referred to a hospital that participates in this study. The participating hospital can decide to measure the cervical length again and if indicated perform physical examination to detect possible cervical dilatation. If you have drawn for vaginal cerclage group, the cerclage will be placed under anesthesia or epidural. You will be admitted to the hospital for one day.

During the study, data about yourself, the pregnancy, the delivery and your children will be collected. For example, data such as your date of birth, ethnicity and level of education. Collecting data on ethnicity is important because people of different ethnicities may have different cervical lengths and may also respond differently to treatment.

Furthermore, we ask you to complete three online questionnaires: at the start of the treatment, after discharge from the hospital after the delivery and three months after delivery. Completing the questionnaires will take approximately 30 minutes. The questionnaires are completed online on a safe and private server. We ask your permission for using your email address to send you the questionnaires.

Follow-up: What is different from regular care?
If you have drawn for standard care, the pregnancy will be followed up by your own gynecologist according to the usual care. If you have drawn for a cerclage, the pregnancy will also be followed up by your own gynecologist with the usual check-ups after placement. . In addition to the treatment, no additional checks are necessary and no additional examinations will be performed. The cerclage will be removed at 36-37 weeks of pregnancy or sooner if labor starts before then.

For peer review only - http://bmjopen.bmj.com/site/about/guidelines.xhtml

5. What do we expect from you?

We strive for the research to proceed as planned. Therefore, we will make the following agreements with you:

- You will come to every control visit
- You contact the investigator if:
    - You are hospitalized
    - You suddenly start having problems with your health
    - You no longer want to participate in the study
    - Your phone number, address or email address has changed

6. What side effects, adverse reactions or discomforts may you experience?

A vaginal cerclage is a minor and safe surgical procedure commonly performed in singleton pregnancies with a short cervix or dilatation and a previous preterm birth in all the participating centers, thus there is experience in the participating hospitals. Potential complications of a cerclage are infection, premature rupture of membranes, cervical laceration or bleeding and anesthesia-related complications, occurring in approximately 0.3-2.5 %. In addition, placing a cerclage is a surgical procedure and there are the small general risks of surgery and anesthesia or an epidural.

7. Possible advantages and disadvantages

It is important to weigh the pros and cons before you decide to participate. It is currently unclear whether a vaginal cerclage reduces preterm birth rates in women with a twin pregnancy and a short cervix. We cannot guarantee you will benefit from participating in this study. The study may however be useful for other pregnant women in the future and help in the search for better treatment of premature birth in twin pregnancies

If you do not wish to participate or wish to stop your participation

Your participation in this study is completely voluntary. If you do not wish to participate, you will be treated according to local protocol (no cerclage). If you decide not to participate, we would like to ask for your consent to collect, use and store data from your pregnancy and childbirth in order to gain insight into the treatment of preterm birth in twin pregnancies. If you decide that we are allowed to collect your data from your pregnancy and childbirth, we would like to ask you to sign the consent statement in Appendix D.

8. The end of the study

If there is new information about the study that is of interest to you, the investigator will inform you. You will then be asked whether you want to continue your participation.

Your participation in the study stops when:

- Three months after you gave birth
- You decide to withdraw from the study. You can decide to stop at any time during the study and you can inform the investigator about this. You do not have to explain why you want to discontinue participation.
- If you physician decides it’s better for you to stop-If the investigator, the government or the judging medical ethical committee decide to stop the study

What happens when you discontinue participation?

The researchers will use your data that is collected until the moment you decide to discontinue the study. If you are assigned to the cerclage group, your physician will discuss your wishes regarding the cerclage. Overall, the study is concluded once all the participants have completed the study.

For peer review only - http://bmjopen.bmj.com/site/about/guidelines.xhtml

9. Usage and storage of your data

Are you participating in the study? If so, then you also give us permission to use and store your personal data.

What personal data do we keep?

- Your and your babies' names
- Your and your babies' genders
- Your address
- Your and your babies date of birth
- Your ethnicity
- Your level of education
- Information about your and your babies' health
- Data about your pregnancy and delivery
- (Medical) data that we collect during the study

Why do we collect, use and store your data?

We collect, use and store your and your children's data to answer the research questions and to be able to publish the results.

How do we protect your privacy?

To protect your privacy, your data is encrypted. All your data can only be traced back to you with a key of the code. The key to the code remains securely stored in the local research facility. When processing your data, we only use this code. The data cannot be traced back to you in reports and publications about the study.

Who will have access to my data?

Some people can access all the data at the study location, including the data without a code. This is necessary to check whether the study is being conducted in a good and reliable manner. Persons who have access to your data for review are:

-Members of the committee that monitors the safety of the study

- A monitor hired by the sponsor(Amsterdam UMC, location AMC)

-National and international supervisory authorities. They keep your information secret. We ask you to give permission for this access. The Health and Youth Care Inspectorate (IGJ) can access your data without your permission.

How long will your data be stored?

We keep your records stored in the hospital and at the sponsor for 15 years.

Can we use your data for other research?

After this study, your data may also be important for other scientific research in the field of prevention of preterm birth in twin pregnancies. To this end, your data will be stored in the hospital for 15 years. You can indicate on the consent form whether you agree with this. If you do not agree to this, you can still participate in the current study. You will receive the same care as any other pregnant women in your situation.

Can you withdraw your consent for the use of your personal data?

For peer review only - http://bmjopen.bmj.com/site/about/guidelines.xhtml

You can withdraw your consent to the use of your personal data and that of your child at any time. This applies to the use of your data for this current study and also to storage and use for future research. Please note: when you withdraw your consent, the data collected up to the time you withdraw your consent may still be used in the research.

More information about your rights when processing data

-Would you like to know more about your rights in the processing of personal data? Then please visit the following website: www.autoriteitpersoonsgegevens.nl.

-Do you have questions about your rights? Or do you have a complaint about the processing of your personal data? Please contact the person responsible for processing your personal data. For this study: [Name of institute] See Appendix A for the contact details and website.

-If you have questions or complaints about the processing of your personal data, we advise you to first contact the research team. You can also contact the Data Protection Officer of [the instite],Amsterdam UMC, or the Dutch Data Protection Authority.

Where can you find additional information on the study?

The following website provides more information about the study: https://www.zorgevaluatienederland.nl. You can find the study by searching for TWIN Cerclage.

10. Do you have an insurance during the study

Insurance has been provided for everyone who participates in this study. The insurance pays for damage caused by the research. But not for all damages. Appendix B contains more information about the insurance and the exceptions. It also tells you who you can report damage to.

11. We will inform your general practitioner and/or treating specialist.

The researcher will send your treating specialist a letter or e-mail to let him/her know that you are participating in this study. We will also inform your general practitioner if you get a cerclage.

12. You have any questions?

You can ask questions about the study to the investigators of this study. Would you like advice from an independent expert? If so, contact details can be found in appendix A. If you have any complaints about the study, you can discuss this with the investigator or your treating specialist. If you prefer not to do so, you may contact the complaints' officer/committee at your hospital. All the relevant details can be found in Appendix A.

13.How do you give consent for the study?

You should first think carefully about participating in this study. Afterwards, you tell the researcher whether you understand the information and whether you want to participate or not. If you would like to participate, please complete the consent form that you can find with this information sheet. You and the researcher will both receive a signed version of this consent form.

Thank you for your attention.

14.Appendices to this information

A.Contact details [each participating hospital needs to change this section]

B.Insurance information

For peer review only - http://bmjopen.bmj.com/site/about/guidelines.xhtml

C.Informed Consent Form

D.Informed consent form for the collection, use and store of data from your pregnancy, your delivery and details of your children

For peer review only - http://bmjopen.bmj.com/site/about/guidelines.xhtml

Appendix A: Contact details for (name participating center)

(Researcher): [for principal investigator of centre: name, contact details and availability]

<if applicable>
[Research nurse/research doctor/nurse specialist]

Independent expert: If you have any doubts about participation, you can consult an independent expert who is not involved in the study herself, but knows a lot about this study. Also if you have questions before or during the study that you would rather not ask your doctor, you can contact the independent expert: Dr. A. Timmermans. She can be reached by telephone via the office of the women's clinic: 020-5663754 on working days during office hours

Complaints:
[service or person with contact details and accessibility]

The Data Protection Officer::
Name of institute: ……………………..
Data protection officier from the sponsor: privacy@amsterdamumc.nl.

For additional information on your rights with respect to the use of your personal data:
Name of institute: local PI
On behalf of the sponsor: Prof. dr. M.A. Oudijk twinc@amsterdamumc.nl

For peer review only - http://bmjopen.bmj.com/site/about/guidelines.xhtml

Appendix B: Insurance information

Insurance has been taken out by the AMC for everyone participating in this study. The insurance covers damage due to participation in the study. This applies to damage manifesting during the study or within 4 years of the end of your participation in the study. You must notify the insurance company about the damage within those 4 years.

In the event of damage please contact the insurance company [or claims adjustor] directly.

The insurance company for the study is:
Name: Centramed B.A.
Address: Postbus 7374
2701 AJ Zoetermeer
Telephone number: 070 301 70 70
E-mail address: info@centramed.nl
Insurance number: 624.528.303

In case of damage, the subject is requested to contact Prof. Dr. M.A. Oudijk, 020-5663754.

The insurance offers a cover of €650,000 per study subject and €5,000,000 for the entire study. For all studies from the same sponsor, the maximum amount of coverage is. €7,500,000 annually.

The insurance policy does **not** cover the following damage:
-Damage as a result of a risk that you were informed about in the written information. This does not apply if the risk occurs in a more severe form than envisaged, or if the risk was very unlikely to occur;
-Damage to your health that would also have occurred if you had not participated in the study;
-Damage resulting from not or not entirely following directions or instructions;
-Damage to descendants as a result of a negative effect of the study on you or your descendants (unless the study specifically involves an existing pregnancy or birth, or embryos for the purpose of creating a pregnancy);
-Damage as a result of an existing treatment method or of research into existing methods of treatment.
- These provisions are set out in the 'Besluit verplichte verzekering bij medisch-wetenschappelijk onderzoek met mensen 2015''. This decision can be found in the government's Wettenbank (https://wetten.overheid.nl).

For peer review only - http://bmjopen.bmj.com/site/about/guidelines.xhtml

Appendix C: Informed consent form TWIN Cerclage studie

Belonging to TWIN Cerclage: The effectiveness of Cerclage for the reduction of extreme preterm birth and perinatal mortality in Twin pregnancies with a short cervix.

- I was able to read the information letter. I was also able to ask questions. My questions have been answered to my satisfaction. I had enough time to decide whether to participate.
- I know that participation is voluntary. I know that I may decide at any time not to participate after all or to withdraw from the study. I do not need to give a reason for this.
- I give the researcher permission to let my treating specialist know that I am participating in this study and your general practitioner if you have are classified to the cerclage group.
- I give consent that the researcher can ask additional information from my midwife, gynecologist general practitioner or specialist about my pregnancy, health and medical history.
- I consent to the collection and use of my data, including ethnicity and education level, and my child (ren)'s data in the manner and for the purposes set out in the information letter.
- I know that some people may have access to all my data to verify the study. These people are listed in this information sheet. I consent to the inspection by them.
- I give permission to keep my data and those of my child (ren) for 15 years after the end of this research.
- I give permission to forward my contact details (address and telephone number) to the research team at Amsterdam UMC so that they can approach me for possible follow-up research and sending the questionnaires

Would you please fill out yes or no in the following table?

I give permission to keep my data to use it for other research, as stated in the information letter.	Yes ☐	No☐
I give permission to be asked if I want to participate in a follow-up study after this study.	Yes ☐	No ☐

- I want to take part in this study.

My name is (subject): ....
My e-mail address:
My telephone number:

Signature: ....

Date: __/__/__

I declare that I have fully informed this subject about this study. If information comes to light during the course of the study that could affect the study subject's consent, I will inform him/her of this in a timely fashion. The study subject will receive the full information sheet, together with a signed copy of the consent form.

Investigator name (or their representative): ....
Signature:

Date: __/__/__

For peer review only - http://bmjopen.bmj.com/site/about/guidelines.xhtml

Appendix D. Statement of consent for the collection, use and storage of data about the pregnancy, childbirth and data of your children

If you decide not to participate in this study, we would like to ask for your consent to the collection, use and retention of data from your pregnancy and childbirth in order to gain a better understanding of threatened preterm birth in twin pregnancies. If you decide that we are allowed to collect your data from your pregnancy and childbirth, we would like to ask you to sign the consent statement.

- I was also able to ask questions. My questions have been answered to my satisfaction. I had enough time to decide whether to participate.
- I have the right to withdraw my consent at any time without having to give a reason.
- I give the researcher permission to request information about my pregnancy, delivery and children from my GP/specialist(s).
- I give permission to keep my data and those of my children for a period of 15 years after the end of the research.
- I give permission for the collection and use of my data, pregnancy and delivery and of my children for answering the research question in this study.
- I give permission to forward my contact details (address and telephone number) to the research team at Amsterdam UMC, so that they can approach me for possible follow-up research and sending the questionnaires.

I give permission to be asked if I want to participate in a follow-up study after this study.	Yes ☐	No ☐

-

My name is (subject): ....
E-mail address:
Telephone number:

Signature: ....

Date: __/__/__

I declare that I have fully informed this subject about this study. If information comes to light during the course of the study that could affect the study subject's consent, I will inform him/her of this in a timely fashion.

For peer review only - http://bmjopen.bmj.com/site/about/guidelines.xhtml

SPIRIT

STANDARD PROTOCOL ITEMS: RECOMMENDATIONS FOR INTERVENTIONAL TRIALS

SPIRIT 2013 Checklist: Recommended items to address in a clinical trial protocol and related documents*

Section/item	Item No	Description
**Administrative information**		
Title	1	Descriptive title identifying the study design, population, interventions, and, if applicable, trial acronym – **Page 1, Line 1-3**
Trial registration	2a	Trial identifier and registry name. If not yet registered, name of intended registry – **Page 3**
	2b	All items from the World Health Organization Trial Registration Data Set **– Page 1-4 and 14**
Protocol version	3	Date and version identifier – **At the bottom of every page**
Funding	4	Sources and types of financial, material, and other support – **Page 14**
Roles and responsibilities	5a	Names, affiliations, and roles of protocol contributors – **Page 1,2,14**
	5b	Name and contact information for the trial sponsor – **Page 3**
	5c	Role of study sponsor and funders, if any, in study design; collection, management, analysis, and interpretation of data; writing of the report; and the decision to submit the report for publication, including whether they will have ultimate authority over any of these activities – **Page 3**
	5d	Composition, roles, and responsibilities of the coordinating centre, steering committee, endpoint adjudication committee, data management team, and other individuals or groups overseeing the trial, if applicable (see Item 21a for data monitoring committee) – **Page 3, 8**
**Introduction**		
Background and rationale	6a	Description of research question and justification for undertaking the trial, including summary of relevant studies (published and unpublished) examining benefits and harms for each intervention – **Page 5, Line 139-184**
	6b	Explanation for choice of comparators – **Page 5, Line 153-184**
Objectives	7	Specific objectives or hypotheses – **Page 6, Line 185-187**

For peer review only - http://bmjopen.bmj.com/site/about/guidelines.xhtml

Trial design	8	Description of trial design including type of trial (eg, parallel group, crossover, factorial, single group), allocation ratio, and framework (eg, superiority, equivalence, noninferiority, exploratory) **– Page 6, Line 189-192**

Methods: Participants, interventions, and outcomes

Study setting	9	Description of study settings (eg, community clinic, academic hospital) and list of countries where data will be collected. Reference to where list of study sites can be obtained **– Page 6, Line 189-192**
Eligibility criteria	10	Inclusion and exclusion criteria for participants. If applicable, eligibility criteria for study centres and individuals who will perform the interventions (eg, surgeons, psychotherapists) **– Page 6, Line 193-210**
Interventions	11a	Interventions for each group with sufficient detail to allow replication, including how and when they will be administered – **Page 7, Line 230-235**
	11b	Criteria for discontinuing or modifying allocated interventions for a given trial participant (eg, drug dose change in response to harms, participant request, or improving/worsening disease) – **Page 9, Line 296-300**
	11c	Strategies to improve adherence to intervention protocols, and any procedures for monitoring adherence (eg, drug tablet return, laboratory tests) – **Page 7, Line 236-260**
	11d	Relevant concomitant care and interventions that are permitted or prohibited during the trial – **Page 7, Line 226-250**
Outcomes	12	Primary, secondary, and other outcomes, including the specific measurement variable (eg, systolic blood pressure), analysis metric (eg, change from baseline, final value, time to event), method of aggregation (eg, median, proportion), and time point for each outcome. Explanation of the clinical relevance of chosen efficacy and harm outcomes is strongly recommended – **Page 7, 8**
Participant timeline	13	Time schedule of enrolment, interventions (including any run-ins and washouts), assessments, and visits for participants. A schematic diagram is highly recommended (see Figure) – **Page 7, Line 226-250**
Sample size	14	Estimated number of participants needed to achieve study objectives and how it was determined, including clinical and statistical assumptions supporting any sample size calculations – **Page 6, Line 211-228**
Recruitment	15	Strategies for achieving adequate participant enrolment to reach target sample size – **Page 7, Line 226-250 and Page 13, Line 455-465**

Methods: Assignment of interventions (for controlled trials)

Allocation:

Sequence generation	16a	Method of generating the allocation sequence (eg, computer-generated random numbers), and list of any factors for stratification. To reduce predictability of a random sequence, details of any planned restriction (eg, blocking) should be provided in a separate document that is unavailable to those who enrol participants or assign interventions – **Page 8, Line 291-295**
Allocation concealment mechanism	16b	Mechanism of implementing the allocation sequence (eg, central telephone; sequentially numbered, opaque, sealed envelopes), describing any steps to conceal the sequence until interventions are assigned **- Page 8, Line 291-295**
Implementation	16c	Who will generate the allocation sequence, who will enrol participants, and who will assign participants to interventions **- Page 8, Line 291-295**
Blinding (masking)	17a	Who will be blinded after assignment to interventions (eg, trial participants, care providers, outcome assessors, data analysts), and how - **Page 8, Line 291-295**
	17b	If blinded, circumstances under which unblinding is permissible, and procedure for revealing a participant's allocated intervention during the trial – **Not applicable**

Methods: Data collection, management, and analysis

Data collection methods	18a	Plans for assessment and collection of outcome, baseline, and other trial data, including any related processes to promote data quality (eg, duplicate measurements, training of assessors) and a description of study instruments (eg, questionnaires, laboratory tests) along with their reliability and validity, if known. Reference to where data collection forms can be found, if not in the protocol – **Page 8, Line 262-290**
	18b	Plans to promote participant retention and complete follow-up, including list of any outcome data to be collected for participants who discontinue or deviate from intervention protocols - **Page 9, Line 301-303**
Data management	19	Plans for data entry, coding, security, and storage, including any related processes to promote data quality (eg, double data entry; range checks for data values). Reference to where details of data management procedures can be found, if not in the protocol – **Page 13, Line 471-478**
Statistical methods	20a	Statistical methods for analysing primary and secondary outcomes. Reference to where other details of the statistical analysis plan can be found, if not in the protocol – **Page 11, Line 375-401**

For peer review only - http://bmjopen.bmj.com/site/about/guidelines.xhtml

	20b	Methods for any additional analyses (eg, subgroup and adjusted analyses) – **Page 11,Line 402-406**
	20c	Definition of analysis population relating to protocol non-adherence (eg, as randomised analysis), and any statistical methods to handle missing data (eg, multiple imputation) – **Page 9, Line 296-303**
**Methods: Monitoring**		
Data monitoring	21a	Composition of data monitoring committee (DMC); summary of its role and reporting structure; statement of whether it is independent from the sponsor and competing interests; and reference to where further details about its charter can be found, if not in the protocol. Alternatively, an explanation of why a DMC is not needed – **Page 10, Line 357-374**
	21b	Description of any interim analyses and stopping guidelines, including who will have access to these interim results and make the final decision to terminate the trial - **Page 10, Line 357-374**
Harms	22	Plans for collecting, assessing, reporting, and managing solicited and spontaneously reported adverse events and other unintended effects of trial interventions or trial conduct - **Page 9, Line 304-328**
Auditing	23	Frequency and procedures for auditing trial conduct, if any, and whether the process will be independent from investigators and the sponsor - **Page 10, Line 357-374**
**Ethics and dissemination**		
Research ethics approval	24	Plans for seeking research ethics committee/institutional review board (REC/IRB) approval – **Page 13, Line 446-454**
Protocol amendments	25	Plans for communicating important protocol modifications (eg, changes to eligibility criteria, outcomes, analyses) to relevant parties (eg, investigators, REC/IRBs, trial participants, trial registries, journals, regulators) **Page 13, Line 446-454**
Consent or assent	26a	Who will obtain informed consent or assent from potential trial participants or authorised surrogates, and how (see Item 32) **Page 13, Line 455-465**
	26b	Additional consent provisions for collection and use of participant data and biological specimens in ancillary studies, if applicable **Page 13, Line 466-469**
Confidentiality	27	How personal information about potential and enrolled participants will be collected, shared, and maintained in order to protect confidentiality before, during, and after the trial **Page 13, Line 446-469**
Declaration of interests	28	Financial and other competing interests for principal investigators for the overall trial and each study site - **Page 3**

For peer review only - http://bmjopen.bmj.com/site/about/guidelines.xhtml

Access to data	29	Statement of who will have access to the final trial dataset, and disclosure of contractual agreements that limit such access for investigators **Page 13, Line 471-478**
Ancillary and post-trial care	30	Provisions, if any, for ancillary and post-trial care, and for compensation to those who suffer harm from trial participation **Not applicable**
Dissemination policy	31a	Plans for investigators and sponsor to communicate trial results to participants, healthcare professionals, the public, and other relevant groups (eg, via publication, reporting in results databases, or other data sharing arrangements), including any publication restrictions **Page 13, Line 453-454**
	31b	Authorship eligibility guidelines and any intended use of professional writers **Page 3**
	31c	Plans, if any, for granting public access to the full protocol, participant-level dataset, and statistical code **Not applicable**
**Appendices**		
Informed consent materials	32	Model consent form and other related documentation given to participants and authorised surrogates – **Supplementary file 1**
Biological specimens	33	Plans for collection, laboratory evaluation, and storage of biological specimens for genetic or molecular analysis in the current trial and for future use in ancillary studies, if applicable **Not applicable**

*It is strongly recommended that this checklist be read in conjunction with the SPIRIT 2013 Explanation & Elaboration for important clarification on the items. Amendments to the protocol should be tracked and dated. The SPIRIT checklist is copyrighted by the SPIRIT Group under the Creative Commons "Attribution-NonCommercial-NoDerivs 3.0 Unported" license.

For peer review only

BMJ Open

Study protocol for two randomized controlled trials evaluating the effect of cerclage in the reduction of extreme preterm birth and perinatal mortality in twin pregnancies with a short cervix or dilatation: the TWIN Cerclage studies

Journal:	*BMJ Open*
Manuscript ID	bmjopen-2023-081561.R2
Article Type:	Protocol
Date Submitted by the Author:	02-Apr-2024

Complete List of Authors:	van Gils, Lissa; Amsterdam UMC Locatie AMC, Department of Obstetrics and Gynaecology Amsterdam<br>de Boer, Marjon; Amsterdam UMC Locatie VUmc, Department of Obstetrics and Gynaecology<br>Bosmans, Judith; Amsterdam UMC Location AMC, Department of Health Sciences<br>Duijnhoven, Ruben; Amsterdam UMC Location AMC, Obstetrics and Gynaecology<br>Schoenmakers, Sam ; Erasmus Medical Center, Department of Obstetrics and Gynaecology<br>Derks, J. ; University Medical Centre Utrecht, Obstetrics<br>Prins, Jelmer; University Medical Center Groningen, Department of Obstetrics and Gynaecology<br>Al-Nasiry, Salwan; Maastricht UMC+, Department of Obstetrics and Gynaecology<br>Lutke Holzik, Margo; Leiden Universitair Medisch Centrum, Department of Obstetrics and Gynecology<br>Lopriore, Enrico; Leiden University Medical Center, Department of neonatology and fetal medicine<br>van Drongelen, J. ; Radboud Universiteit, Department of Obstetrics and Gynecology<br>Knol, H. ; Isala Zwolle, Department of Obstetrics and Gynaecology<br>van Laar, J.; Máxima Medical Center, Department of Obstetrics & Gynaecology<br>Jacquemyn, Y.; University Hospital Antwerp<br>van Holsbeke, Caroline; AZ Sint-Lucas Brugge<br>Dehaene, Isabelle; University Hospital Ghent,<br>Lewi, Liesbeth; KU Leuven University Hospitals Leuven, Fetal Medicine Unit<br>van der Merwe, Hannes; KU Leuven University Hospitals Leuven<br>Gyselaers, Wilfried; Hospital Oost-Limburg, Department of Obstetrics and Gynecology<br>Obermann-Borst, Sylvia; Care4Neo, Neonatal patient and parent advocacy organization.<br>Holthuis, Mayella; Patiëntenorganisaties Extreme Vroeggeboorte<br>Mol, Ben; Monash Medical School, OB/GYN<br>Pajkrt, Eva; Amsterdam UMC Location AMC, Obstetrics and Gynaecology<br>Oudijk, Martijn; AMC, Obstetrics

**Primary Subject Heading**:	Obstetrics and gynaecology
Secondary Subject Heading:	Obstetrics and gynaecology
Keywords:	Fetal medicine < OBSTETRICS, Maternal medicine < OBSTETRICS, OBSTETRICS

SCHOLARONE™
Manuscripts

Study protocol for two randomized controlled trials evaluating the effect of cerclage in the reduction of extreme preterm birth and perinatal mortality in twin pregnancies with a short cervix or dilatation: the TWIN Cerclage studies

Authors

Drs. Lissa van Gils [1]

Dr. Marjon A. de Boer [1]

Prof. Dr. J. Bosmans [2]

Dr. Ruben Duijnhoven[1]

Dr. Sam Schoenmakers [3]

Dr. Jan B. Derks [4]

Dr. Jelmer R. Prins [5]

Dr. Salwan Al-Nasiry [6]

Dr. Margo Lutke Holzik [7]

Dr. Enrico Lopriore [8]

Dr. Joris van Drongelen [9]

Dr. Marieke H. Knol [10]

Dr. Judith O.E.H. van Laar [11]

Prof. Dr. Yves Jacquemyn [12]

Dr. Caroline van Holsbeke [13]

Dr. Isabelle Dehaene [14]

Prof. Dr. Liesbeth Lewi [15]

Dr. Hannes van der Merwe [15]

Dr. Wilfried Gyselaers [16]

Dr. Sylvia A. Obermann-Borst [17]

Mayella Holthuis [18]

Prof. Dr. Ben W. Mol [19]

Prof. Dr. Eva Pajkrt [1]

Prof Dr. Martijn A. Oudijk [1]

For peer review only

For peer review only - http://bmjopen.bmj.com/site/about/guidelines.xhtml

Affiliations

[1] Department of Obstetrics and Gynaecology, Amsterdam University Medical Center, De Boelelaan 1118, 1081 HZ Amsterdam, Netherlands

[2] Department of Health Sciences and the EMGO Institute for Health and Care Research, Faculty of Earth and Life Sciences, Vrije Universiteit Amsterdam, De Boelelaan 1085, 1081 HV Amsterdam, Netherlands

[3] Department of Obstetrics and Gynaecology, Erasmus Medical Center, ‘s- Gravendijkwal 230, 3015 CE Rotterdam, Netherlands

[4] Department of Obstetrics and Gynaecology, Birth Centre Wilhelmina Children Hospital, University Medical Center Utrecht, Heidelberglaan 100, 3584 CX Utrecht, Netherlands

[5] Department of Obstetrics and Gynaecology, University Medical Center Groningen, Hanzeplein 1, 9713 GZ Groningen, Netherlands

[6] Department of Obstetrics and Gynaecology, Maastricht University Medical Center, P. Debyelaan 25, 6229 HX Maastricht, Netherlands

[7] Department of Obstetrics and Gynaecology, Leiden University Medical Center, Albinusdreef 2, 2333 ZA Leiden, Netherlands

[8] Department of Neonatology and Fetal Medicine, Leiden University Medical Center, Albinusdreef 2, 2333 ZA Leiden, Netherlands

[9] Radboud University Medical Center, Department of Obstetrics and Gynaecology, Geert Grooteplein-Zuid 10, 6525 GA Nijmegen, Netherlands

[10] Department of Obstetrics and Gynaecology, Isala Zwolle, Zwolle, Netherlands

[11] Department of Obstetrics and Gynaecology, Maxima Medical Center in Veldhoven, De Run 4600, 5504 DB Veldhoven, Netherlands

[12] Department of Obstetrics and Gynaecology, University Hospital, Antwerpen, Belgium

[13] Department of Obstetrics and Gynaecology, AZ Sint-Lucas, Brugge, West-Vlaanderen, Belgium

[14] Department of Obstetrics and Gynaecology, University Hospital Ghent, Corneel Heymanslaan 10, Gent, Belgium

[15] Leuven University Hospitals Leuven, Fetal Medicine Unit Leuven, Flanders, Belgium

[16] Hospital Oost-Limburg, Department of Obstetrics and Gynaecology, Genk, Belgium

[17] Care4Neo, Neonatal Patient and Parent Advocacy Organization, Rotterdam, Netherlands

[18] Patiëntenorganisaties Extreme Vroeggeboorte, Amsterdam, Netherlands

[19] Department of Obstetrics and Gynaecology, Robinson Research Institute, University of Adelaide, 72 King William St, North Adelaide, SA, Australia

For peer review only - http://bmjopen.bmj.com/site/about/guidelines.xhtml

Correspondence to: L. van Gils, Department of Obstetrics and Gynaecology, Amsterdam University Medical Center, De Boelelaan 1118, 1081 HZ Amsterdam, Netherlands
Email: L.vangils1@amsterdamumc.nl or TWINC@Amsterdamumc.nl

Abstract

Introduction: Twin pregnancies have a high risk of extreme preterm birth (PTB) at less than 28 weeks of gestation which is associated with increased risk of neonatal morbidity and mortality. Currently there is a lack of effective treatments for women with a twin pregnancy and a short cervix or cervical dilatation. A possible effective surgical method to reduce extreme PTB in twin pregnancies with an asymptomatic short cervix or dilatation at midpregnancy is the placement of a vaginal cerclage.

Methods and analysis: We designed two multicentre randomized trials spanned across eight hospitals in the Netherlands (sites in other countries may be added at a later date). Women above the age of 16 with a twin pregnancy < 24 weeks of gestation and an asymptomatic short cervix ≤ 25mm or cervical dilatation, will be randomly allocated (1:1) in both trials to vaginal cerclage or standard treatment according to the current Dutch NVOG guideline (no cerclage). Permuted blocks will be used of sizes 2 and 4 to minimize the risk of disbalance. The primary outcome measure is PTB <28 weeks. Analyses will be by intention to treat. The first trial is to demonstrate a risk reduction from 25% to 10% in the short cervix group, for which 194 patients need to be recruited. The second trial is to demonstrate a risk reduction from 80% to 35% in the dilatation group and will need to recruit 44 women. A cost-effectiveness analysis will be performed from a societal perspective.

Ethics and dissemination: This study has been approved by the Research Ethics Committees (REC) in the Netherlands on 3/30/2023. Participants will be required to sign an informed consent form. The results will be presented at conferences and published in a peer-reviewed journal. Participants will be informed about the results.

Trial registration: ClinicalTrials.gov, NCT05968794.

Strengths and limitations of this study

- These are the largest randomized trials comparing the effect of cerclage on PTB in women with a twin pregnancy and a short cervix or dilatation.
- A strength of the study lies in its comprehensive assessment of outcomes, including preterm birth at various gestational age thresholds, as well as a thorough examination of adverse maternal and neonatal outcomes.
- The Intention to perform long term follow-up of children, thus future parents can be counselled on the long-term effects of this intervention, enhances the strength of this study
- The recruitment of patients could be a potential challenge as the incidence of women with a twin pregnancy and a short cervix ≤ 25 mm or dilatation at midpregnancy is low.

Keywords: Preterm birth, twins, cerclage, randomized controlled trial

For peer review only

For peer review only - http://bmjopen.bmj.com/site/about/guidelines.xhtml

Introduction

Twin pregnancies have a high risk on extreme preterm birth (PTB) at less than 28 weeks of gestation which is associated with increased risk of neonatal morbidity and mortality.(1) Worldwide, twins represent 0.5–4.0% of all births, with rates varying between ethnic groups and countries.(2) Compared with singletons, twins are at increased risk for adverse perinatal outcomes.(3) In the Netherlands, 250 (5,2%) women with a twin pregnancy deliver at < 28 weeks per year, resulting in 157 perinatal deaths and 343 surviving neonates of whom a large proportion suffer from long term neurodevelopmental problems associated with extreme preterm birth.(4) An asymptomatic short cervix (≤ 25 mm) or dilatation at midpregnancy in twin pregnancies is associated with high rates of extreme PTB and proven to be the best predictor for extreme PTB.(5, 6)

Effective interventions for twin pregnancies with short cervix are limited. The EPPPIC individual participant data (IPD) meta-analysis on progesterone treatment in multiple pregnancies with a short cervix showed no reduction of PTB, however a recent IPD meta-analysis did show a possible effect of progesterone.(7, 8) The use of a vaginal pessary in women with a twin pregnancy and a short cervix does not seem to result in a reduction of PTB. (9)

A possible surgical method to reduce extreme PTB in twin pregnancies with an asymptomatic short cervix or dilatation at midpregnancy is a vaginal cerclage. An IPD meta-analysis from 2015 assessed its impact in 49 women with a twin pregnancy and a short cervix (< 25 mm) across three randomized controlled trials.(10) No significant differences were present between the groups on outcomes such as PTB or perinatal death. However, a more recent systematic review with a total of 471 women also included six cohort studies that focused on the outcome of PTB at different cut-offs.(11) The pooled results of these cohort studies showed a significant beneficial effect of a cerclage on PTB <32 weeks (Relative risk (RR) 0.68; 95% confidence interval (CI) 0.51-0.92) and <34 weeks (RR 0.73; 95% CI 0.59-0.90). In addition, a cerclage significantly prolonged pregnancy (mean 2.53 weeks (95% CI 1.25-3.81)). No significant difference was seen in the number of PTBs <24 and 28 weeks or perinatal mortality, although the trend was in favour of the cerclage group.

Furthermore, a subgroup analysis by cervical length was carried out, distinguishing between a cervical length between 16 and 24mm and ≤15mm.(11) For the group with a cervical length <15mm, cerclage placement was associated with less PTB <37 weeks (RR 0.86; 95% CI 0.74-0.99), <34 weeks (RR 0.57; 95% CI 0.43-0.75) and <32 weeks (RR 0.61; 95% CI 0.41-0.90) compared to the control group. Cerclage also resulted in a significant pregnancy prolongation by 3.89 weeks (95% CI 2.19-5.59). In the group of women with a cervical length of 16-24mm, no significant differences were seen in the outcomes.

In women with a twin pregnancy and asymptomatic cervical dilatation, the most recent systematic review was published in August 2023 and included one RCT and five retrospective cohort studies.(12) These studies showed that a vaginal cerclage may result in a reduction of PTB at less than 24 weeks (RR 0.32; 95% CI 0.21-0.48), 28 weeks (RR 0.47; 95% CI 0.36-0.62), 32 weeks (RR 0.59; 95% CI 0.50–0.70) and 34 weeks (RR 0.68; 95% CI 0,57–0,80). In addition, women with a cerclage had a reduced risk of perinatal death (RR 0.30; 95% CI 0.16-0.55), 5 minute Apgar score < 7 (RR 0.49; 95% CI 0.27-0.90), birthweight <1500 grams (RR 0.41; 95% CI 0.31-0.55) and admission to the neonatal intensive care unit (NICU) (RR 0.67; 95% CI 0.52-0.88).

For peer review only - http://bmjopen.bmj.com/site/about/guidelines.xhtml

In conclusion, there is a lack of well-designed RCT's on the effect of vaginal cerclage, on PTB and neonatal outcomes in women with a twin pregnancy with a short cervix or asymptomatic dilatation at midpregnancy. Long-term follow-up of children is lacking in general, thus future parents cannot be counselled on the long-term effects of this intervention. The possible effect of a cerclage in cohort studies justifies a well-designed large multicentre randomized trial. We therefore will perform the TWIN Cerclage study.

The objective of the present study is to assess the effectiveness of a vaginal cerclage in women pregnant with a twin pregnancy with a midpregnancy asymptomatic short cervix or dilatation in the prevention of extreme PTB and perinatal mortality compared to standard treatment (no cerclage).

Methods and analysis

Design and setting

We will conduct two multicentre randomized controlled trials within the Dutch Consortium for Healthcare Evaluation and Research in Obstetrics and Gynaecology (eight hospitals). Sites in other countries may be added at a later date.

Participants and eligibility criteria

RCT I: Women (> 16 years of age) with a twin pregnancy with a short cervix ≤ 25 mm below 24 weeks of pregnancy are eligible for this trial.

RCT II: Women (> 16 years of age) with a twin pregnancy with dilatation on transvaginal ultrasound or physical examination below 24 weeks of pregnancy are eligible for this trial.

Women who meet any of the following criteria will be excluded from participation in these trials:

- Women with a mono-amniotic twin pregnancy
- Women with twin pregnancy in which one or both children are diagnosed with a major structural, congenital or chromosomal abnormality (at time of inclusion) that is likely to influence the composite adverse neonatal outcome.
- Women with signs of clinical intra-uterine infection, defined by the presence of fever ≥ 38 degrees Celsius.
- Women with overt symptoms of preterm labour at time of measurement of short cervix or dilatation (regular contractions, PPROM, recurrent blood loss).
- Women with a placenta previa, defined as a placenta position covering the internal ostium of the cervix.
- Women who do not master the Dutch or English language and are therefore not able to give written consent.

Sample size calculation

In women with a twin pregnancy and an asymptomatic short cervix, the sample size was calculated based on a meta-analysis published in 2019.(11) In this study, the incidence of preterm birth before 28 weeks was lower in the cerclage group (13.8%) compared to the expected management group (23.0%) (RR 0.64; CI 0.40-1.02).

For peer review only - http://bmjopen.bmj.com/site/about/guidelines.xhtml

• Stratum I Short cervix group: To demonstrate a risk reduction from 25% to 10%, 97 patients need to be recruited per arm (194 in total). Sample size for this stratum was calculated using a Z-test with unpooled variance, a two-sided alpha of 0.05 and a target power of 80%.

In women with a twin pregnancy and asymptomatic cervical dilatation, the sample size was calculated based on a systematic review published in December 2021, which included 1 RCT and 4 retrospective cohort studies.(13) In this study, the incidence of preterm birth before 28 weeks was significantly lower in the cerclage group (37.2%) compared to the expected management group (76.9%) (RR 0.44; CI 0.33-0.58).

• Stratum II Dilatation group: To demonstrate a risk reduction from 80% to 35%, 22 patients need to be recruited per arm (44 in total). The sample size for this stratum was calculated using Fisher's exact test, with a two-sided alpha of 0.05 and a target power of 80%. Numbers are not corrected for loss of follow-up, as it is highly unlikely to have loss to follow-up in these high risk pregnancies.

Treatment

The investigated intervention is a vaginal cerclage. Potential complications of a cerclage are infection, premature rupture of membranes, cervical laceration or bleeding and anaesthesia-related complications, occurring in approximately 0.3-2.5 %.(14)

The comparator will be standard treatment according to the current guideline in the Netherlands from 2018, which is to not perform or offer an intervention such as vaginal cerclage.(15)

Study procedures

Measurement of cervical length in twin pregnancies will be performed as part of the standard 20-week ultrasound scan in all hospitals in the Netherlands. In case of a short cervical length or dilatation patients will be seen at the nearest participating centre, where women will be counselled for participation in the study. Inclusion is possible below 24 weeks of gestation, and if randomized to the surgical treatment, the cerclage needs to be placed before 24^{+0} weeks of gestation.

After women give written consent to participate in the trials, patients will be randomized for two treatment options: 1. surgical treatment with cerclage and additional standard care according to Dutch guidelines or 2. Standard care according to Dutch guidelines (no cerclage). Randomization will be done using a web-based interface for each of the two trials. For the trial recruiting women with dilatation, random permuted blocks will be used of sizes 2 and 4 to minimize the risk of disbalance. Participants and investigators will not be blinded for the intervention.

In addition, participants are treated according to the local protocol in the participating clinics i.e. the use of progesterone during pregnancy and other interventions such as tocolysis and corticosteroids in case of a threatened PTB. Follow up of the pregnancy and delivery will be in the referral centre until delivery. The cerclage will be removed according to protocol between 36-37 weeks of gestation, or in case of labour, whatever comes first. Delivery will take place by either spontaneous onset of labour, or induction of labour or elective caesarean section according to national guidelines for twin pregnancies.

The referral hospitals will be contacted for data collection of outcomes as mentioned above by the studies' research midwifes or nurses. Primary and secondary outcome measures will be obtained from clinical patient data. Outcomes such as PTB will be assessed at birth, additional outcomes will

For peer review only - http://bmjopen.bmj.com/site/about/guidelines.xhtml

be assessed at the time of discharge of the child from hospital to home address and at 3 months after discharge.

Outcome measures

Main study parameter/endpoint

The primary outcome will be extreme preterm birth < 28 weeks.

Secondary study parameters/endpoints

Secondary outcomes include:

- A composite for adverse neonatal outcome (including bronchopulmonary dysplasia, periventricular leucomalacia > grade 1, intraventricular hemorrhage > grade 2, necrotizing enterocolitis > stage 2, retinopathy of prematurity > stage 2, proven sepsis and perinatal death)
- All components of the composite outcome will also be assessed separately.
- Preterm birth (indicated and spontaneous) at less than 24, 32, 34 and 37 weeks.
- Premature rupture of membranes (PPROM)
- Gestational age at delivery
- Days on ventilation support
- Days in NICU
- Maternal quality of life
- Maternal outcomes, including sepsis, need for antibiotics, need to remove cerclage in operation room, mode of delivery (% caesarean delivery).
- Healthcare costs

Outcome parameters are in line with the core outcome set for studies on prevention of PTB defined by members of GONet and the Core Outcomes in Women's health (CROWN) initiative.(16)

Parents will be asked consent to approach the parents for long-term follow-up of the children at 2 and 4 years of age. Maternal quality of life will be assessed at inclusion, discharge after birth and 3 months postpartum using the five level version of the EuroQol (EQ-5D-5L).(17) Costs will be measured from a societal perspective using web-based questionnaires based on the iMCQ and iPCQ (Medical and Productivity Cost Questionnaires) at discharge after birth and at 3 months after discharge for the mother and the neonate.

In addition, pregnancy outcome data will also be collected for women opting not to participate in the trials but giving informed consent to collect data on pregnancy outcome from hospital records. Women give permission for this on a voluntary basis.

Randomisation and treatment allocation

Randomization will be done using a web-based interface a member of de project team or a qualified research nurse. Randomization will be stratified for the presence of dilatation at time of recruitment. Allocation will be 1:1 and permuted blocks will be used of sizes 2 and 4 to minimize the risk of disbalance. Participants and investigators will not be blinded for the intervention.

Withdrawal

For peer review only

For peer review only - http://bmjopen.bmj.com/site/about/guidelines.xhtml

Participants can leave the study at any time for any reason if they wish to do so, without any consequences. Patients who withdraw from the study will remain in their treatment group for the intent-to-treat analysis, their data will be used for analysis. The investigator can decide to withdraw a participant from the study for urgent medical reasons.

Follow-up of participants withdrawn from treatment

As the statistical analysis is planned according to intention to treat principle, patients that discontinue the intervention will be analysed in the group that they were allocated to.

Safety reporting

Adverse events (AEs)

An adverse event is defined as an event during or following medicinal treatment or follow up which was not intended to happen and is suspected to be a complication of the intervention performed.

Serious adverse events (SAEs)

Serious Adverse Events (SAEs) will be reported from the first study-related procedure until 3 months after delivery. A serious adverse event is any untoward medical occurrence or effect that:

• results in death (maternal or perinatal)

• is life threatening (at the time of the event);

• requires hospitalisation or prolongation of existing inpatients' hospitalisation

• results in persistent or significant disability or incapacity

• any other important medical event that did not result in any of the outcomes listed above due to medical or surgical intervention but could have been based upon appropriate judgement by the investigator

SAEs must be reported by e-mail to the study project team within 24 hours after the event was reported to the investigator, using the provided SAE report form. This initial report should minimally contain information with respect to the event, associated treatment and patient identification, as described in the detail in the instructions for the SAE report form. If necessary, more detailed information should be provided in a follow-up report within a further 2 business days. SAEs need to be reported until end of study as defined in the protocol. SAEs that result in death or are life threatening should be reported immediately with a maximal 7 days for a preliminary report with another 8 days for completion of the report. All other SAEs will be reported within a period of maximum 15 days after the sponsor initially received knowledge of the serious adverse events.

If required by national laws or regulations or by the procedures of the authorities, the sponsor will ensure that a six-monthly line listing of all reported SAEs is submitted to the Ethics Committee(s).

Events inherent to the study population

Due to the fact that the population is pregnant with a notable risk of premature birth, events inherent to the study population are to be expected and will not be considered as SAE. The study

For peer review only

For peer review only - http://bmjopen.bmj.com/site/about/guidelines.xhtml

project group will report these events by line listing to the Health Authorities, competent authorities, and Research Ethics Committee once a year.

The following events are not considered to be a Serious Adverse Event:

• Maternal hospital admission during pregnancy, delivery or postpartum (with exception of admission to the Intensive Care Unit or Coronary Care Unit, which will be considered as SAE's)

• Maternal hospitalization for a procedure that was planned prior to study participation (i.e. prior to registration or randomization). This should be recorded in the source documents.

• Prolonged maternal hospitalization for a complication of such procedures remains a reportable serious adverse event.

• Neonatal NICU admission

• Neonatal hospital admission other than NICU admission

• Neonatal disease expected in prematurity such as: bronchopulmonary dysplasia, periventricular leucomalacia, intraventricular hemorrhage, necrotizing enterocolitis, retinopathy of prematurity, culture proven sepsis.

Follow-up of (serious) adverse events

Adverse events occurring from the first study-related procedure until 3 months after delivery will be recorded in the CRF. All AEs will be followed until they have abated, or until a stable situation has been reached. Depending on the event, follow up may require additional tests or medical procedures as indicated, and/or referral to the general physician or a medical specialist. All serious adverse events will be followed clinically until they are resolved or until a stable situation has been reached. Depending on the event, follow up may require additional tests or medical procedures as indicated and/or referral to a general physician or a medical specialist. Follow-up information on SAEs should be reported until recovery or until a stable clinical situation has been reached. The final outcome of the SAE should be reported on a final SAE report.

Monitoring and safety

An independent data safety monitoring board (DSMB) will monitor the trials for safety. The DSMB consists of several members with expertise in the relevant fields of obstetrics, neonatology, epidemiology and statistics. Serious adverse events (SAEs) will be collected from the first study-related procedure until 3 months after delivery. SAE's must be reported by e-mail to the study project team within 24 hours after the event was reported to the investigator.

An interim analysis will be conducted for safety (i.e. interim safety review) at least after the outcomes for the first 50 and 100 patients have become available in RCT I (short cervix), or the first 22 patients (half the planned sample size) in RCT II (dilatation group).

Premature termination of the study

For peer review only - http://bmjopen.bmj.com/site/about/guidelines.xhtml

The DSMB can advise to terminate the study prematurely in case of safety concerns. The DSMB can advise to terminate the study prematurely in case the interim analysis shows harm of either one of the interventions (cerclage or no cerclage) or due to external evidence.

An interim analysis will be conducted for safety (i.e. interim safety review) after the outcomes for the first 50 patients have become available for stratum I, and the first 22 patients (half the planned sample size) in stratum II. In addition, after the outcomes of 100 patients have come available from stratum I, the DSMB will meet again for interim safety review.

Statistical analysis

Data analysis will be performed according to the intention-to-treat principle. A supplementary per-protocol analysis is considered if any patients in the standard care arm would have received a cerclage (protocol deviation). Categorical variables will be expressed as a number with the percentage of the total allocation arm. Continuous variables will be presented as mean with standard deviation, as geometric mean with 95% confidence interval (CI) or as median with interquartile range, whenever appropriate.

The primary outcome will be evaluated using the intention-to-treat population. Estimand for both RCT I and RCT II is the relative risk for extreme PTB after cerclage, as compared to the control group not undergoing cerclage. Confidence interval will be a two-sided 95% interval, with statistical inference based on the Chi-squared test for RCT I, and Fisher's exact test for RCT II. Heterogeneity of treatment effect is anticipated to be substantial for the two trials, and a pooled estimate will only be provided would heterogeneity be low. Heterogeneity will be assessed using a forest plot and tests for interaction in a generalized linear model. The two trials may be reported separately if not completed within a reasonable time from each another to allow timely communication of results.

Missing data for the primary outcome will be very low, given that the primary outcome is both an outcome recorded for routine care and there is a short follow-up time. If patients would deliver in other hospitals than the hospital they were recruited from, the data can still be obtained. Only patients who actively revoke consent cannot be analysed for the primary outcome.

The secondary outcome measures be approached similarly to the primary outcome measure. Dichotomous outcomes will be presented as relative risks and absolute with 95% confidence intervals and chi-squared tests or Fisher's exact test. Continuous secondary outcomes, differences between groups will be assessed with the student's t-test if the outcome is normally distributed and with a non-parametric Mann-Whitney U test if skewed. These outcomes will be presented as means with standard deviation, geometric means with 95% CI, or as median with interquartile range, whichever appropriate. A nominal level of 5% will be used for significance. From the perspective of type I error control the two trials are considered as independent tests.

Subgroup analyses

The following subgroup analyses are planned:

- Cervical length ≤ 15 mm and 16-25 mm
- Dichorionic and monochorionic pregnancies
- Multiparous women with a history of PTB and women without a history of PTB
- Nulliparous women and multiparous women with a term birth in their obstetric history

For peer review only

For peer review only - http://bmjopen.bmj.com/site/about/guidelines.xhtml

Economic evaluation

Both a cost-effectiveness analysis (CEA) and a cost-utility analysis (CUA) will be performed from a societal and healthcare perspective according to Dutch guidelines with a time horizon of 3 months after discharge. All statistical analyses will be done according to the intention-to treat principle. Missing cost and effect data will be imputed using multiple imputation according to the MICE algorithm. Rubin's rules will be used to pool the results from the different multiply imputed datasets. Linear regression analyses will be used to estimate cost and effect differences between intervention and control while adjusting for confounders if necessary. Incremental cost-effectiveness ratios (ICERs) will be calculated by dividing the difference in the mean total costs between the treatment groups by the difference in mean effects between the treatment groups. Bias-corrected and accelerated bootstrapping with 5000 replications will be used to estimate 95% confidence intervals around the cost differences and statistical uncertainty surrounding the ICERs. Uncertainty surrounding the ICERs will be graphically presented on cost-effectiveness planes. Cost-effectiveness acceptability curves will also be estimated showing the probability that the intervention is cost-effective in comparison with control for a range of different ceiling ratios thereby showing decision uncertainty.(18)

Budget impact analysis

A budget impact analysis (BIA) will be conducted from the perspective of health-care decision makers according to the Dutch guidelines and the recommendations from Sullivan et al using the Dutch BIA tool.(19) In the BIA, data from the effectiveness and cost-effectiveness analyses regarding the differences in costs and health outcomes will be combined with national prevalence and incidence data to extrapolate the findings to a time horizon of 5 years. The BIA will be conducted from the government perspective (Budget Kader Zorg) and the societal perspective. Actual NZA tariffs will be used to calculate costs. The budget analysis will differentiate between incidental and structural cost (savings), and will take into account budgetary consequences of changes within these cost components. Sensitivity analyses will be performed for subgroups of patients if applicable, providing budget information for relevant subgroups to decision makers. In addition, sensitivity analyses will address the impact of variations of the main assumptions and input parameters for the BIA.

Handling and storage of data and documents

Data will be collected using Castor EDC. Data monitoring will be done by the primary investigator. Data processing will be done coded, as the patient code only available to the local investigators. All data will be stored for 15 years in a secured database on a secured computer, to which only the investigators will have access. All personal data is protected conform the rules of the General Data Protection Regulation. Data on ethnicity and level of education will be collected from the patients' medical file because this is one of the known risk factors for PTB. The participant will give her consent for this on the consent form (Supplementary File 1).

Monitoring and quality assurance

Data monitoring will be performed by a certified clinical research associate of the participating institute or its delegate. The monitor will compare the data entered into the database with the hospital or clinic records (source documents). The nature and location of all source documents will be identified to ensure that all sources of original data required to complete the database are known to the investigational staff and are accessible for verification. Frequency of monitoring will be further discussed with the monitoring party.

For peer review only - http://bmjopen.bmj.com/site/about/guidelines.xhtml

Patient and public involvement

The Dutch consortium collaborates with three Dutch patient associations, Care4Neo (Neonatal patient and parent advocacy organization), the 'Vereniging voor Ouders van Meerlingen (NVOM)' (society of parents of multiples) and the patient organization for extreme PTB. They are project members of the TWINC Cerclage study and involved in the design of the study. A survey was performed during the design of the study among members of the closed Facebook group of both patient associations, to evaluate the interest in participation in the TWIN Cerclage study. The Dutch consortium has a website where it publishes all results of completed studies and publishes the protocols of currently recruiting studies. Participants will be informed about the results.

Ethics and dissemination

The Research Ethics Committee (REC) at the Amsterdam University Medical Centres approved this study (reference number: 2022.0885 - NL82609.018.22). Additional regional approval was obtained for the remaining participating hospitals in the Netherlands. If other study sites are added, ethical approval will be required as relevant. Protocol amendments will be communicated to relevant parties.

Recruitment and consent

Eligible participants will receive the participant information leaflet and will be informed about the study during an outpatient clinic (Supplementary File 1). Because of the need to initiate treatment as soon as possible (<24 weeks of pregnancy) we will call the participant in 1 to 5 days (based upon her gestational age at time of counselling) get the opportunity to ask questions to ask if she wants to participate in the study. If the participant wants to participate, she will be ask to sign the informed consent and ask to send a photo of her signed informed consent. To schedule the cerclage randomization will be performed after receiving a copy of the informed consent. If women are randomized to the cerclage group, they will be ask to bring the original signed informed consent at the day of their admission for the cerclage. If women randomize for standard care, they will be ask to send the original signed informed consent back with the return envelope.

Public disclosure and publication policy

The study results will be published in peer-reviewed, indexed, journals with an open access policy, and the results will be presented at academic conferences. A separate manuscript will be prepared reporting the cost-effectiveness analysis. Authorship eligibility will be in accordance with The International Committee of Medical Journal Editors. Participants will be informed about the results.

Study status

Participants are currently being recruited and enrolled (start date: 16-05-2023). The estimated completion is 01-12-2028.

For peer review only - http://bmjopen.bmj.com/site/about/guidelines.xhtml

Competing interests: None.

Funding: This study is funded by ZonMw (The Netherlands organisation for health research and development), grant number 10390012110017.

Trial sponsor: Amsterdam University Medical Center, De Boelelaan 1118, 1081 HZ Amsterdam, Netherlands. Role of coordinating centre which will perform the analysis and interpreted the data. All members of the TWIN Cerclage project group will participate in writing the report of the final analysis and the decision to submit the report for publication.

Contributors: Drs. Lissa van Gils, Dr. Marjon A. de Boer, Prof. Dr. J. Bosmans, Dr. Ruben Duijnhoven, Dr. Sam Schoenmakers, Dr. Jan B. Derks, Dr. Jelmer R. Prins, Dr. Salwan Al-Nasiry, Dr. Margo Lutke Holzik, Dr. Enrico Lopriore, Dr. Joris van Drongelen, Dr. Marieke H. Knol, Dr. Judith O.E.H. van Laar , Prof. Dr. Yves Jacquemyn, Dr. Caroline van Holsbeke, Dr. Isabelle Dehaene, Prof. Dr. Liesbeth Lewi, Dr. Hannes van der Merwe, Dr. Wilfried Gyselaers, Dr. Sylvia A. Obermann-Borst, Mayella Holthuis, Prof. Dr. Ben W. Mol, Prof. Dr. Eva Pajkrt and Prof Dr. Martijn A. Oudijk were all involved in the conception and design of this study. All authors mentioned in the manuscript are member of the TWIN Cerclage study group. Dr. Sam Schoenmakers, Dr. Jan B. Derks, Dr. Jelmer R. Prins, Dr. Salwan Al-Nasiry, Dr. Margo Lutke Holzik, Dr. Enrico Lopriore, Dr. Joris van Drongelen, Dr. Marieke H. Knol, Dr. Judith O.E.H. van Laar , Prof. Dr. Yves Jacquemyn, Dr. Caroline van Holsbeke, Dr. Isabelle Dehaene, Prof. Dr. Liesbeth Lewi, Dr. Hannes van der Merwe and Dr. Wilfried Gyselaers are local investigators in the participating centres. Dr. Sylvia A. Obermann-Borst and Mayella Holthuis represent two Dutch patient associations (Care4Neo and the patient organization for extreme PTB). Drs. Lissa van Gils and Prof. Dr. Martijn A. Oudijk drafted this manuscript. All other authors reviewed and edited the manuscript. All authors approved the final version of this protocol and agreed to be accountable for all aspects of the work in ensuring that questions related to the accuracy or integrity of any part of the work are appropriately investigated and resolved.

References

1. Schaaf JM, Mol BW, Abu-Hanna A, Ravelli AC. Trends in preterm birth: singleton and multiple pregnancies in the Netherlands, 2000-2007. BJOG. 2011;118(10):1196-204.
2. Deng C, Dai L, Yi L, Li X, Deng K, Mu Y, et al. Temporal trends in the birth rates and perinatal mortality of twins: A population-based study in China. PLoS One. 2019;14(1):e0209962.
3. Russo FM, Pozzi E, Pelizzoni F, Todyrenchuk L, Bernasconi DP, Cozzolino S, Vergani P. Stillbirths in singletons, dichorionic and monochorionic twins: a comparison of risks and causes. Eur J Obstet Gynecol Reprod Biol. 2013;170(1):131-6.
4. data P. Perinatale registratie Nederland. 2019.
5. Honest H, Bachmann LM, Coomarasamy A, Gupta JK, Kleijnen J, Khan KS. Accuracy of cervical transvaginal sonography in predicting preterm birth: a systematic review. Ultrasound Obstet Gynecol. 2003;22(3):305-22.
6. Lim AC, Hegeman MA, Huis In TVMA, Opmeer BC, Bruinse HW, Mol BW. Cervical length measurement for the prediction of preterm birth in multiple pregnancies: a systematic review and bivariate meta-analysis. Ultrasound Obstet Gynecol. 2011;38(1):10-7.

For peer review only - http://bmjopen.bmj.com/site/about/guidelines.xhtml

7. Group E. Evaluating Progestogens for Preventing Preterm birth International Collaborative (EPPPIC): meta-analysis of individual participant data from randomised controlled trials. Lancet. 2021;397(10280):1183-94.

8. Romero R, Conde-Agudelo A, Rehal A, Da Fonseca E, Brizot ML, Rode L, et al. Vaginal progesterone for the prevention of preterm birth and adverse perinatal outcomes in twin gestations with a short cervix: an updated individual patient data meta-analysis. Ultrasound Obstet Gynecol. 2022;59(2):263-6.

9. Hoffman M. Randomized trial of cervical pessary for short cervix in singleton pregnancies. American journal of obstetrics and gynecology. 2023;228(1):S5-S6.

10. Saccone G, Rust O, Althuisius S, Roman A, Berghella V. Cerclage for short cervix in twin pregnancies: systematic review and meta-analysis of randomized trials using individual patient-level data. Acta Obstet Gynecol Scand. 2015;94(4):352-8.

11. Li C, Shen J, Hua K. Cerclage for women with twin pregnancies: a systematic review and metaanalysis. Am J Obstet Gynecol. 2019;220(6):543-57 e1.

12. D'Antonio F, Eltaweel N, Prasad S, Flacco ME, Manzoli L, Khalil A. Cervical cerclage for prevention of preterm birth and adverse perinatal outcome in twin pregnancies with short cervical length or cervical dilatation: A systematic review and meta-analysis. PLoS medicine. 2023;20(8):e1004266.

13. Su J, Li D, Yang Y, Cao Y, Yin Z. Cerclage placement in twin pregnancies with cervical dilation: a systematic review and meta-analysis. J Matern Fetal Neonatal Med. 2022;35(25):9112-8.

14. van Dijk CE, Breuking SH, Jansen S, Limpens J, Kazemier BM, Pajkrt E. Perioperative complications of a transvaginal cervical cerclage in singleton pregnancies: a systematic review and meta-analysis. AMERICAN JOURNAL OF OBSTETRICS AND GYNECOLOGY. 2023;228(5):521-52+.

15. (NVOG) DSfOaGNVvOeG. Preventie-Vroeggeboorte-bij-Meerlingzwangerschappen-def-2018. 2018.

16. van 't Hooft J. A core outcome set for evaluation of interventions to prevent preterm birth: summary for CROWN. BJOG. 2016;123 Suppl 3:107.

17. M MV, K MV, S MAAE, de Wit GA, Prenger R, E AS. Dutch Tariff for the Five-Level Version of EQ-5D. Value Health. 2016;19(4):343-52.

18. Fenwick E, O'Brien BJ, Briggs A. Cost-effectiveness acceptability curves--facts, fallacies and frequently asked questions. Health Econ. 2004;13(5):405-15.

19. Sullivan SD, Mauskopf JA, Augustovski F, Jaime Caro J, Lee KM, Minchin M, et al. Budget impact analysis-principles of good practice: report of the ISPOR 2012 Budget Impact Analysis Good Practice II Task Force. Value Health. 2014;17(1):5-14.

For peer review only

For peer review only - http://bmjopen.bmj.com/site/about/guidelines.xhtml

Patient Information – TWIN cerclage study

The TWIN Cerclage Study: *The effectiveness of Cerclage for the reduction of extreme preterm birth and perinatal mortality in Twin pregnancies with a short cervix.*

Dear Madam,

You are asked to participate in a medical scientific study. Participation is voluntary. Your written consent is required to participate. You are receiving this letter because you are pregnant with twins and are at risk of having a preterm delivery. You can read about the medical study in this information sheet, what it means for you, and about the advantages and disadvantages. It's a lot of information. Would you please read the information and decide if you want to participate? If you would like to participate, please fill out the form found in Appendix C.

Ask your questions

You can make your decision with the information you find in this information letter. In addition, we encourage you to:

- Ask the researcher giving you this information any questions you have.
-Talk to your partner, family or friends about this study.
-Pose your questions to the independent expert, see Appendix A for contact information.
-Read the information at www.rijksoverheid.nl/mensenonderzoek

1. General information

This study has been designed by the Department of Obstetrics and Gynecology of the Amsterdam University Medical Center, location AMC. The study will be conducted by gynecologists, clinicians or midwives. A total of 194 pregnant women with a short cervix and 44 women with dilatation of the cervix are required for this study. This study is approved by the Medical Ethics Committee (METC) Academic Medical Centre.

2. Purpose of the study

The purpose of this study is to investigate the effectiveness of a vaginal cerclage in women with a twin pregnancy and a midpregnancy short cervix compared to standard treatment (no cerclage) in the prevention of extreme preterm birth < 28 weeks of gestational age. The effectiveness of a vaginal cerclage will be compared with standard care according to the (inter)national guidelines, no cerclage.

3. Background of the study

Twin pregnancies have a high risk of extreme preterm birth (PTB) at less than 28 weeks of gestation which is associated with increased risk of neonatal morbidity and mortality. In the Netherlands, per year 250 women with a twin pregnancy deliver at < 28 weeks, resulting in 157 perinatal deaths. One of the risk factors for extreme preterm birth is a short cervix. If a short cervix is ≤ 25mm at the 20-week ultrasound, there is a high risk of an extremely premature birth. A possible effective surgical method to reduce extreme PTB in twin pregnancies with a short cervix or asymptomatic dilatation at midpregnancy is a minor operative procedure, the placement of a vaginal cerclage

For peer review only - http://bmjopen.bmj.com/site/about/guidelines.xhtml

4. What does participation mean?

How long will the study last?

Are you participating in the study? Then the study will run until three months after you gave birth. A few years after the study is finished, we may want to contact you to inquire through a questionnaire how you and your child are doing. At that time, you can decide whether you wish to answer our questions. No additional diagnostic tests are performed on you or your baby.

Are you suitable for participation in the study?

Women above the age of 16 years with a twin pregnancy and a short cervix or cervical dilatation below the 24th week of pregnancy can participate. It may happen that you are healthy, but that you are not suitable to participate. The researcher will tell you more about this.

The treatment

If you consent to participate in the study you will be randomly assigned to either the group receiving standard care (no cerclage) or a cerclage. A vaginal cerclage is a minor surgical procedure in which an unabsorbable suture is placed around/through the cervix to close the cervical canal and to increase its firmness, in order to reduce cervical insufficiency and this pathway to preterm birth. The chance to end up in either one of the groups is equal.

Examinations and measurements

During the standard 20-week ultrasound (SEO), the cervix is measured in your own hospital. If the cervix is short (<25mm), you will be referred to a hospital that participates in this study. The participating hospital can decide to measure the cervical length again and if indicated perform physical examination to detect possible cervical dilatation. If you have drawn for vaginal cerclage group, the cerclage will be placed under anesthesia or epidural. You will be admitted to the hospital for one day.

During the study, data about yourself, the pregnancy, the delivery and your children will be collected. For example, data such as your date of birth, ethnicity and level of education. Collecting data on ethnicity is important because people of different ethnicities may have different cervical lengths and may also respond differently to treatment.

Furthermore, we ask you to complete three online questionnaires: at the start of the treatment, after discharge from the hospital after the delivery and three months after delivery. Completing the questionnaires will take approximately 30 minutes. The questionnaires are completed online on a safe and private server. We ask your permission for using your email address to send you the questionnaires.

Follow-up: What is different from regular care?

If you have drawn for standard care, the pregnancy will be followed up by your own gynecologist according to the usual care. If you have drawn for a cerclage, the pregnancy will also be followed up by your own gynecologist with the usual check-ups after placement. . In addition to the treatment, no additional checks are necessary and no additional examinations will be performed. The cerclage will be removed at 36-37 weeks of pregnancy or sooner if labor starts before then.

For peer review only - http://bmjopen.bmj.com/site/about/guidelines.xhtml

5. What do we expect from you?

We strive for the research to proceed as planned. Therefore, we will make the following agreements with you:

- You will come to every control visit
- You contact the investigator if:
    - You are hospitalized
    - You suddenly start having problems with your health
    - You no longer want to participate in the study
    - Your phone number, address or email address has changed

6. What side effects, adverse reactions or discomforts may you experience?

A vaginal cerclage is a minor and safe surgical procedure commonly performed in singleton pregnancies with a short cervix or dilatation and a previous preterm birth in all the participating centers, thus there is experience in the participating hospitals. Potential complications of a cerclage are infection, premature rupture of membranes, cervical laceration or bleeding and anesthesia-related complications, occurring in approximately 0.3-2.5 %. In addition, placing a cerclage is a surgical procedure and there are the small general risks of surgery and anesthesia or an epidural.

7. Possible advantages and disadvantages

It is important to weigh the pros and cons before you decide to participate. It is currently unclear whether a vaginal cerclage reduces preterm birth rates in women with a twin pregnancy and a short cervix. We cannot guarantee you will benefit from participating in this study. The study may however be useful for other pregnant women in the future and help in the search for better treatment of premature birth in twin pregnancies

If you do not wish to participate or wish to stop your participation

Your participation in this study is completely voluntary. If you do not wish to participate, you will be treated according to local protocol (no cerclage). If you decide not to participate, we would like to ask for your consent to collect, use and store data from your pregnancy and childbirth in order to gain insight into the treatment of preterm birth in twin pregnancies. If you decide that we are allowed to collect your data from your pregnancy and childbirth, we would like to ask you to sign the consent statement in Appendix D.

8. The end of the study

If there is new information about the study that is of interest to you, the investigator will inform you. You will then be asked whether you want to continue your participation.

Your participation in the study stops when:

- Three months after you gave birth
- You decide to withdraw from the study. You can decide to stop at any time during the study and you can inform the investigator about this. You do not have to explain why you want to discontinue participation.
- If you physician decides it’s better for you to stop-If the investigator, the government or the judging medical ethical committee decide to stop the study

What happens when you discontinue participation?

The researchers will use your data that is collected until the moment you decide to discontinue the study. If you are assigned to the cerclage group, your physician will discuss your wishes regarding the cerclage. Overall, the study is concluded once all the participants have completed the study.

For peer review only - http://bmjopen.bmj.com/site/about/guidelines.xhtml

9. Usage and storage of your data

Are you participating in the study? If so, then you also give us permission to use and store your personal data.

What personal data do we keep?

- Your and your babies' names
- Your and your babies' genders
- Your address
- Your and your babies date of birth
- Your ethnicity
- Your level of education
- Information about your and your babies' health
- Data about your pregnancy and delivery
- (Medical) data that we collect during the study

Why do we collect, use and store your data?

We collect, use and store your and your children's data to answer the research questions and to be able to publish the results.

How do we protect your privacy?

To protect your privacy, your data is encrypted. All your data can only be traced back to you with a key of the code. The key to the code remains securely stored in the local research facility. When processing your data, we only use this code. The data cannot be traced back to you in reports and publications about the study.

Who will have access to my data?

Some people can access all the data at the study location, including the data without a code. This is necessary to check whether the study is being conducted in a good and reliable manner. Persons who have access to your data for review are:

-Members of the committee that monitors the safety of the study

- A monitor hired by the sponsor(Amsterdam UMC, location AMC)

-National and international supervisory authorities. They keep your information secret. We ask you to give permission for this access. The Health and Youth Care Inspectorate (IGJ) can access your data without your permission.

How long will your data be stored?

We keep your records stored in the hospital and at the sponsor for 15 years.

Can we use your data for other research?

After this study, your data may also be important for other scientific research in the field of prevention of preterm birth in twin pregnancies. To this end, your data will be stored in the hospital for 15 years. You can indicate on the consent form whether you agree with this. If you do not agree to this, you can still participate in the current study. You will receive the same care as any other pregnant women in your situation.

Can you withdraw your consent for the use of your personal data?

For peer review only - http://bmjopen.bmj.com/site/about/guidelines.xhtml

You can withdraw your consent to the use of your personal data and that of your child at any time. This applies to the use of your data for this current study and also to storage and use for future research. Please note: when you withdraw your consent, the data collected up to the time you withdraw your consent may still be used in the research.

More information about your rights when processing data

-Would you like to know more about your rights in the processing of personal data? Then please visit the following website: www.autoriteitpersoonsgegevens.nl.

-Do you have questions about your rights? Or do you have a complaint about the processing of your personal data? Please contact the person responsible for processing your personal data. For this study: [Name of institute] See Appendix A for the contact details and website.

-If you have questions or complaints about the processing of your personal data, we advise you to first contact the research team. You can also contact the Data Protection Officer of [the instite],Amsterdam UMC, or the Dutch Data Protection Authority.

Where can you find additional information on the study?

The following website provides more information about the study: https://www.zorgevaluatienederland.nl. You can find the study by searching for TWIN Cerclage.

10. Do you have an insurance during the study

Insurance has been provided for everyone who participates in this study. The insurance pays for damage caused by the research. But not for all damages. Appendix B contains more information about the insurance and the exceptions. It also tells you who you can report damage to.

11. We will inform your general practitioner and/or treating specialist.

The researcher will send your treating specialist a letter or e-mail to let him/her know that you are participating in this study. We will also inform your general practitioner if you get a cerclage.

12. You have any questions?

You can ask questions about the study to the investigators of this study. Would you like advice from an independent expert? If so, contact details can be found in appendix A. If you have any complaints about the study, you can discuss this with the investigator or your treating specialist. If you prefer not to do so, you may contact the complaints' officer/committee at your hospital. All the relevant details can be found in Appendix A.

13.How do you give consent for the study?

You should first think carefully about participating in this study. Afterwards, you tell the researcher whether you understand the information and whether you want to participate or not. If you would like to participate, please complete the consent form that you can find with this information sheet. You and the researcher will both receive a signed version of this consent form.

Thank you for your attention.

14.Appendices to this information

A.Contact details [each participating hospital needs to change this section]

B.Insurance information

For peer review only - http://bmjopen.bmj.com/site/about/guidelines.xhtml

C.Informed Consent Form

D.Informed consent form for the collection, use and store of data from your pregnancy, your delivery and details of your children

For peer review only - http://bmjopen.bmj.com/site/about/guidelines.xhtml

Appendix A: Contact details for (name participating center)

(Researcher): [for principal investigator of centre: name, contact details and availability]

<if applicable>
[Research nurse/research doctor/nurse specialist]

Independent expert: If you have any doubts about participation, you can consult an independent expert who is not involved in the study herself, but knows a lot about this study. Also if you have questions before or during the study that you would rather not ask your doctor, you can contact the independent expert: Dr. A. Timmermans. She can be reached by telephone via the office of the women's clinic: 020-5663754 on working days during office hours

Complaints:
[service or person with contact details and accessibility]

The Data Protection Officer::
Name of institute: ……………………..
Data protection officier from the sponsor: privacy@amsterdamumc.nl.

For additional information on your rights with respect to the use of your personal data:
Name of institute: local PI
On behalf of the sponsor: Prof. dr. M.A. Oudijk twinc@amsterdamumc.nl

For peer review only - http://bmjopen.bmj.com/site/about/guidelines.xhtml

Appendix B: Insurance information

Insurance has been taken out by the AMC for everyone participating in this study. The insurance covers damage due to participation in the study. This applies to damage manifesting during the study or within 4 years of the end of your participation in the study. You must notify the insurance company about the damage within those 4 years.

In the event of damage please contact the insurance company [or claims adjustor] directly.

The insurance company for the study is:
Name: Centramed B.A.
Address: Postbus 7374
2701 AJ Zoetermeer
Telephone number: 070 301 70 70
E-mail address: info@centramed.nl
Insurance number: 624.528.303

In case of damage, the subject is requested to contact Prof. Dr. M.A. Oudijk, 020-5663754.

The insurance offers a cover of €650,000 per study subject and €5,000,000 for the entire study. For all studies from the same sponsor, the maximum amount of coverage is. €7,500,000 annually.

The insurance policy does **not** cover the following damage:
-Damage as a result of a risk that you were informed about in the written information. This does not apply if the risk occurs in a more severe form than envisaged, or if the risk was very unlikely to occur;
-Damage to your health that would also have occurred if you had not participated in the study;
-Damage resulting from not or not entirely following directions or instructions;
-Damage to descendants as a result of a negative effect of the study on you or your descendants (unless the study specifically involves an existing pregnancy or birth, or embryos for the purpose of creating a pregnancy);
-Damage as a result of an existing treatment method or of research into existing methods of treatment.
- These provisions are set out in the 'Besluit verplichte verzekering bij medisch-wetenschappelijk onderzoek met mensen 2015''. This decision can be found in the government's Wettenbank (https://wetten.overheid.nl).

For peer review only - http://bmjopen.bmj.com/site/about/guidelines.xhtml

Appendix C: Informed consent form TWIN Cerclage studie

Belonging to TWIN Cerclage: The effectiveness of Cerclage for the reduction of extreme preterm birth and perinatal mortality in Twin pregnancies with a short cervix.

- I was able to read the information letter. I was also able to ask questions. My questions have been answered to my satisfaction. I had enough time to decide whether to participate.
- I know that participation is voluntary. I know that I may decide at any time not to participate after all or to withdraw from the study. I do not need to give a reason for this.
- I give the researcher permission to let my treating specialist know that I am participating in this study and your general practitioner if you have are classified to the cerclage group.
- I give consent that the researcher can ask additional information from my midwife, gynecologist general practitioner or specialist about my pregnancy, health and medical history.
- I consent to the collection and use of my data, including ethnicity and education level, and my child (ren)'s data in the manner and for the purposes set out in the information letter.
- I know that some people may have access to all my data to verify the study. These people are listed in this information sheet. I consent to the inspection by them.
- I give permission to keep my data and those of my child (ren) for 15 years after the end of this research.
- I give permission to forward my contact details (address and telephone number) to the research team at Amsterdam UMC so that they can approach me for possible follow-up research and sending the questionnaires

Would you please fill out yes or no in the following table?

I give permission to keep my data to use it for other research, as stated in the information letter.	Yes ☐	No☐
I give permission to be asked if I want to participate in a follow-up study after this study.	Yes ☐	No ☐

- I want to take part in this study.

My name is (subject): ....
My e-mail address:
My telephone number:

Signature: ....

Date: __/__/__

I declare that I have fully informed this subject about this study. If information comes to light during the course of the study that could affect the study subject's consent, I will inform him/her of this in a timely fashion. The study subject will receive the full information sheet, together with a signed copy of the consent form.

Investigator name (or their representative): ....
Signature:

Date: __/__/__

For peer review only - http://bmjopen.bmj.com/site/about/guidelines.xhtml

Appendix D. Statement of consent for the collection, use and storage of data about the pregnancy, childbirth and data of your children

If you decide not to participate in this study, we would like to ask for your consent to the collection, use and retention of data from your pregnancy and childbirth in order to gain a better understanding of threatened preterm birth in twin pregnancies. If you decide that we are allowed to collect your data from your pregnancy and childbirth, we would like to ask you to sign the consent statement.

- I was also able to ask questions. My questions have been answered to my satisfaction. I had enough time to decide whether to participate.
- I have the right to withdraw my consent at any time without having to give a reason.
- I give the researcher permission to request information about my pregnancy, delivery and children from my GP/specialist(s).
- I give permission to keep my data and those of my children for a period of 15 years after the end of the research.
- I give permission for the collection and use of my data, pregnancy and delivery and of my children for answering the research question in this study.
- I give permission to forward my contact details (address and telephone number) to the research team at Amsterdam UMC, so that they can approach me for possible follow-up research and sending the questionnaires.

I give permission to be asked if I want to participate in a follow-up study after this study.	Yes ☐	No ☐

-

My name is (subject): ....
E-mail address:
Telephone number:

Signature: ....

Date: __/__/__

I declare that I have fully informed this subject about this study. If information comes to light during the course of the study that could affect the study subject's consent, I will inform him/her of this in a timely fashion.

For peer review only - http://bmjopen.bmj.com/site/about/guidelines.xhtml

SPIRIT

STANDARD PROTOCOL ITEMS: RECOMMENDATIONS FOR INTERVENTIONAL TRIALS

SPIRIT 2013 Checklist: Recommended items to address in a clinical trial protocol and related documents*

Section/item	Item No	Description
**Administrative information**		
Title	1	Descriptive title identifying the study design, population, interventions, and, if applicable, trial acronym – **Page 1, Line 1-3**
Trial registration	2a	Trial identifier and registry name. If not yet registered, name of intended registry – **Page 3**
	2b	All items from the World Health Organization Trial Registration Data Set **– Page 1-4 and 14**
Protocol version	3	Date and version identifier – **At the bottom of every page**
Funding	4	Sources and types of financial, material, and other support – **Page 14**
Roles and responsibilities	5a	Names, affiliations, and roles of protocol contributors – **Page 1,2,14**
	5b	Name and contact information for the trial sponsor – **Page 3**
	5c	Role of study sponsor and funders, if any, in study design; collection, management, analysis, and interpretation of data; writing of the report; and the decision to submit the report for publication, including whether they will have ultimate authority over any of these activities – **Page 3**
	5d	Composition, roles, and responsibilities of the coordinating centre, steering committee, endpoint adjudication committee, data management team, and other individuals or groups overseeing the trial, if applicable (see Item 21a for data monitoring committee) – **Page 3, 8**
**Introduction**		
Background and rationale	6a	Description of research question and justification for undertaking the trial, including summary of relevant studies (published and unpublished) examining benefits and harms for each intervention – **Page 5, Line 139-184**
	6b	Explanation for choice of comparators – **Page 5, Line 153-184**
Objectives	7	Specific objectives or hypotheses – **Page 6, Line 185-187**

For peer review only - http://bmjopen.bmj.com/site/about/guidelines.xhtml

Trial design	8	Description of trial design including type of trial (eg, parallel group, crossover, factorial, single group), allocation ratio, and framework (eg, superiority, equivalence, noninferiority, exploratory) **– Page 6, Line 189-192**

Methods: Participants, interventions, and outcomes

Study setting	9	Description of study settings (eg, community clinic, academic hospital) and list of countries where data will be collected. Reference to where list of study sites can be obtained **– Page 6, Line 189-192**
Eligibility criteria	10	Inclusion and exclusion criteria for participants. If applicable, eligibility criteria for study centres and individuals who will perform the interventions (eg, surgeons, psychotherapists) **– Page 6, Line 193-210**
Interventions	11a	Interventions for each group with sufficient detail to allow replication, including how and when they will be administered – **Page 7, Line 230-235**
	11b	Criteria for discontinuing or modifying allocated interventions for a given trial participant (eg, drug dose change in response to harms, participant request, or improving/worsening disease) – **Page 9, Line 296-300**
	11c	Strategies to improve adherence to intervention protocols, and any procedures for monitoring adherence (eg, drug tablet return, laboratory tests) – **Page 7, Line 236-260**
	11d	Relevant concomitant care and interventions that are permitted or prohibited during the trial – **Page 7, Line 226-250**
Outcomes	12	Primary, secondary, and other outcomes, including the specific measurement variable (eg, systolic blood pressure), analysis metric (eg, change from baseline, final value, time to event), method of aggregation (eg, median, proportion), and time point for each outcome. Explanation of the clinical relevance of chosen efficacy and harm outcomes is strongly recommended – **Page 7, 8**
Participant timeline	13	Time schedule of enrolment, interventions (including any run-ins and washouts), assessments, and visits for participants. A schematic diagram is highly recommended (see Figure) – **Page 7, Line 226-250**
Sample size	14	Estimated number of participants needed to achieve study objectives and how it was determined, including clinical and statistical assumptions supporting any sample size calculations – **Page 6, Line 211-228**
Recruitment	15	Strategies for achieving adequate participant enrolment to reach target sample size – **Page 7, Line 226-250 and Page 13, Line 455-465**

Methods: Assignment of interventions (for controlled trials)

Allocation:

Sequence generation	16a	Method of generating the allocation sequence (eg, computer-generated random numbers), and list of any factors for stratification. To reduce predictability of a random sequence, details of any planned restriction (eg, blocking) should be provided in a separate document that is unavailable to those who enrol participants or assign interventions – **Page 8, Line 291-295**
Allocation concealment mechanism	16b	Mechanism of implementing the allocation sequence (eg, central telephone; sequentially numbered, opaque, sealed envelopes), describing any steps to conceal the sequence until interventions are assigned **- Page 8, Line 291-295**
Implementation	16c	Who will generate the allocation sequence, who will enrol participants, and who will assign participants to interventions **- Page 8, Line 291-295**
Blinding (masking)	17a	Who will be blinded after assignment to interventions (eg, trial participants, care providers, outcome assessors, data analysts), and how - **Page 8, Line 291-295**
	17b	If blinded, circumstances under which unblinding is permissible, and procedure for revealing a participant's allocated intervention during the trial – **Not applicable**

Methods: Data collection, management, and analysis

Data collection methods	18a	Plans for assessment and collection of outcome, baseline, and other trial data, including any related processes to promote data quality (eg, duplicate measurements, training of assessors) and a description of study instruments (eg, questionnaires, laboratory tests) along with their reliability and validity, if known. Reference to where data collection forms can be found, if not in the protocol – **Page 8, Line 262-290**
	18b	Plans to promote participant retention and complete follow-up, including list of any outcome data to be collected for participants who discontinue or deviate from intervention protocols - **Page 9, Line 301-303**
Data management	19	Plans for data entry, coding, security, and storage, including any related processes to promote data quality (eg, double data entry; range checks for data values). Reference to where details of data management procedures can be found, if not in the protocol – **Page 13, Line 471-478**
Statistical methods	20a	Statistical methods for analysing primary and secondary outcomes. Reference to where other details of the statistical analysis plan can be found, if not in the protocol – **Page 11, Line 375-401**

	20b	Methods for any additional analyses (eg, subgroup and adjusted analyses) – **Page 11,Line 402-406**
	20c	Definition of analysis population relating to protocol non-adherence (eg, as randomised analysis), and any statistical methods to handle missing data (eg, multiple imputation) – **Page 9, Line 296-303**
**Methods: Monitoring**		
Data monitoring	21a	Composition of data monitoring committee (DMC); summary of its role and reporting structure; statement of whether it is independent from the sponsor and competing interests; and reference to where further details about its charter can be found, if not in the protocol. Alternatively, an explanation of why a DMC is not needed – **Page 10, Line 357-374**
	21b	Description of any interim analyses and stopping guidelines, including who will have access to these interim results and make the final decision to terminate the trial - **Page 10, Line 357-374**
Harms	22	Plans for collecting, assessing, reporting, and managing solicited and spontaneously reported adverse events and other unintended effects of trial interventions or trial conduct - **Page 9, Line 304-328**
Auditing	23	Frequency and procedures for auditing trial conduct, if any, and whether the process will be independent from investigators and the sponsor - **Page 10, Line 357-374**
**Ethics and dissemination**		
Research ethics approval	24	Plans for seeking research ethics committee/institutional review board (REC/IRB) approval – **Page 13, Line 446-454**
Protocol amendments	25	Plans for communicating important protocol modifications (eg, changes to eligibility criteria, outcomes, analyses) to relevant parties (eg, investigators, REC/IRBs, trial participants, trial registries, journals, regulators) **Page 13, Line 446-454**
Consent or assent	26a	Who will obtain informed consent or assent from potential trial participants or authorised surrogates, and how (see Item 32) **Page 13, Line 455-465**
	26b	Additional consent provisions for collection and use of participant data and biological specimens in ancillary studies, if applicable **Page 13, Line 466-469**
Confidentiality	27	How personal information about potential and enrolled participants will be collected, shared, and maintained in order to protect confidentiality before, during, and after the trial **Page 13, Line 446-469**
Declaration of interests	28	Financial and other competing interests for principal investigators for the overall trial and each study site - **Page 3**

For peer review only - http://bmjopen.bmj.com/site/about/guidelines.xhtml

Access to data	29	Statement of who will have access to the final trial dataset, and disclosure of contractual agreements that limit such access for investigators **Page 13, Line 471-478**
Ancillary and post-trial care	30	Provisions, if any, for ancillary and post-trial care, and for compensation to those who suffer harm from trial participation **Not applicable**
Dissemination policy	31a	Plans for investigators and sponsor to communicate trial results to participants, healthcare professionals, the public, and other relevant groups (eg, via publication, reporting in results databases, or other data sharing arrangements), including any publication restrictions **Page 13, Line 453-454**
	31b	Authorship eligibility guidelines and any intended use of professional writers **Page 3**
	31c	Plans, if any, for granting public access to the full protocol, participant-level dataset, and statistical code **Not applicable**
**Appendices**		
Informed consent materials	32	Model consent form and other related documentation given to participants and authorised surrogates – **Supplementary file 1**
Biological specimens	33	Plans for collection, laboratory evaluation, and storage of biological specimens for genetic or molecular analysis in the current trial and for future use in ancillary studies, if applicable **Not applicable**

*It is strongly recommended that this checklist be read in conjunction with the SPIRIT 2013 Explanation & Elaboration for important clarification on the items. Amendments to the protocol should be tracked and dated. The SPIRIT checklist is copyrighted by the SPIRIT Group under the Creative Commons "Attribution-NonCommercial-NoDerivs 3.0 Unported" license.

For peer review only
