# Supplementary material for: Study protocol for two randomised controlled trials evaluating the effects of Cerclage in the reduction of extreme preterm birth and perinatal mortality in twin pregnancies with a short cervix or dilatation: the TWIN Cerclage studies
Source: BMJ Open. 2024 May 10;14(5):e081561. doi: 10.1136/bmjopen-2023-081561 (PMC11097875; doi:10.1136/bmjopen-2023-081561)
Supplement: Reviewer comments [file bmjopen-2023-081561.reviewer_comments.pdf]

PEER REVIEW HISTORY

BMJ Open publishes all reviews undertaken for accepted manuscripts. Reviewers are asked to complete a checklist review form (**http://bmjopen.bmj.com/site/about/resources/checklist.pdf**) and are provided with free text boxes to elaborate on their assessment. These free text comments are reproduced below.

ARTICLE DETAILS

**TITLE (PROVISIONAL)**	Study protocol for two randomized controlled trials evaluating the effect of cerclage in the reduction of extreme preterm birth and perinatal mortality in twin pregnancies with a short cervix or dilatation: the TWIN Cerclage studies
**AUTHORS**	van Gils, Lissa; de Boer, Marjon; Bosmans, Judith; Duijnhoven, Ruben; Schoenmakers, Sam; Derks, J.; Prins, Jelmer; Al-Nasiry, Salwan; Lutke Holzik, Margo; Lopriore, Enrico; van Drongelen, J.; Knol, H.; van Laar, J.; Jacquemyn, Y.; van Holsbeke, Caroline; Dehaene, Isabelle; Lewi, Liesbeth; van der Merwe, Hannes; Gyselaers, Wilfried; Obermann-Borst, Sylvia; Holthuis, Mayella; Mol, Ben; Pajkrt, Eva; Oudijk, Martijn

This article was not externally reviewed at BMJ Open. Protocols that have been independently assessed prior to submission to BMJ Open are usually fast-tracked to publication on the grounds that further substantial changes will not be possible. This independent assessment will usually be external, independent review for both a substantial grant award from a non-commercial or government funder and ethics approval.
